# The E3 Ubiquitin Ligase RLIM Safeguards Oligodendrocyte Development and Myelination by Targeting SLC7A11 for Polyubiquitination to Regulate Ferroptotic Resistance

**DOI:** 10.1002/advs.76315

**Published:** 2026-07-23

**Authors:** Yuwei Li, Haijun Zhang, Jieya Zhou, Shiyu Yang, Jingmei Cha, Gaofeng Yan, Shuhua Zhao, Bingyu Mao, Pengcheng Ma

**Affiliations:** ^1^ State Key Laboratory of Genetic Evolution and Animal Models Kunming Institute of Zoology Chinese Academy of Sciences Kunming Yunnan China; ^2^ Department of Reproduction and Genetics the First Affiliated Hospital of Kunming Medical University Kunming Yunnan China; ^3^ Kunming College of Life Science University of Chinese Academy of Sciences Kunming China; ^4^ National Research Facility for Phenotypic & Genetic Analysis of Model Animals (Primate Facility) Kunming Institute of Zoology Chinese Academy of Sciences Kunming Yunnan China

**Keywords:** ferroptosis, myelination, OPC, RLIM, SLC7A11, ubiquitination

## Abstract

In the central nervous system, oligodendrocytes (OLs) generate myelin sheaths to support rapid nerve impulse conduction. OL lineage cells, especially oligodendrocyte precursor cells (OPCs), feature high metabolic activity and are exposed to severe oxidative stress, but the protective mechanisms remain poorly understood. Here we show that RLIM, an E3 ubiquitin ligase linked to X‐linked neurodevelopmental disorders, safeguards OL development via ferroptosis resistance. RLIM directly polyubiquitinates SLC7A11, a key cystine/glutamate antiporter for glutathione (GSH) synthesis, thereby sustaining SLC7A11 membrane localization. OL lineage‐specific ablation of RLIM in mice reduces membrane SLC7A11, impairs OPC proliferation, and triggers ferroptosis and thus myelination defects, leading to motor, social, and cognitive deficits that mimic patient phenotypes. Most pathological RLIM missense variants disrupt SLC7A11 binding and/or polyubiquitination. Importantly, GSH supplementation rescues myelination defects and behavioral abnormalities in RLIM‐deficient mice. These findings reveal that the RLIM–SLC7A11‐GSH signaling axis governs ferroptosis resistance in OL lineage cells, implicates this pathway in RLIM‐related neurodevelopmental disorders, and suggests GSH as a potential therapeutic strategy.

## Graphical abstract

RLIM‐mediated SLC7A11 polyubiquitination is required for SPTBN2‐mediated trafficking and membrane localization of SLC7A11, which enables oligodendrocyte precursor cells to be resistant to ferroptosis and safeguards oligodendrocyte lineage progression and thus myelination. GSH administration restores the defects in oligodendrocyte lineage development and myelination, and behavioral abnormalities in mice with *RLIM* knockout in oligodendrocyte lineage cells.

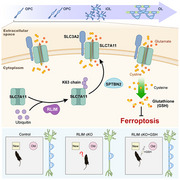

## 1 Introduction

Myelin sheaths wrapped around the nerve axons are necessary to rapidly and efficiently conduct action potentials in the central nervous system (CNS) [1]. Myelination is a dynamic process involved in brain homeostasis throughout the whole life, deregulation of which is related to a variety of neurological diseases, including neurodevelopmental disorders, neurodegenerative diseases, and neuropsychiatric problems [1, 2, 3, 4]. In the CNS, myelin is supplied by oligodendrocytes (OLs). During development, OL lineage cells, identified from neural stem cells, progress through a series of stages, including immature (pre‐myelinating) OL (iOL) and mature myelinating OL (mOL), beginning with the specification of oligodendrocyte precursor cells (OPCs) [5, 6]. Unlike other neuroglia, such as astroglia and microglia, whose dedicated precursors mainly disappear in postnatal brains, PDGFRα^+^ OPCs form a sizable and stable population in the adult brain and bear the ability to differentiate into CC1^+^ iOL and MBP^+^ OL (mOL) throughout the whole life [7, 8, 9]. Emerging evidence suggests that adult‐born OLs play an integral part in activity‐dependent adaptive myelination, which is thought to play a significant role in neuroplasticity, learning, and memory [10, 11, 12].

OPCs and OLs are thought to be metabolically and energetically active [13]. During OL development, OPCs undergo as much as a 6,500‐fold increase in membrane area to provide myelin segments for multiple axons wrapping, a process that also entails extraordinary metabolic demands [14, 15]. Oligodendrocytes can accommodate up to 40 myelin segments and maintain membrane extensions up to 100 × the weight of their cell body [16, 17]. In addition to providing myelin for neuronal axons to support rapid action potentiation transduction, OL is the main source of nutritional support for the surrounding neurons [18]. Adaptively, in OL lineage cells, there is a considerable amount of tubular mitochondria to sustain high oxidative phosphorylation, and consistently, OPCs harbour a very high oxygen consumption and ATP production rate [8, 19, 20, 21]. Recently, compared to the other neuroglia cell types, OL lineage cells were reported to be the largest cell population in the brain and thought to be the major consumers of energy among all CNS cells [8, 22]. Mounting studies have focused on mechanisms regulating OL development and myelination; however, little is known about how OL lineage cells adaptively cope with such high oxidative stress.

Ferroptosis is a regulated form of cell death induced by accumulating lipid reactive oxygen species (ROS) such as lipid hydroperoxides [23, 24]. Lipid peroxidation is the most important marker and must be detected to distinguish ferroptosis from other forms of cell death [25, 26]. The cystine/glutamate antiporter SLC7A11 (also known as xCT) is used to import cystine for glutathione (GSH) biosynthesis and antioxidant defense [27]. As one of the most important ferroptosis‐defending systems, SLC7A11 normally functions as a strong ferroptosis suppressor together with GPX4 to reduce reactive polyunsaturated fatty acid (PUFA) phospholipid hydroperoxides to non‐reactive and non‐lethal PUFA phospholipid alcohols [28]. To ensure that SLC7A11 can properly maintain redox homeostasis, whose expression is precisely regulated through multiple mechanisms at the levels of epigenetic modification, transcription, translation, and post‐translational modification [29, 30, 31, 32, 33, 34, 35]. In the CNS, OL lineage cells, including OPCs and OLs, express a high level of SLC7A11, and pharmacological inhibition of SLC7A11 leads to reduced expression of myelin genes and thus hypomyelination in mice [36, 37, 38, 39]. *SLC7A11* knockout mice are more tolerant of LPS‐induced inflammation and depressive like behavior and chemically induced tumorigenesis in mice [40, 41]. However, the mechanism by which SLC7A11 regulates oligodendrocyte development and myelination remains to be investigated. Although increasing evidence suggests that ferroptosis is an essential form of OPC cell death in pathological conditions, and rescuing ferroptotic OPCs could serve as a therapeutic strategy for remyelination defects [42, 43], whether ferroptosis is involved in oligodendrocyte development and myelination under physiological conditions is largely unknown.

Ubiquitination is a critical post‐translational modification regulating diverse biological processes [44]. The E3 ubiquitin ligases were responsible for specific substrate recognition and targeting, serving as regulatory gatekeepers of myriad biological processes [45]. Therefore, deregulation or mutation of E3 ubiquitin ligases has been implicated in many human diseases [45, 46]. This is exemplified by the E3 ubiquitin ligase RLIM, encoded by the X‐linked gene *RLIM* (*RNF12*), whose mutations cause Tonne‐Kalscheuer syndrome (TOKAS, OMIM: 300978), a class of neurodevelopmental disorders characterized by global developmental delay, impaired intellectual development, speech delay, behavioral abnormalities, and abnormal gait [47, 48, 49, 50, 51, 52]. Brain imaging examination showed widespread white matter abnormalities in *RLIM‐*affected patients, implying an important role of RLIM in OL development and myelination [49]. Although studies support that RLIM targets the transcriptional regulator REX1 for ubiquitination and proteasomal degradation, through which RLIM regulates specific gene expression programs involved in X‐chromosome inactivation (XCI) and gametogenesis [53, 54, 55, 56, 57, 58], the underlying mechanism of RLIM in neural development, especially OL differentiation and myelination, and the etiology of RLIM‐affected neurodevelopmental disorders are still elusive.

Here, we report a critical role of RLIM‐mediated ferroptotic resistance in OL development, myelination, and remyelination in mice. Specific depletion of *RLIM* in OL lineage cells impairs OPC proliferation and differentiation, and induces OPC ferroptosis by downregulating the membrane SLC7A11 level via a ubiquitination‐dependent manner. The mice with specific deletion of *RLIM* in OL lineage cells exhibit motor coordination defects, social, learning, and memory impairments, phenocopying the symptoms of *RLIM*‐affected patients. Furthermore, we found that GSH administration restored the defects in OL development, myelination, remyelination, and impaired cognitive function in the *RLIM*‐depleted mice, suggesting a potential therapeutic strategy for *RLIM*‐affected neurodevelopmental disorders.

## 2 Results

### 2.1 *RLIM* Is Required for OL Development and Myelination

To examine the expression of *RLIM* in OL lineage cells, OPCs and differentiated OLs were purified with either platelet‐derived growth factor receptor α (PDGFRα) or OL cell surface antigen 4 (O4) beads from P10 mouse cortical tissues [59, 60]. Reverse transcription coupled with quantitative polymerase chain reaction (RT‐qPCR) results showed that there is a significant enrichment for the expression of *RLIM* in OL lineage cells, including OPCs and OLs (Figure S1A–C). Similarly, immunofluorescence (IF) staining on corpus callosum sections of P7 or P14 mouse brains showed that RLIM was mainly observed in the cytoplasm of PDGFRα^+^ OPCs and CC1^+^ OLs (Figure S1D,E). Moreover, the expression of RLIM coincides with that of MBP, a myelin‐related protein, which is a marker for mature OL (Figure S1F). Together, these observations suggest that RLIM have a faithful expression pattern in OL lineage cells during mouse development, indicating that RLIM might have a regulatory role in oligodendrocyte development and myelination in mice.

To investigate how RLIM influences myelination in the central nervous system (CNS), we ablated *RLIM* in OL lineage cells by breeding mice with the *RLIM* floxed allele (*RLIM^flox/flox^*) with an oligodendrocyte lineage expression *Olig2‐Cre* line that commences in primitive OPCs [61, 62]. We compared conditional *RLIM* knockout mice (*RLIM^fl/y^;Olig2‐Cre*, hereafter referred to as *RLIM* cKO) with their littermate controls (*RLIM^wt/y^;Olig2‐Cre*, hereafter referred to as control). *RLIM* knockout was first confirmed by RT‐qPCR and IF assays using cortical tissues of P14 mouse brains, and the results showed a significant reduction in *RLIM* mRNA level and the number of RLIM^+^SOX10^+^ cells (Figure S2A–C). Further, we observed that the *RLIM* cKO mice were born at the Mendelian ratio, and there was no significant difference in survival rate, general appearance, brain morphology, and cortical patterning at adult age between the *RLIM* cKO and control mice (Figure S2D–G).

**FIGURE 1:**
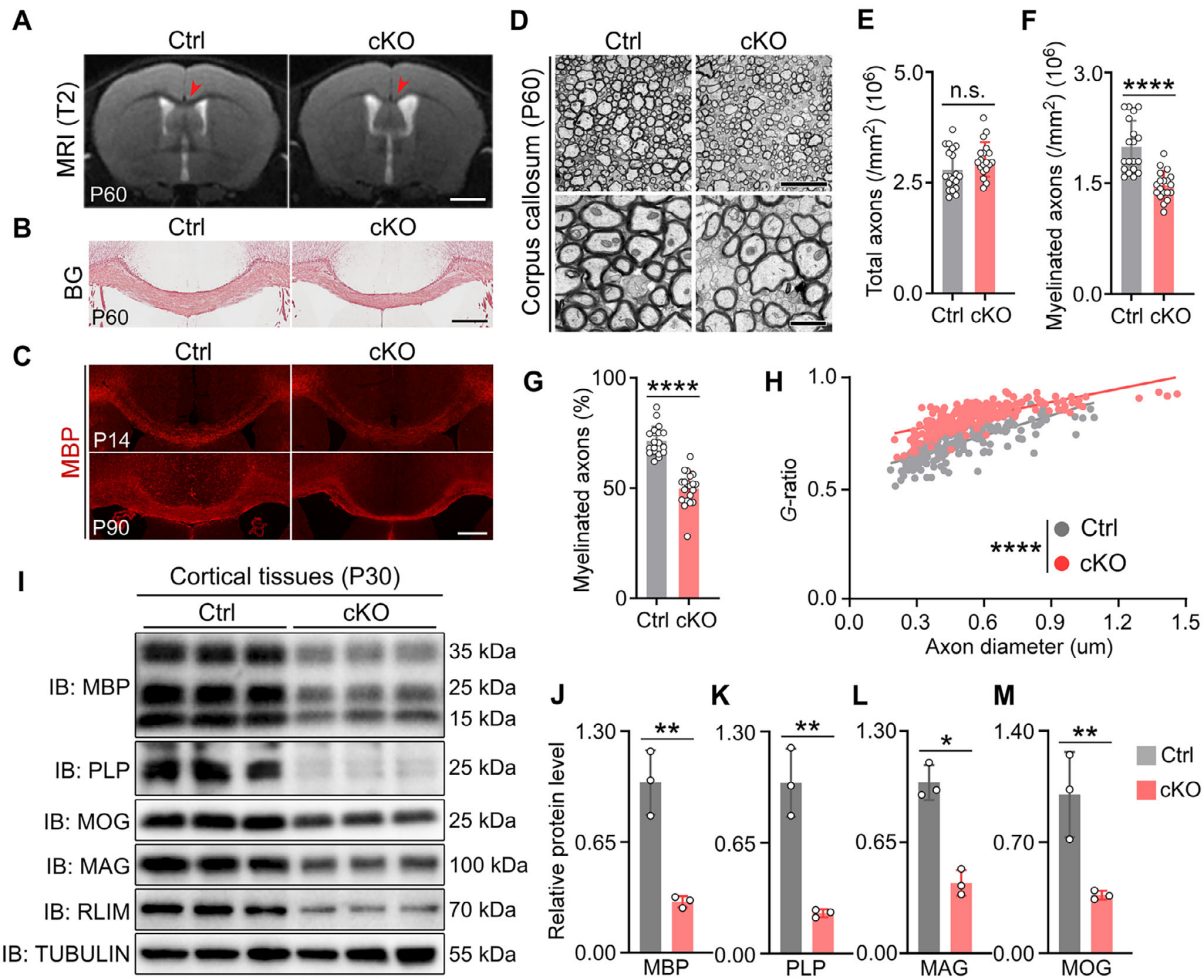
*RLIM* knockout in OL lineage cells leads to hypomyelination in mouse brains. (A) Representative T2‐weighted mouse brain MRI coronal scanning of P60 control (*n* = 3) and the *RLIM* cKO (*n* = 3) mice. The red arrows indicate the corpus callosum. Scale bar, 2 mm. (B) Representative images of Black‐Gold staining (BG) showing myelin staining in corpus callosum regions of P60 control (*n* = 3) and the *RLIM* cKO (*n* = 3) mice. Scale bar, 500 µm. (C) Representative images of immunofluorescence staining assays showing the expression of MBP in the corpus callosum of control (P14: *n* = 3, P90: *n* = 3) and the *RLIM* cKO (P14: *n* = 3, P90: *n* = 3) mice at the indicated stages. Scale bar, 400 µm for both P14 and P90. (D) Transmission electron microscopy (TEM) images of the corpus callosum transverse sections from P60 control (*n* = 3) and the *RLIM* cKO (*n* = 3) mice. Scale bars: 5 µm for the upper panels and 1 µm for the lower panels. (E) Bar graphs (mean ± SD) showing quantification of the total axon number in (D) (control, *n* = 3, 2,790,315 ± 465,322 /mm^2^ and RLIM‐cKO, *n* = 3, 3,026,587 ± 391,517 /mm^2^). Unpaired two‐tailed Student's *t*‐test; n.s., not significant. (F) Bar graphs (mean ± SD) showing quantification of the number of myelinated axons in (D) (control, *n* = 3, 1,994,203 ± 353,549 /mm^2^ and *RLIM* cKO, *n* = 3, 1,471,316 ± 191,561 /mm^2^). Unpaired two‐tailed Student's *t*‐test; ^****^, *p* < 0.0001. (G) Bar graphs (mean ± SD) show quantification of the percentage of myelinated axons in (D) (Control, *n* = 3, 71.36 ± 6.439% and *RLIM* cKO, *n* = 3, 49.69 ± 7.715%). Unpaired two‐tailed Student's *t*‐test. ^****^, *p* < 0.0001. (H) Scatterplots show the myelin *g*‐ratios (diameter of axon/diameter of entire fiber). At least 200 axons from 3 mice were involved in the analysis for each group. General linear regression model and ANCOVA analysis; ^****^; *p* < 0.0001. (I) WB analysis of expression levels of the myelin‐related proteins, including MBP, PLP, MAG, and MOG, in the corpus callosum of P30 control (*n* = 3) and the *RLIM* cKO (*n* = 3) mice. (J–M) Bar graphs (mean ± SD) showing quantification of the relative protein levels of MBP (J, control: 1.000 ± 0.1893 and *RLIM* cKO: 0.2990 ± 0.03274), PLP (K, control: 1.000 ± 0.1993 and *RLIM* cKO: 0.2371 ± 0.02297), MAG (L, control: 1.000 ± 0.2674 and *RLIM* cKO: 0.3664 ± 0.02787), and MOG (M, control: 1.000 ± 0.1021 and *RLIM* cKO: 0.4117 ± 0.07572) in (I). TUBULIN was used as an internal control, and the indicated protein levels in the control group were set to 1. Unpaired two‐tailed Student's *t*‐test; *, *p* < 0.05; **, *p* < 0.01. Ctrl, control; cKO, *RLIM* cKO; BG, black‐gold staining.

T2‐weighted magnetic resonance imaging (MRI) analysis was used to examine the white matter volume of the corpus callosum in the *RLIM* cKO and control mice. The results showed that the signal intensity was specifically and significantly increased in adult *RLIM* cKO mice as compared to their littermate controls, suggesting a myelination defect in the brains of the *RLIM* cKO mice (Figure 1A; Figure S2H). The black‐gold (BG) staining was then used to examine the axonal fibers of the corpus callosum in these mice, where consistently severe hypomyelination was found in the *RLIM* cKO mice (Figure 1B). Moreover, a significant reduction in MBP IF staining signals in corpus callosum tissues of P14 and adult mouse brains, which further confirmed the defects in myelination in the *RLIM* cKO mice (Figure 1C). Next, when transmission electron microscopy (TEM) imaging was applied to analyze the myelin ultrastructure, although the number of total axons in the corpus callosum was not changed, the number and percentage of myelinated axons, and myelin thickness were specifically and markedly reduced in the corpus callosum of the adult *RLIM* cKO mice (Figure 1D–H). Accordingly, both the mRNA and protein levels of myelin‐related molecules, including MBP, PLP, MAG, and MOG, were significantly reduced in cortical tissues of the *RLIM* cKO mice at P30 (Figure 1I–M; Figure S2I–M). Together, these results indicate that RLIM is required for CNS myelination in mice.

**FIGURE 2:**
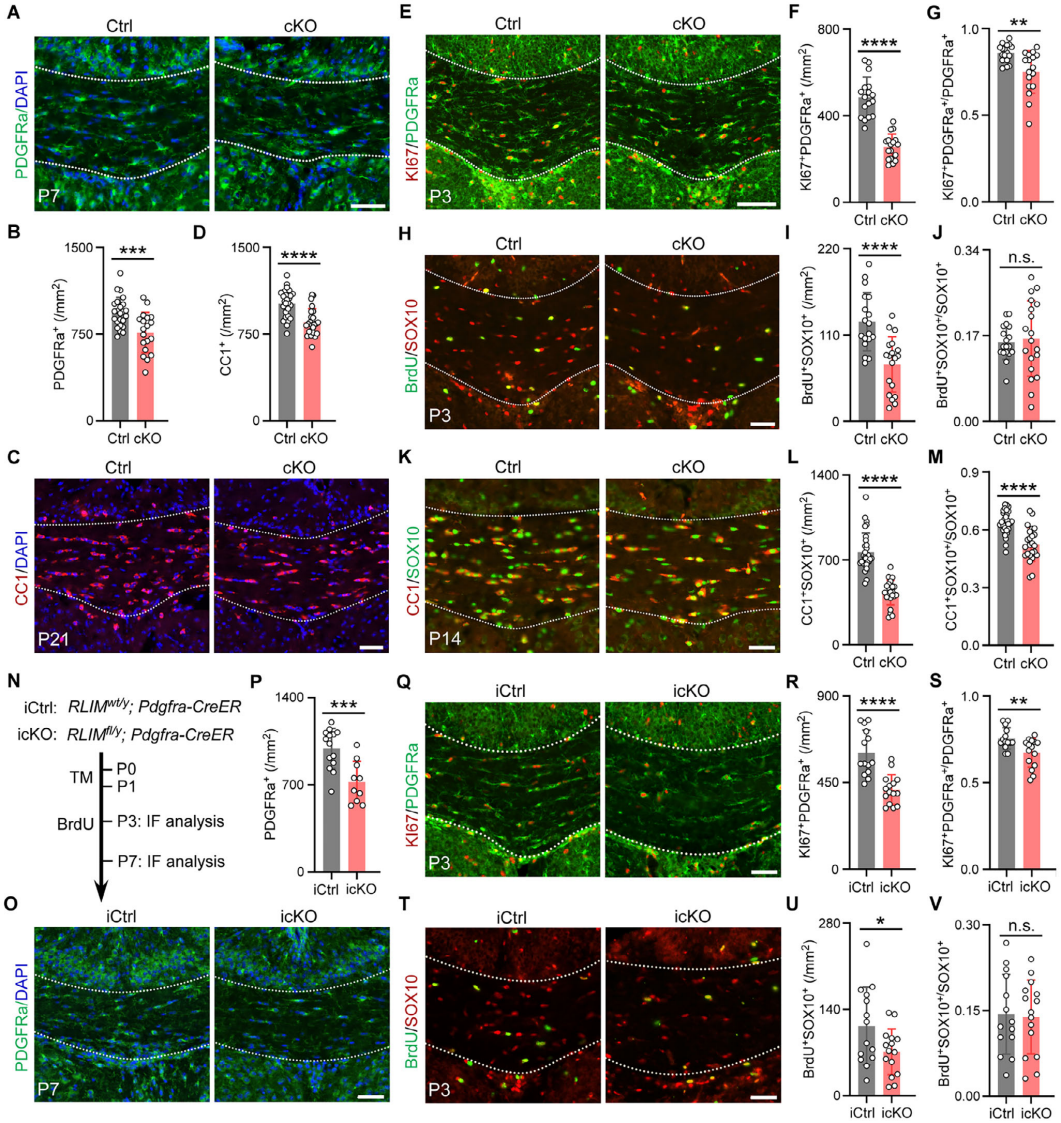
*RLIM* knockout impairs oligodendroglial development in mouse brains. (A) Representative images of immunofluorescence staining assays showing PDGFRα expression in the corpus callosum of control (*n* = 3) and the *RLIM* cKO (*n* = 3) mice at P7. Scale bar, 50 µm. (B) The bar graph shows the quantification of the number of PDGFRα^+^ OPC cells in (A) (Control: 936.6 ± 131.5 /mm^2^ and *RLIM* cKO: 763.9 ± 174.4 /mm^2^). Unpaired two‐tailed Student's *t*‐test; ^***^, *p* < 0.001. (C) Representative images of immunofluorescence staining assays showing CC1 expression in the corpus callosum of control (*n* = 3) and the *RLIM* cKO (*n* = 3) mice at P21. Scale bar, 50 µm. (D) The bar graph shows the quantification of the number of CC1^+^ OL cells in (C) (control: 1,013 ± 120.2 /mm^2^ and *RLIM* cKO: 853.2 ± 117.1 /mm^2^). Unpaired two‐tailed Student's *t*‐test; ^****^, *p* < 0.0001. (E) Representative images of immunofluorescence staining assays showing KI67 and PDGFRα expression in the corpus callosum of control (*n* = 3) and the *RLIM* cKO (*n* = 3) mice at P3. Scale bar, 100 µm. (F) The bar graph shows the quantification of the number of KI67^+^PDGFRα^+^ cells in (E) (control: 485.1 ± 92.90 /mm^2^ and *RLIM* cKO: 255.9 ± 59.43 /mm^2^). Unpaired two‐tailed Student's *t*‐test; ^****^, *p* < 0.0001. (G) The bar graph shows the quantification of the ratio of KI67^+^PDGFRα^+^ among PDGFRα^+^ cells in (E) (control: 0.8585 ± 0.05155 and *RLIM* cKO: 0.7493 ± 0.1218). Unpaired two‐tailed Student's *t*‐test; ^**^, *p* < 0.01. (H) Representative images of immunofluorescence staining coupled with BrdU pulse labeling assays showing BrdU labeling and SOX10 expression in the corpus callosum of control (*n* = 3) and *RLIM* cKO (*n* = 3) mice at P3. Scale bar, 50 µm. (I) The bar graph shows the quantification of the number of BrdU^+^SOX10^+^ cells in (H) (control: 126.9± 37.04 /mm^2^ and *RLIM* cKO: 72.59 ± 35.28 /mm^2^). Unpaired two‐tailed Student's *t*‐test, ^****^, *p* < 0.0001. (J) The bar graph shows the quantification of the ratio of BrdU^+^SOX10^+^ among SOX10^+^ cells in (H) (control: 0.1570 ± 0.03410 and *RLIM* cKO: 0.1645 ± 0.07476). Unpaired two‐tailed Student's *t*‐test; n.s., not significant. (K) Representative images of immunofluorescence staining assays showing CC1 and SOX10 expressions in the corpus callosum of control (*n* = 3) and the *RLIM* cKO (*n* = 3) mice at P14. Scale bar, 50 µm. (L) The bar graph shows the quantification of the number of CC1^+^SOX10^+^ cells in (K) (control: 765.1 ± 157.6 /mm^2^ and *RLIM* cKO: 431.1 ± 102.0 /mm^2^). Unpaired two‐tailed Student's *t*‐test; ^****^, *p* < 0.0001. (M) The bar graph shows the quantification of the ratio of CC1^+^SOX10^+^ among SOX10^+^ cells in (K) (control: 0.6404 ± 0.06082 and *RLIM* cKO: 0.5242 ± 0.08713). Unpaired two‐tailed Student's *t*‐test; ^****^, *p* < 0.0001. (N) Schematic diagram of tamoxifen‐induced knockout and BrdU labeling in iCtrl and *RLIM* iKO mice. (O) Representative images of immunofluorescence staining assays showing PDGFRα expression in the corpus callosum of iCtrl (*n* = 3) and the *RLIM* icKO (*n* = 3) mice at P7. Scale bar, 50 µm. (P) The bar graph shows the quantification of the number of PDGFRα^+^ OPC cells in (O) (iCtrl: 992.9 ± 160.8 /mm^2^ and *RLIM* icKO: 533.0 ± 167.4 /mm^2^). Unpaired two‐tailed Student's *t*‐test; ^***^, *p* < 0.001. (Q) Representative images of immunofluorescence staining assays showing KI67 and PDGFRα expression in the corpus callosum of iCtrl (*n* = 3) and the *RLIM* icKO (*n* = 3) mice at P3. Scale bar, 100 µm. (R) The bar graph shows the quantification of the number of KI67^+^PDGFRα^+^ cells in (Q) (iCtrl: 604.2 ± 118.8 /mm^2^ and *RLIM* icKO: 413.6 ± 77.04 /mm^2^). Unpaired two‐tailed Student's *t*‐test; ^****^, *p* < 0.0001. (S) The bar graph shows the quantification of the ratio of KI67^+^PDGFRα^+^ among PDGFRα^+^ cells in (Q) (iCtrl: 0.7549 ± 0.06223 and *RLIM* icKO: 0.6727 ± 0.08323). Unpaired two‐tailed Student's *t*‐test; ^**^, *p* < 0.01. (T) Representative images of immunofluorescence staining coupled with BrdU pulse labeling assays showing BrdU labeling and SOX10 expression in the corpus callosum of iCtrl (*n* = 3) and *RLIM* icKO (*n* = 3) mice at P3. Scale bar, 50 µm. (U) The bar graph shows the quantification of the number of BrdU^+^SOX10^+^ cells in (T) (iCtrl: 113.1± 62.98 /mm^2^ and *RLIM* icKO: 71.14 ± 37.10 /mm^2^). Unpaired two‐tailed Student's *t*‐test; ^*^; *p* < 0.05. (V) The bar graph shows the quantification of the ratio of BrdU^+^SOX10^+^ among SOX10^+^ cells in (T) (iCtrl: 0.1436 ± 0.06968 and *RLIM* icKO: 0.1390 ± 0.06453). Unpaired two‐tailed Student's *t*‐test; n.s., not significant. Ctrl, Control; cKO, *RLIM* cKO; iCtrl, iControl; icKO, *RLIM* icKO.

To investigate whether the hypomyelination phenotype in the *RLIM* cKO mice is due to a defect in OL development, we assessed OPC development by immunostaining assays on corpus callosum sections at different developmental stages. First, we counted the number of PGGFRα^+^ OPCs and CC1^+^ OLs in the corpus callosum at P7 or P21, respectively, in the *RLIM* cKO animals and found that both the number of OPCs and OLs was reduced significantly (Figure 2A–D), indicating a defect in OL development in the brains of the *RLIM* cKO mice. Next, OPC proliferation was examined by BrdU pulse labeling and KI67 co‐staining analyses at P3, a developmental stage with the highest OPC proliferating rate, and the results showed that both the number and ratio of proliferating OPCs were reduced in the corpus callosum after *RLIM* knockout (Figure 2E–J). Co‐immunostaining of the OPC marker SOX10 and the OL marker CC1 was used to analyze OL differentiation, and we further found that the number and ratio of differentiating OPCs (CC1^+^Sox10^+^) were significantly decreased in the corpus callosum of the *RLIM* cKO mice at P14 (Figure 2K–M). It is known that there is a chronological procedure of oligodendroglia development in postnatal mouse brains: before P7 OPCs are mainly proliferated, and after that, OL cells are differentiated. Therefore, to examine whether the impaired OL differentiation after *RLIM* deletion is a secondary effect of the early deregulated proliferation, *RLIM^flox/flox^* mice were crossed with the *PDGFRα‐CreER* transgenic mouse line, an OPC‐specific tamoxifen‐inducible Cre line [63], to obtain the two mouse lines (*RLIM^flox/y^; PDGFRα‐CreER*, *RLIM‐icKO* and *RLIM^wt/y^; PDGFRα‐CreER, RLIM‐iCtrl*). The *RLIM‐icKO* mice, as well as the control group, were treated with tamoxifen to induce *RLIM* deletion at different developmental stages. At first, the *RLIM‐icKO* mice were treated with tamoxifen on P0 and P1 to induce *RLIM* deletion from birth (Figure 2N), and the brain samples were harvested on P3 after BrdU injection for 2 hours. Double immunostaining confirmed RLIM loss in PDGFRα^+^ cells in the corpus callosum of P3 *RLIM*‐icKO mouse brains (Figure S3A,B). In addition, the results showed that the number of OPCs labeled by PDGFRα^+^ and proliferating OPCs marked by Sox10^+^BrdU^+^ or PDGFRα^+^Ki67^+^ was severely decreased in the *RLIM‐icKO* mice (Figure 2O–V), confirming that RLIM is involved in regulating OPC proliferation at the early developmental stage. Then, tamoxifen was administered to the *RLIM‐icKO* mice from P8 to P10, and OL differentiation was examined in the brains of P14 or P21 mice (Figure S3C). Immunostaining assays also confirmed RLIM loss in the OL lineage cells in the corpus callosum of P14 *RLIM*‐icKO mouse brains (Figure S3D,E). Interestingly, the number of CC1^+^ OL cells in P21 brains and the number of CC1^+^Sox10^+^ differentiating OL cells in P14 brains of the *RLIM*‐icKO mice were both comparable to those in brains of the *RLIM*‐iCtrl mice (Figure S3F–J). Consistently, there is no significant difference in the expression level of MBP and axon myelination in P21 brains between *RLIM*‐iCtrl and *RLIM*‐icKO mice (Figure S3K–P). These data suggest that RLIM loss has a limited direct effect on OL differentiation per se, whereas the pronounced differentiation defect in constitutive cKO mice is probably secondary to the reduced OPC pool resulting from impaired proliferation. Taken together, these results indicate that RLIM is directly involved in regulating OPC proliferation, while its deficiency has an impact on OL development and leads to hypomyelination in mouse brains.

### 2.2 Deficiency of *RLIM* in OL Lineage Cells Leads to Motor Coordination Defects, Social, Learning, and Memory Impairments in Mice

**FIGURE 3:**
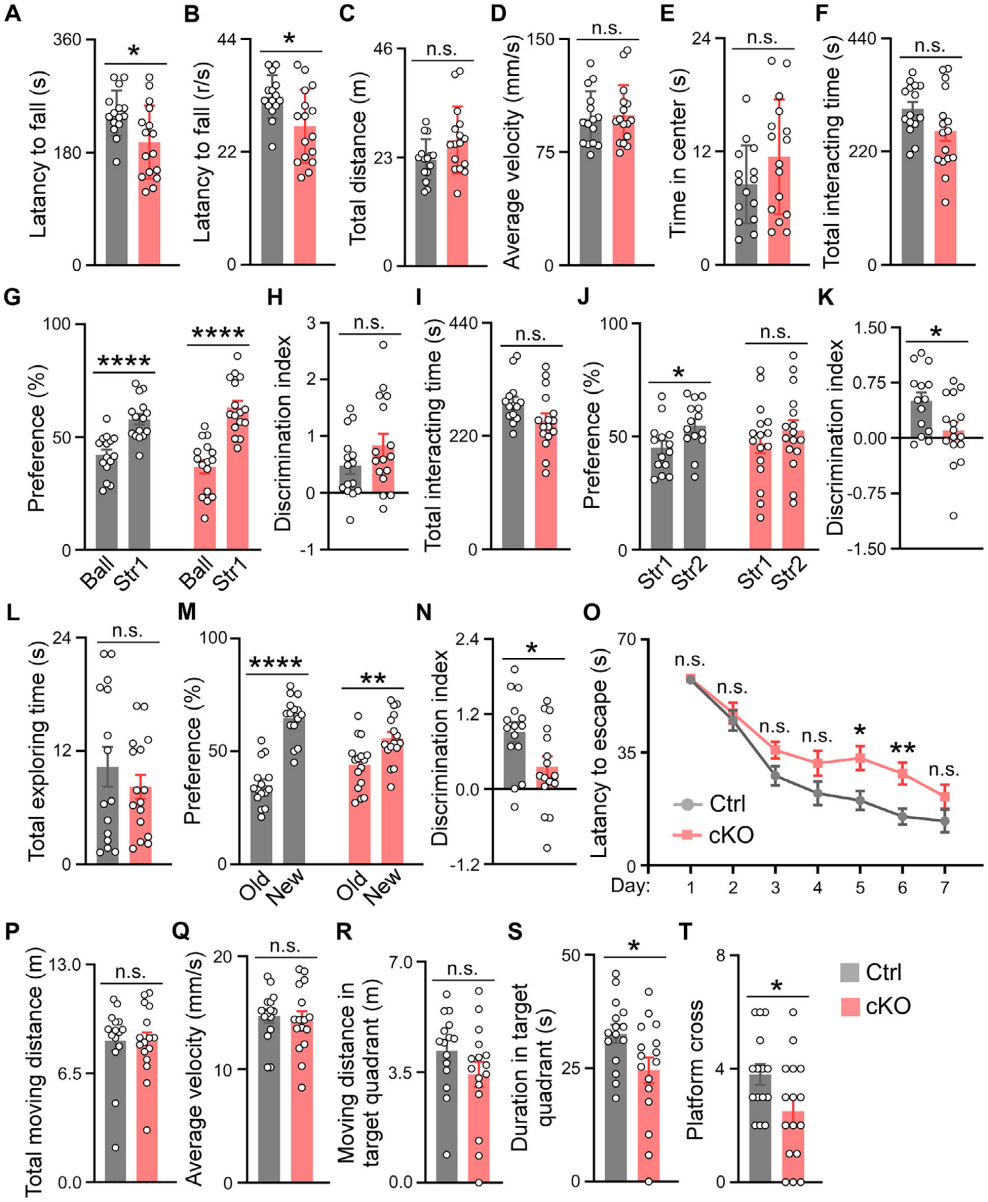
RLIM deficiency in OL lineage cells causes impairment of motor, social, learning, and memory behaviors. (A, B) Mouse performances in the rotarod test (control, n = 15 and *RLIM* cKO, n = 16), including latent time (mean ± SD, control: 242.9 ± 36.11 s and *RLIM* cKO: 196.6 ± 58.58 s) on a running rotarod (A) and the maximal rate (mean ± SD, Control: 32.73 ± 4.200 r/s and *RLIM* cKO: 27.00 ± 7.137 r/s) when mice fell on an accelerated rotarod (B). Unpaired two‐tailed Student's *t*‐test; p = 0.0136; Cohen's d = 0.85, post‐hoc power = 0.72 (A) and p = 0.0114, Cohen's d = 0.90, post‐hoc power = 0.76 (B). (C‐E) Mouse performances in open field tests (control, *n* = 15 and *RLIM* cKO, *n* = 16), including total moving distance (C) (mean ± SD, control: 22,524 ± 4,373 mm and *RLIM* cKO: 26,673 ± 7,081 mm), average velocity (D) (mean ± SD, control: 97.32 ± 18.02 mm/s and *RLIM* cKO: 99.55 ± 19.86 mm/s), and time in center (E) (mean ± SD, control: 8.530 ± 4.111 s and *RLIM* cKO: 11.45 ± 6.086 s). Unpaired two‐tailed Student's *t*‐test; n.s., not significant. (F–K) Mouse performances in the three‐chamber social test (control, *n* = 15 and *RLIM* cKO, *n* = 16). (F) The bar graph (mean ± SD) shows the total interacting time close to the inanimate ball and the stranger mouse (control: 303.4 ± 48.90 s and *RLIM* cKO: 260.2 ± 77.31 s). Unpaired two‐tailed Student's *t*‐test. The bar graph (G) (mean ± SD) shows the percentage of interacting time to the inanimate ball and stranger mouse (control: ball, 42.11 ± 9.153%; stranger, 57.89 ± 9.153%; and *RLIM* cKO: ball, 36.89 ± 11.94%; stranger, 63.11 ± 11.94%). The bar graph (H) (mean ± SD) shows the logarithm of social ratio in (G) (control: 0.4774 ± 0.5620 and *RLIM* cKO: 0.8382 ±0.8134). The bar graph (I) (mean ± SD) shows the total interacting time close to the familiar and stranger mice (control: 285.3 ± 42.06 s and *RLIM* cKO: 249.7 ± 56.54 s). The bar graph (J) (mean ± SD) shows the percentage of interacting time with the familiar and stranger mouse (control: familiar, 45.17 ± 11.03%; stranger, 54.83 ± 11.03%; and *RLIM* cKO: familiar, 47.28 ± 17.87%; stranger, 52.72 ± 17.87%). (K) The bar graph (mean ± SD) shows the logarithm of social ratio in (J) (control: 0.5043 ± 0.4265 and *RLIM* cKO: 0.1012 ± 0.4658). Unpaired two‐tailed Student's *t*‐test; n.s., not significant; ^*^, *p* < 0.05; ^****^, *p* < 0.0001. (L–N) Mouse performance in the novel object recognition test (control, *n* = 15 and *RLIM* cKO, *n* = 16). The bar graph (L) (mean ± SD) shows the total exploring time (control: 10.33 ± 8.158 s and *RLIM* cKO: 8.220 ± 5.087 s) close to new and old objects. The bar graph (M) (mean ± SD) shows the percentage of exploring time close to the old and new objects (control: old, 35.21 ± 9.683%; new, 64.79 ± 9.683%; and *RLIM* cKO: old, 44.12 ± 11.03%; new, 55.88 ± 11.03%). The bar graph (N) (mean ± SD) shows the logarithm of the discrimination index (control: 0.9128 ± 0.6169 and *RLIM* cKO: 0.3570 ± 0.6693). Unpaired two‐tailed Student's *t*‐test; n.s., not significant; ^*^, *p* < 0.05; ^**^, *p* < 0.01; ^****^, *p* < 0.0001. (O–T) Mouse performances in the Morris water maze test (control: *n* = 15 and *RLIM* cKO: *n* = 16). (O) Escape latencies (mean ± SEM) to find the platform throughout the 7‐day learning trials (control: day 1, 57.53 ± 0.8418 s; day 2, 44.98 ± 0.6201 s; day 3, 27.80 ± 0.05718 s; day 4, 22.36 ± 0.09216 s; day 5, 20.13 ± 0.01034 s; day 6, 15.16 ± 0.00364 s; day 7, 13.78 ± 0.1489 s and *RLIM* cKO: day 1, 57.92 ± 1.366 s; day 2, 47.23 ± 3.202 s; day 3, 35.73 ± 2.63 s; day 4, 31.67 ± 3.869 s; day 5, 33.2708 ± 3.733 s; day 6, 28.4583 ± 3.368 s; day 7, 21.3542 ± 3.712 s). (P‐T) Spatial memory retrieval of these mice used in (O) was examined when the platform was removed. The bar graph (P) (mean ± SD) shows the total moving distance during spatial memory tests (control: 8,461 ± 2,273 cm and *RLIM* cKO: 8,428 ± 2,092 cm). The bar graph (Q) (mean ± SD) shows the average moving velocity during spatial memory tests (control: 147.2 ± 22.92 cm/s and *RLIM* cKO: 143.9 ± 29.25 cm/s). The bar graph (R) (mean ± SD) shows the average moving velocity during spatial memory tests (control: 147.2 ± 22.92 cm/s and *RLIM* cKO: 143.9 ± 29.25 cm/s). The bar graph (S) (mean ± SD) shows the duration in the target quadrant during spatial memory tests (control: 32.59 ± 7.713 s and *RLIM* cKO: 24.56 ± 11.44 s). The bar graph (T) (mean ± SD) shows the number of target crosses during spatial memory tests (control: 3.800 ± 1.424 and *RLIM* cKO: 2.500 ± 1.826). Unpaired two‐tailed Student's *t*‐test; n.s., not significant; ^*^, *p* < 0.05; ^**^, *p* < 0.01. Ctrl, control; cKO, *RLIM* cKO.

It is known that myelin dysfunction has a profound effect on neurological functions and psychiatric conditions, including motor coordination, learning and memory, and anxiety, and *RLIM*‐affected patients have symptoms of developmental delays, brain abnormality, and intellectual disability [10, 49]. To determine whether neurological deficits and psychiatric problems are present in the *RLIM* cKO mice, we performed a battery of behavioral tests. Motor coordination was first assessed through rotarod and revealed a slight but significant reduction in the performance of rotarod in the *RLIM* cKO mice, compared to control mice (Figure 3A,B). Then the spontaneous locomotion and anxiety level were assessed using the open‐field test, and we detected no significant differences in the travel distance, average velocity, or time in center between the two groups, suggesting normal spontaneous locomotion and no anxiety‐like behavior in the *RLIM* cKO mice (Figure 3C–E). During the three‐chamber test, which examines social interaction and memory, we found that the *RLIM* cKO mice showed a significant preference for the animated stranger over the inanimate ball to the same extent as their littermate controls (Figure 3F–H). However, the *RLIM* cKO mice had a significantly lower preference for the new stranger over the familiar stranger compared to the controls (Figure 3I–K), suggesting an impairment of social memory. Next, the non‐spatial and spatial memory were examined by the novel object recognition and Morris water maze tests, respectively. In the novel object recognition test, the *RLIM* cKO mice showed less preference for the new object as compared to their control littermates (Figure 3L–N), suggesting that non‐spatial memory is impaired with *RLIM* deficiency. In the Morris water maze test, we found that the *RLIM* cKO mice took more time to find the hidden platform on the 5th and 6th days during the 7‐day training (Figure 3O), suggesting a higher escape latency and impairment of spatial learning. One day after the training trials, spatial memory was tested. Similarly, it was shown that there was no significant difference in the total movement distances or average velocity between the control and *RLIM* cKO mice, suggesting that *RLIM* knockout in OL lineage cells had no effect on motor function (Figure 3P,Q). Although there is no difference in the moving distances in the target quadrant between the two groups, the *RLIM* cKO mice spent significantly less time in the target quadrant and had severely decreased crosses for the original target zone (Figure 3R–T). Together, these results indicate that spatial learning and memory are impaired in the *RLIM* cKO mice. Collectively, these results indicate that *RLIM* deficiency in OL lineage cells leads to defects in motor coordination, social behavior, and learning and memory, phenocopying the neurological and psychiatric malfunctions in *RLIM*‐affected patients.

### 2.3 Ferroptosis Is Induced by *RLIM* Depletion in OPCs

**FIGURE 4:**
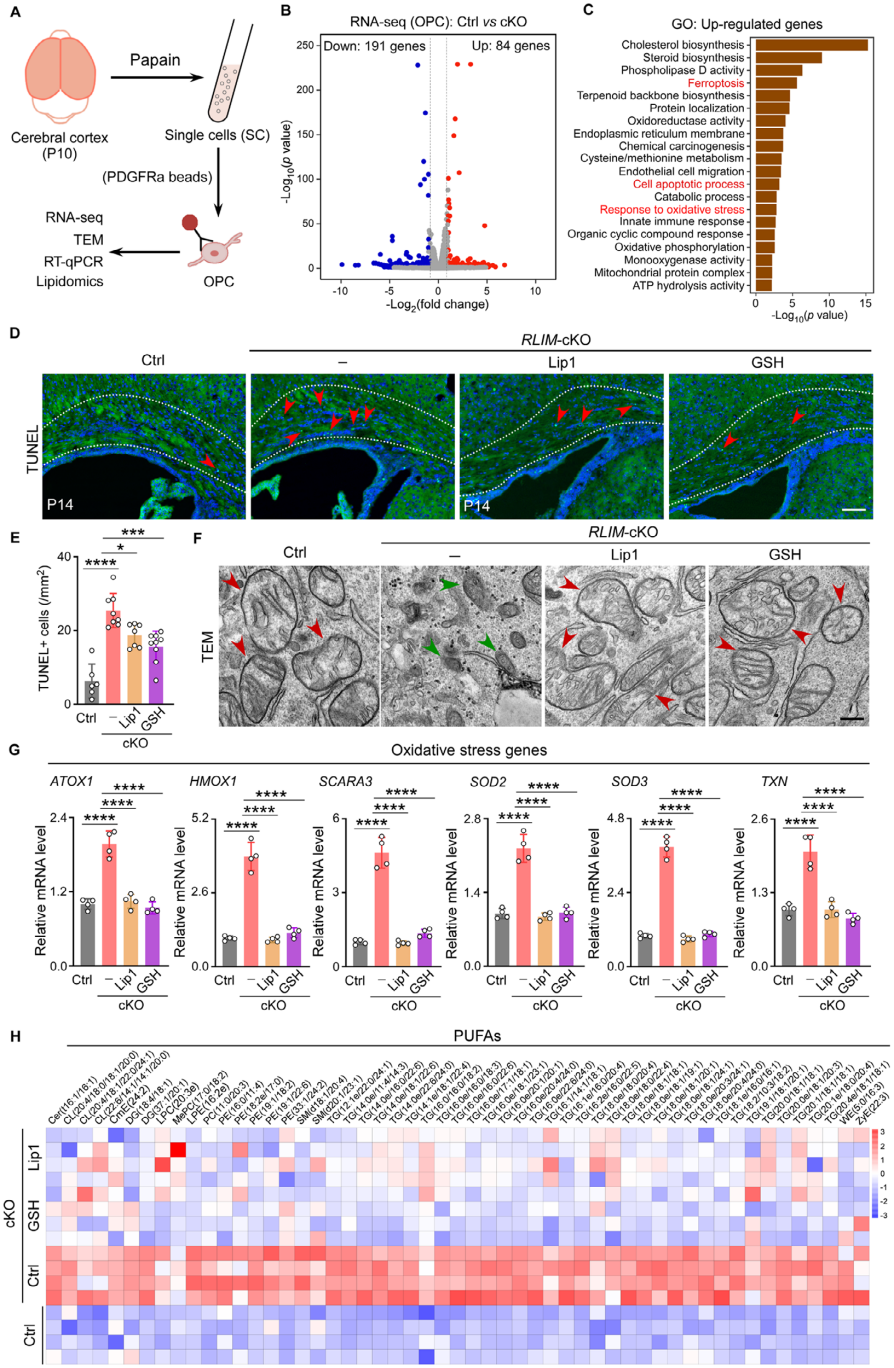
RLIM deficiency leads to ferroptosis in OPCs. (A) Schematic diagram of isolating PDGFRa^+^ OPC and the following analysis. (B) Volcano plot of RNA‐seq data shows differentially expressed genes (DEG) from isolated OPC cells in cortical tissues of P10 control (*n* = 3) and the *RLIM* cKO (*n* = 3) mice. (C) Representative Gene Ontology (GO) terms of the biological process categories enriched in the upregulated genes identified by RNA‐seq in (B). (D) Representative images of TUNEL staining showing cell death in corpus callosum tissues of the indicated mice (*n* = 3 for each group). Scale bar, 100 µm. (E) The bar graph shows the quantification of TUNEL^+^ cells in corpus callosum tissues in (D) (control: 6.284 ± 4.644 /mm^2^; *RLIM* cKO: 25.45 ± 4.631 /mm^2^; *RLIM* cKO + Lip1: 18.73 ± 3.172 /mm^2^; *RLIM* cKO + GSH: 15.64 ± 4.202 /mm^2^). One‐way ANOVA with multiple comparisons; ^*^, *p* < 0.05; ^**^, *p* < 0.01; ^****^, *p* < 0.0001. (F) OPC cells isolated from the indicated mice were subjected to transmission electron microscopy. Red arrows indicated mitochondria with obvious cristae, while green arrows represent shrunken mitochondria. Scale bar, 280 nm. (G) Realtime PCR results showing the relative expression level of the oxidative stress genes (*ATOX1*: control: 1.000 ± 0.08423, *RLIM* cKO: 1.972 ± 0.2112, *RLIM* cKO‐Lip1: 1.045 ± 0.1132, *RLIM* cKO‐GSH: 0.9394 ± 0.09871; *HMOX1*: control: 1.000 ± 0.03818, *RLIM* cKO: 3.885 ± 0.2484, *RLIM* cKO‐Lip1: 0.9484 ± 0.04610, *RLIM* cKO‐GSH: 1.193 ± 0.08757; *SCARA3*: control: 1.000 ± 0.1067, *RLIM* cKO: 4.622 ± 0.6266, *RLIM* cKO‐Lip1: 0.9560 ± 0.08838, *RLIM* cKO‐GSH: 1.360 ± 0.1625; *SOD2*: control: 1.000 ± 0.09531, *RLIM* cKO: 2.238 ± 0.2652, *RLIM* cKO‐Lip1: 0.9419 ± 0.06972, *RLIM* cKO‐GSH: 1.016 ± 0.1051; *SOD3*: control: 1.000 ± 0.07454, *RLIM* cKO: 3.883 ± 0.3282, *RLIM* cKO‐Lip1: 0.8964 ± 0.09686, *RLIM* cKO‐GSH: 1.058 ± 0.08440; *TXN*: control: 1.000 ± 0.1045, *RLIM* cKO: 2.023 ± 0.2975, *RLIM* cKO‐Lip1: 1.002 ± 0.1398, *RLIM* cKO‐GSH: 0.8450 ± 0.09066) in OPCs isolated from cortical tissues of the indiacated mice (*n* = 3 for each group). One‐way ANOVA with multiple comparisons; ^****^, *p* < 0.0001. (H) The heatmap shows the relative levels of upregulated lipid metabolites in *RLIM*‐deficient OPC cells in the indicated groups (*n* = 4 for each group). PE, phosphatidylethanolamine; PC, phosphatidylcholine; TG, triacylglycerol. Cer, ceramide. Ctrl, Control; cKO, *RLIM* cKO.

To uncover how RLIM regulates OL development, RNA sequencing (RNA‐seq) analysis of *RLIM*‐deficient OPCs purified from P10 cortical tissues identified 84 up‐regulated genes compared to cells from control mice, with pathways related to ferroptosis and apoptosis processes significantly enriched, as revealed by Gene Ontology (GO) analysis (Figure 4A–C; Table S1). Ferroptosis is a cell death form caused by iron overload and abnormal cellular lipid metabolism [25], and consistently, the GO analysis also revealed that genes related to lipid metabolism were dramatically changed in *RLIM*‐depleted OPCs (Figure 4C). To examine the effect of *RLIM* deficiency on survival of OPCs, siRNAs against *RLIM* were transfected into cultured MOPC cells, a mouse OPC cell line [60], and PI and TUNEL staining analyses were carried out. Both PI and TUNEL staining analyses showed that *RLIM* depletion induced cell death in MOPC cells (Figure S4A–D). In addition, lipid ROS, a biomarker of ferroptosis, accumulated in *RLIM*‐depleted MOPC cells (Figure S4E,F). Transmission electron microscopy analysis revealed that *RLIM*‐depleted MOPC cells contained shrunken mitochondria, which is a morphological feature of ferroptosis (Figure S4G). Further, untargeted lipidomic analysis indicates an increase in the amount of polyunsaturated fatty acids (PUFAs) (Figure S5 and Table S2), which is consistent with the current consensus that the oxidation of PUFA‐containing phospholipids drives ferroptosis [64]. To rule out the off‐target effects of *RLIM* siRNAs, we re‐expressed wild‐type or ubiquitin E3 ligase defective mutants in *RLIM* knockdown cells. And we found that restoration of wild‐type RLIM, but not its ligase‐dead mutant, rescued the phenotypes, including the cell death and lipid ROS, induced by *RLIM* deficiency in MOPC cells (Figures S5 and S6 and Table S2), suggesting that the E3 ligase activity is necessary for RLIM's function in regulating ferroptosis in OPC cells. To further confirm that ferroptosis was the major cell death form in *RLIM* depletion‐induced cell death, the *RLIM*‐deficient MOPC cells were treated with Liproxstatin‐1 (Lip1), a specific ferroptosis inhibitor [65], and the results showed that Lip1 treatment fully rescued the observed phenotypes in *RLIM*‐deficient MOPC cells (Figures S5 and S7 and Table S2).

Consistent with the in vitro results, in vivo TUNEL staining on corpus callosum sections revealed a significant accumulation of apoptotic cells in the *RLIM* cKO mice, compared to controls (Figure 4D,E). TEM analysis revealed that OPC cells from *RLIM* cKO mice also contained shrunken mitochondria (Figure 4F). Oxidative stress marker genes, including *ATOX1*, *HMOX1*, *SCARA3*, *SOD2*, *SOD3*, and *TXN*, were induced in OPC cells with *RLIM* knockout, indicating high oxidative stress (Figure 4G) [66]. An increase in the amount of PUFAs in OPC cells isolated from P10 cortical tissues of the *RLIM* cKO mice was also observed in lipidomic analysis (Figure 4H). Importantly, Lip1 supplement from P3 to P10 reversed the cell death, lipid ROS, and proliferation defects in OPCs from P14 brains of the *RLIM* cKO mice (Figure 4D–H; Figure S8A–C). Furthermore, protein levels of canonical ferroptosis markers, such as GPX4, ACSL4, and CHAC1, changed accordingly, while no activation of Caspase or PAPR was observed in *RLIM* cKO mice (Figure S8D–I). Taken together, these results suggest that RLIM is required for ferroptotic resistance and that *RLIM* deficiency induces ferroptosis in OPCs.

### 2.4 *RLIM* Catalyzes SLC7A11 for K63‐Linked Polyubiquitination, Which Is Required for Its Membrane Targeting and Function

To gain insights into how *RLIM* regulates ferroptosis in OPCs, TurboID‐based proximity labeling combined with mass spectrometry (MS) was employed to identify the potential targets involved in RLIM‐mediated ferroptosis regulation using N2A cells, a mouse neuroblast cell line, as previously reported [67, 68]. Principal component analysis (PCA) of three biological replicates revealed clear segregation between RLIM‐TurboID and TurboID‐only samples (Figure S9A). Proteins present in at least two replicates were retained, and statistical cut‐offs (*p*‐value < 0.01 and fold change ≥ 2) were applied to define the potential RLIM proximal interactome. This approach yielded 281 proteins that were uniquely present in RLIM‐TurboID samples, and we consider them the most potential candidates for RLIM direct binding and targeting (Figure S9B; Table S3). Among them, 51 proteins were also identified as candidates for RLIM interacting in mouse embryonic stem cells (mESCs) by the same strategy (Table S3) [69], confirming both the specificity and reliability of these results. Gene ontology (GO) analyses revealed strong enrichment for metabolism pathways (Figure S9C). Protein‐protein interaction networks were constructed using the Search Tool for Recurring Instances of Neighboring Genes (STRING, v12.0), and the resulting interactome was clustered into five functional modules, including aerobic respiration, amino and fatty acids metabolism, actin dynamics, translation, and collagen biogenesis (Figure S9D). Notably, SLC7A11, the cystine‐glutamate exchanger, whose expression has been reported to be closely related to ferroptosis regulation [27], was included in the RLIM proximal proteins, which prompted us to investigate the mechanistic connection between RLIM and SLC7A11.

**FIGURE 5:**
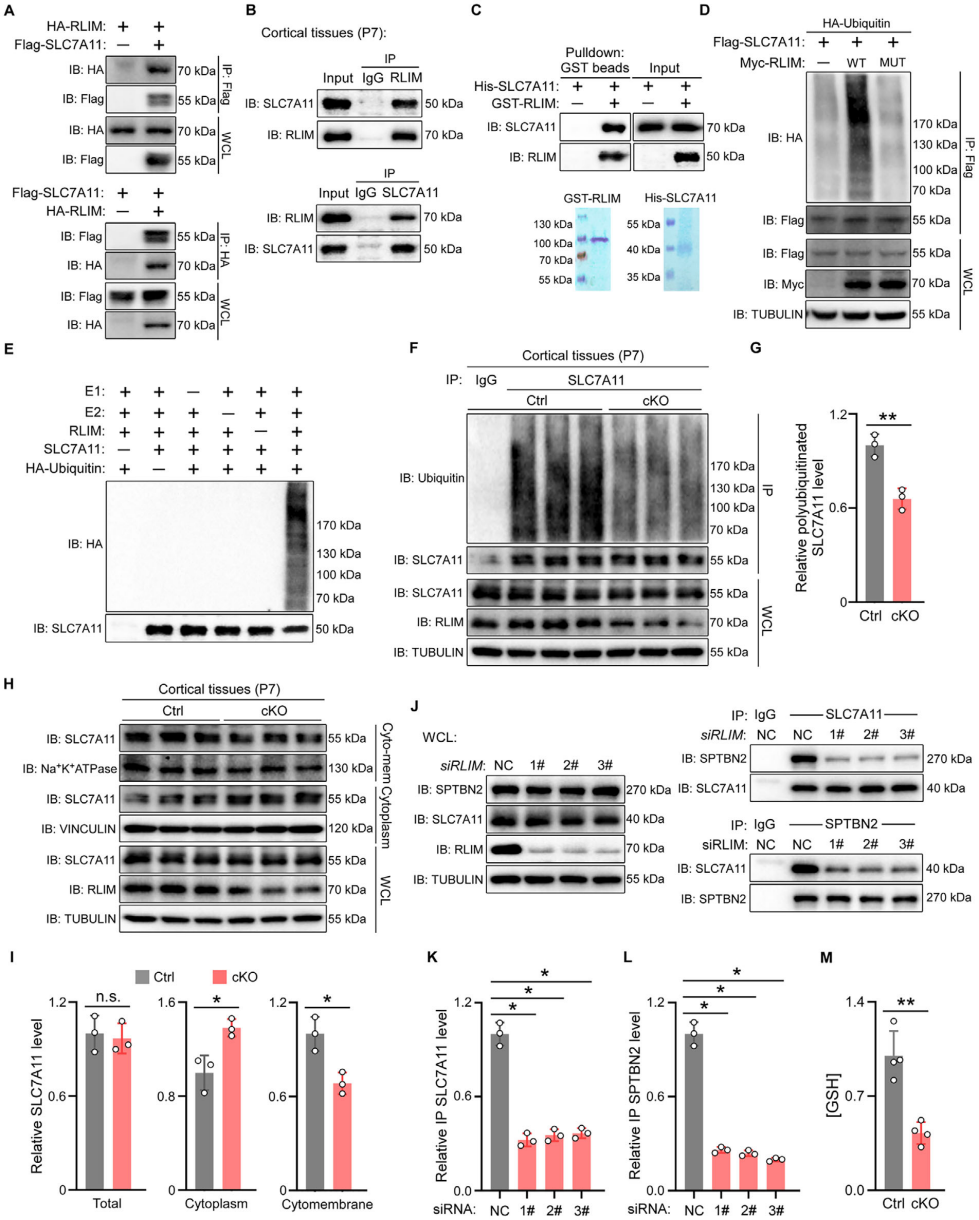
RLIM targets SLC7A11 for polyubiquitination, by which RLIM facilitates the membrane expression and function of SLC7A11. (A) In vitro co‐immunoprecipitation (co‐IP) analysis of the interaction between HA‐tagged RLIM and Flag‐tagged SLC7A11 in HEK293 cells with transfection as indicated. The whole cell lysates (WCLs) were immunoprecipitated by a Flag (the upper panel) or HA antibody (the lower panel), and the WCLs and immunoprecipitations (IPs) were immunoblotted with the indicated antibodies. (B) In vivo co‐IP analysis of the interaction between endogenous RLIM and SLC7A11 in cortical tissues from P7 control mice. The lysates were immunoprecipitated by an IgG and an RLIM (the upper panel) / SLC7A11 (the lower panel) antibody, and the inputs and IPs were immunoblotted by the indicated antibodies. (C) GST‐pulldown analysis of the direct interaction between RLIM and SLC7A11. Purified GST‐RLIM was incubated with purified His‐SLC7A11 and subjected to GST pulldown followed by immunoblotting with the indicated antibodies (the upper panel). Purified GST‐RLIM and His‐SLC7A11 proteins were analyzed by SDS‐PAGE and Coomassie blue staining (the lower panel). (D) Polyubiquitination analysis of SLC7A11 by co‐expression of wild‐type or the E3 ligase dead mutant (H569/572A) of RLIM in HEK293 cells. (E) In vitro ubiquitination assays of SLC7A11 by RLIM. In vitro purified SLC7A11 proteins were incubated in the presence of purified RLIM, ubiquitin‐activating enzyme E1, ubiquitin‐conjugating enzyme E2, and ubiquitin for the in vitro ubiquitination assay. (F, G) Analysis of ubiquitinated levels of SLC7A11 in cortical tissues of P7 control (*n* = 3) and the *RLIM* cKO (*n* = 3) mice. The bar graph (mean ± SD) shows normalized polyubiquitinated levels of SLC7A11 in Control and the *RLIM* cKO groups (control: 1.000 ± 0.07162, *RLIM* cKO: 0.6587 ± 0.06643). The respective expression level in the control group was set to 1. Unpaired two‐tailed Student's *t*‐test; ^**^, *p* < 0.01. (H, I) Results of Western blot assays showing the subcellular distribution of endogenous SLC7A11 in the cortical tissues from P7 control (*n* = 3) and the *RLIM* cKO (*n* = 3) mice. Bar graphs (mean ± SD) (I) show the normalized protein levels of SLC7A11 in total cell lysates (the left panel) (control: 1.000 ± 0.1148, *RLIM* cKO: 0.9686 ± 0.09590), cytoplasmic fractions (the middle panel) (control: 1.000 ± 0.1489, *RLIM* cKO: 1.382 ± 0.07420), and cyto‐membrane fractions (the right panel) (control: 1.000 ± 0.1080, *RLIM* cKO: 0.6850 ± 0.06910). The protein levels of SLC7A11 in total cell lysates, cytoplasmic fractions, or cyto‐membrane fractions were normalized against the levels of Na^+^K^+^ATPase, VINCULIN, or TUBULIN, respectively. The protein levels of SLC7A11 in the control groups were set to 1. Unpaired two‐tailed Student's *t*‐test; n.s., not significant; ^*^; *p* < 0.05. (J‐L) In vivo co‐IP analysis of the interaction between endogenous SPTBN2 and SLC7A11 in MOPC cells. The lysates were immunoprecipitated by an IgG and an SLC7A11 (the right upper panel) / SPTBN2 (the right lower panel) antibody, and the inputs and IPs were immunoblotted by the indicated antibodies. Bar graphs (mean ± SD) (K) shows the normalized protein levels of immunoprecipitated SLC7A11 by SPTBN2 (K) (NC: 1.000 ± 0.07347, siRLIM‐1#: 0.3243 ± 0.04121, siRLIM‐2#: 0.3546 ± 0.03816, siRLIM‐3#: 0.3672 ± 0.03259) or SPTBN2 by SLC7A11 (L) (NC: 1.000 ± 0.07482, siRLIM‐1#: 0.2600 ± 0.01888, siRLIM‐2#: 0.2387 ± 0.01864, siRLIM‐3#: 0.1988 ± 0.01146). The protein level of immunoprecipitated SLC7A11 or SPTBN2 in the NC group was set to 1. One‐way ANOVA with multiple comparisons; ^*^, *p* < 0.05 (M). The bar graph shows intracellular GSH levels in the PDGFRα^+^ OPC cells from cortical tissues of P7 Control (*n* = 4) and the *RLIM* cKO (*n* = 4) mice (control: 1.000 ± 0.1840, *RLIM* cKO: 0.4259 ± 0.08240). The GSH level in the control group was set to 1. Unpaired two‐tailed Student's *t*‐test; ^**^, *p* < 0.01. NC, negative control; Ctrl, control; cKO, *RLIM* cKO; IP, immunoprecipitation; WCL, whole cell lysate; IB, immunoblotting.

First, we confirmed the interaction between RLIM and SLC7A11 by using co‐immunoprecipitation (co‐IP) assays. In HEK293 cells transiently cotransfected with FLAG‐tagged SLC7A11 and HA‐tagged RLIM, SLC7A11 coimmunoprecipitated with RLIM. In the reverse experiment, RLIM also coimmunoprecipitated with SLC7A11 (Figure 5A). The interaction between endogenous RLIM and SLC7A11 proteins was then tested by co‐IP assays in MOPC cells or mouse cortical tissues. The results confirmed that the endogenous RLIM could be detected in SLC7A11 immunoprecipitates and that SLC7A11 was present in RLIM immunoprecipitates (Figure 5B; Figure S10A). Moreover, purified GST‐RLIM bound to His‐SLC7A11 in a cell‐free system, demonstrating a direct interaction between RLIM and SLC7A11 (Figure 5C).

Given that RLIM is a RING‐type E3 ubiquitin ligase that often regulates the stability of its target proteins via direct ubiquitination [58, 70, 71, 72, 73], we carried out ubiquitination assays to examine whether RLIM may affect the ubiquitination status of SLC7A11 and found that co‐expression of RLIM wild‐type protein, but not its ligase‐dead mutant, promotes the ubiquitination of SLC7A11 (Figure 5D). To further determine whether RLIM directly ubiquitinates SLC7A11 as an E3 ligase, in vitro ubiquitination assays were carried out by using purified proteins in a cell‐free system, and the result showed that RLIM markedly induced SLC7A11 polyubiquitination in vitro, suggesting that SLC7A11 is a direct substrate of RLIM (Figure 5E). Moreover, the ubiquitination level of endogenous SLC7A11 was markedly decreased in MOPC cells with *RLIM* knockdown by the transfection of its three independent siRNAs (Figure S10B,C). Further, when the endogenous SLC7A11 protein was immunoprecipitated from cortical tissues of P7 mice, their polyubiquitination levels in the *RLIM* cKO mice were significantly downregulated compared to controls (Figure 5F,G). Different types of ubiquitin linkages have distinct regulatory effects on protein stability, distribution, or activity [74, 75]. To determine the type of ubiquitin chain added by RLIM on the SLC7A11 protein, different and specific ubiquitin mutants were used in the ubiquitination assays in HEK293 cells. It was shown that SLC7A11 was only ubiquitinated by RLIM when K63‐type ubiquitin was present (Figure S10D). Consistent with this, when the K63R‐mutated ubiquitin was co‐expressed, the RLIM‐induced polyubiquitination level of the SLC7A11 protein was fully diminished (Figure S10E). Together, these results indicate that RLIM targets SLC7A11 for K63‐linked polyubiquitination. To further determine the responsible ubiquitination site(s), we individually mutated all potential lysines, which are present in the N‐ and C‐terminal regions and absent in the twelve transmembrane regions, into arginines individually and then examined RLIM‐mediated ubiquitination of each mutant. We found that the lysine 475 (K475) in mouse SLC7A11 or its corresponding lysine 483 (K483) in human SLC7A11, which localizes in the C‐terminal regions, was responsible for the polyubiquitination of SLC7A11 by RLIM (Figure S10F–H). Together, these results indicate that RLIM regulates SLC7A11 ubiquitination by adding K63‐linked polyubiquitin chains at the amino acid site of K475 (mouse) or K483 (human) in the C‐terminal regions.

Unlike K48‐linked polyubiquitination, which always targets proteins for proteasomal degradation, K63‐linked polyubiquitination usually affects the interaction, trafficking, localization, and signaling transduction of target proteins [75, 76]. Next, we examined the effect of RLIM‐mediated polyubiquitination on SLC7A11. Unexpectedly, we found that the total protein level of SLC7A11 was not changed when RLIM was over‐expressed or depleted in our in vitro or in vivo experiments (Figure 5D; Figure S10B,C). We then quantitatively analyzed the membrane and cytoplasmic distribution of SLC7A11. When the different subcellular fractions were purified, and the SLC7A11 protein levels were analyzed by Western blots, a clear increase in distribution in the cytoplasm and a decrease in the cytomembrane fraction of SLC7A11 were observed when *RLIM* was depleted by siRNAs in MOPC cells or knocked out in cortical tissues of P7 mice (Figure 5H,I; Figure S11A–D). To answer how RLIM‐mediated polyubiquitination promotes membrane localization, we revisited our proteomic analysis of RLIM‐associated factors and found that SPTBN2, an SLC7A11 membrane trafficking and localization facilitating factor [77], was among the most highly enriched proteins (Figure S9B; Table S3). We then examined whether RLIM expression has an effect on the interaction between SPTBN2 and SLC7A11 using co‐IP analysis and found that expression of wild‐type RLIM, but not its ligase‐dead mutation, promotes the interaction between SPTBN2 and SLC7A11 (Figure S11E,F). To further confirm that RLIM‐mediated SLC7A11 polyubiquitination facilitates the interaction between SPTBN2 and SLC7A11, the interaction between SPTBN2 and wild‐type SLC7A11 or KR mutated SLC7A11 was examined in the presence of RLIM or not. The results showed that RLIM expression facilitates the interaction between SPTBN2 and wild‐type SLC7A11, but not the SLC7A11 KR mutants (Figure S11G,H). Endogenously, in *RLIM* knockdown MOPC cells, the interaction between SPTBN2 and SLC7A11 was largely weakened (Figure 5J–L). Collectively, these results suggest that RLIM catalyzes SLC7A11 for K63‐linked polyubiquitination at K475, which facilitates its binding to SPTBN2 and thus the trafficking and membrane localization.

Next, to test whether RLIM‐mediated SLC7A11 polyubiquitination is indeed involved in the regulation of ferroptosis in MOPC cells, we further examined the rescue effect of the expression of wild‐type or KR‐mutated SLC7A11 in *RLIM*‐deficient cells. The results showed that cell death and upregulated ROS and PUFAs levels in *RLIM*‐deficient cells were all fully reversed by wild‐type SLC7A11, but not its KR mutated form (Figure S5 and S12; Table S2), implying that the defects in SLC7A11 polyubiquitination caused by RLIM knockdown are responsible for ferroptosis in MOPC cells.

The patients harboring RLIM substitutional mutations, such as P77L, Y356C, and R611C, exhibit features of white matter abnormality and hypomyelination evidenced by MRI imaging examination [47, 49]. Therefore, we investigated the effect of all RLIM substitutional mutations identified in human patients on SLC7A11 regulation. Results of ubiquitination assays in HEK293 cells showed that all the tested missense variants showed markedly reduced ability to polyubiquitinate SLC7A11 (Figure S13A,B). In addition, when co‐expressed in HEK293 cells, most of the tested mutations impaired the interaction between RLIM and SLC7A11 (Figure S13C,D). These results indicate that the pathological mutations of RLIM are involved in regulating ubiquitination of SLC7A11, which might be a pathological mechanism for RLIM‐associated neurodevelopmental disorders.

### 2.5 GSH Supplement Restores the Defects of Oligodendrocyte Development, Myelination, and Cognitive Function in the *RLIM* cKO Mice

It is well established that SLC7A11, the core subunit of System Xc^−^, maintains cellular redox balance by importing cystine for glutathione synthesis, a key antioxidant preventing lipid peroxidation, thus reducing lipid peroxidation levels and protecting against ferroptosis. Supporting this, both cystine uptake and GSH synthesis, critical functions of membrane System Xc^−^, were diminished significantly following *RLIM* knockdown in MOPC cells (Figure S11I,J). Accordingly, GSH supplement rescued the cell death and upregulated ROS and PUFAs in *RLIM*‐deficient MOPC cells (Figures S5 and S7; Table S2). Furthermore, a decrease in the amount of GSH in OPCs isolated from cortical tissues of the *RLIM* cKO mice at P7 was observed compared to that from control mice (Figure 5M).

**FIGURE 6:**
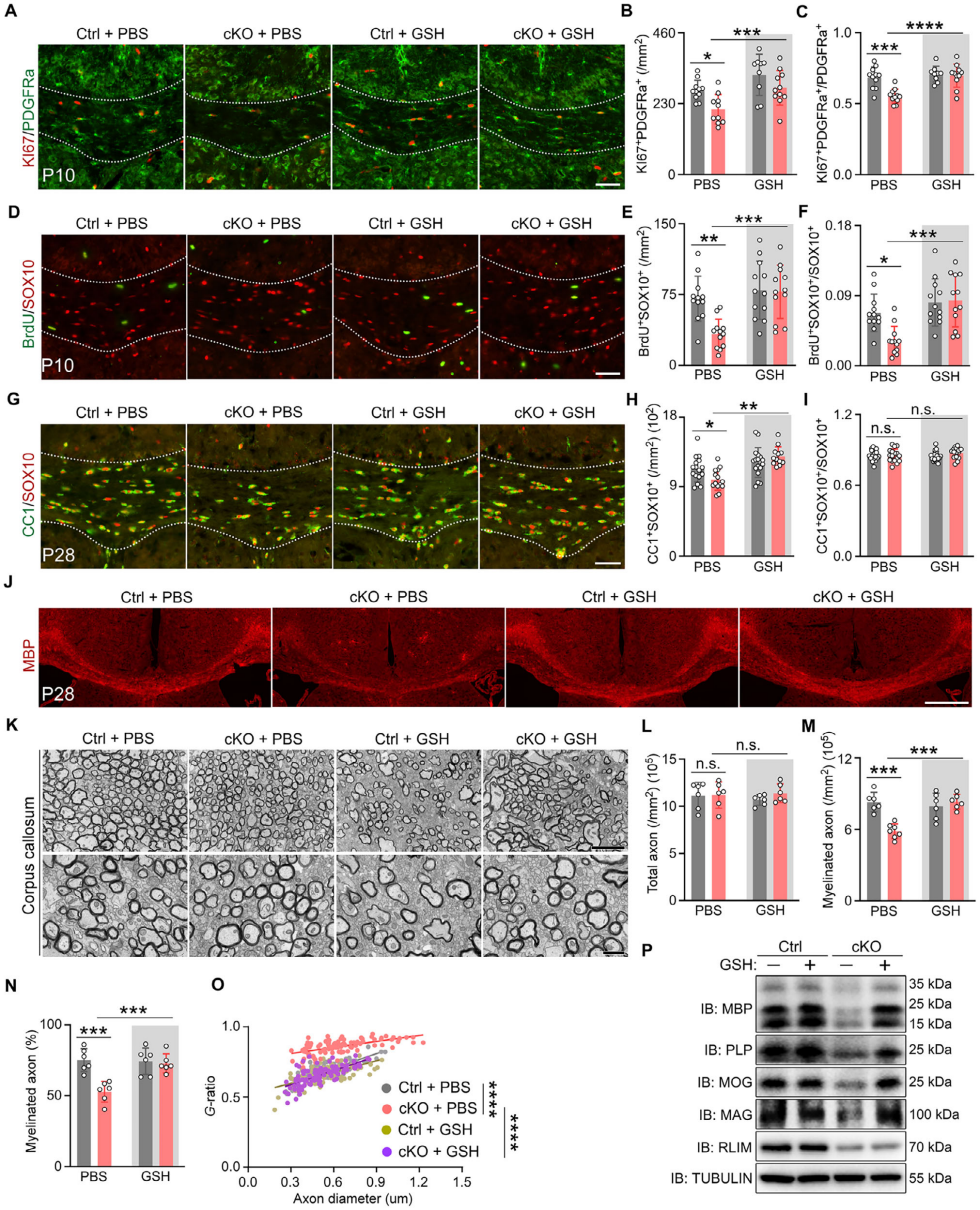
GSH administration restores defects in OL lineage development and myelination in the brains of the *RLIM* cKO mice. (A) Representative images of immunofluorescence staining assays showing KI67 and PDGFRα expression in the corpus callosum of control (*n* = 3) and the *RLIM* cKO (*n* = 3) mice at P10. Scale bar, 50 µm. (B) The bar graph shows the quantification of the number of KI67^+^PDGFRα^+^ cells in (A) (control‐PBS: 273.5 ± 32.79 /mm^2^, *RLIM* cKO‐PBS: 212.1 ±48.12 /mm^2^, control‐GSH: 323.5 ± 66.93 /mm^2^, and *RLIM* cKO‐GSH: 281.4 ± 56.11 /mm^2^). One‐way ANOVA with multiple comparisons; ^*^, *p* < 0.05; ^***^, *p* < 0.001. (C) The bar graph shows the quantification of the ratio of KI67^+^PDGFRα^+^ among PDGFRα^+^ cells in (A) (control‐PBS: 0.6842 ± 0.07540, *RLIM* cKO‐PBS: 0.5528 ±0.04978, control‐GSH: 0.7078 ± 0.05592, and *RLIM* cKO‐GSH: 0.6977 ± 0.08177). One‐way ANOVA with multiple comparisons; ^***^, *p* < 0.001; ^****^, *p* < 0.0001. (D) Representative images of immunofluorescence staining coupled with BrdU pulse labeling assays showing BrdU labeling and SOX10 expression in the corpus callosum of control (*n* = 3) and the *RLIM* cKO (*n* = 3) mice at P10. Scale bar, 50 µm. (E) The bar graph shows the quantification of the number of BrdU^+^SOX10^+^ cells in (D) (control‐PBS: 70.79 ± 23.75 /mm^2^, *RLIM* cKO‐PBS: 33.34 ± 15.61 /mm^2^, control‐GSH: 79.62 ± 30.87 /mm^2^, and *RLIM* cKO‐GSH: 78.03 ± 28.28 /mm^2^). One‐way ANOVA with multiple comparisons; ^**^, *p* < 0.01; ^***^, *p* < 0.001. (F) The bar graph shows the quantification of the ratio of BrdU^+^SOX10^+^ among SOX10^+^ cells in (D) (control‐PBS: 0.06798 ± 0.02408, *RLIM* cKO‐PBS: 0.03235 ± 0.01819, control‐GSH: 0.08121 ± 0.03021, and *RLIM* cKO‐GSH: 0.08381 ± 0.03392). One‐way ANOVA with multiple comparisons; ^*^, *p* < 0.05; ^***^, *p* < 0.001. (G) Representative images of immunofluorescence staining assays showing CC1 and SOX10 expression in the corpus callosum of control (*n* = 3) and the *RLIM* cKO (*n* = 3) mice at P21. Scale bar, 50 µm. (H) The bar graph shows the quantification of the number of CC1^+^SOX10^+^ cells in (G) (control‐PBS: 1127 ± 165.5 /mm^2^, *RLIM* cKO‐PBS: 991.4 ± 136.4 /mm^2^, control‐GSH: 1213 ± 192.6 /mm^2^, and *RLIM* cKO‐GSH: 1295 ± 122.6 /mm^2^). One‐way ANOVA with multiple comparisons; ^*^, *p* < 0.05; ^**^, *p* < 0.01. (I) The bar graph shows the quantification of the ratio of CC1^+^SOX10^+^ among SOX10^+^ cells in (K) (control‐PBS: 0.8555 ± 0.04350, *RLIM* cKO‐PBS: 0.8528 ± 0.05483, control‐GSH: 0.8457 ± 0.04009, and *RLIM* cKO‐GSH: 0.8749 ± 0.04635). One‐way ANOVA with multiple comparisons; n.s., not significant. (J) Representative images of immunofluorescence staining assays showing the expression of MBP in the corpus callosum of control‐PBS (*n* = 3), *RLIM* cKO‐PBS (*n* = 3), control‐GSH (*n* = 3), and *RLIM* cKO‐GSH (*n* = 3) mice at P28. Scale bar, 400 µm. (K) Transmission electron microscopy (TEM) images of the corpus callosum transverse sections from control‐PBS (n = 3), *RLIM* cKO‐PBS (*n* = 3), control‐GSH (*n* = 3), and *RLIM* cKO‐GSH (n = 3) mice at P60. Scale bars: 4 µm for the upper panels and 1 µm for the lower panels. (L) Bar graphs (mean ± SD) show quantification of the total axon number in (K) (control‐PBS: 1,112,500 ± 140,917 /mm^2^, *RLIM* cKO‐PBS: 1,119,167 ± 139,765 /mm^2^, control‐GSH: 1,066,667 ± 56,539 /mm^2^, and *RLIM* cKO‐GSH: 1,138,333 ± 99,079 /mm^2^). One‐way ANOVA with multiple comparisons; n.s., not significant. (M) Bar graphs (mean ± SD) show quantification of the number of myelinated axons in (K) (control‐PBS: 829,167 ± 81,450 /mm^2^, *RLIM* cKO‐PBS: 585,000 ± 60,896 /mm^2^, control‐GSH: 793,333 ± 110,393 /mm^2^, and *RLIM* cKO‐GSH: 831,667 ± 62,263 /mm^2^). One‐way ANOVA with multiple comparisons; ^***^, *p* < 0.001. (N) Bar graphs (mean ± SD) show quantification of the percentage of myelinated axons in (K) (control‐PBS: 75.13 ± 8.114%, *RLIM* cKO‐PBS: 52.80 ± 7.324%, control‐GSH: 74.35 ± 9.316%, and *RLIM* cKO‐GSH: 72.83 ± 6.765%). One‐way ANOVA with multiple comparisons; ^***^, *p* < 0.001. (O) Scatterplots show the myelin *g*‐ratios (diameter of axon/diameter of entire fiber). At least 200 axons from 3 mice were involved in the analysis for each group. General linear regression model and ANCOVA analysis; ^****^, *p* < 0.0001. (P) WB analyses of expression levels of the myelin‐related proteins, including MBP, PLP, MAG, and MOG, in the corpus callosum of control‐PBS (*n* = 3), *RLIM* cKO‐PBS (*n* = 3), control‐GSH (*n* = 3), and *RLIM* cKO‐GSH (*n* = 3) mice at P21. The statistics for (P) were included in the Supporting Information. Ctrl, Control; cKO, *RLIM* cKO; IB, immunoblotting.

The abnormal behaviors, including social and cognitive impairments, observed in the *RLIM* cKO mice recapitulate key aspects of RLIM‐affected neurodevelopmental disorders, providing a tractable model for mechanistic and therapeutic investigation. Given that *RLIM* loss leads to decreased membrane distribution and impaired function of SLC7A11, down‐regulated GSH synthesis, then reduced survival and ferroptosis in OPCs, and thus myelination defects, we wonder whether GSH supplement, which bypasses SLC7A11 function, could reverse OPC ferroptosis, myelination defects, and the associated behavioral abnormalities in the *RLIM* cKO mice, thereby functionally validating the downstream SLC7A11–GSH axis. To test this hypothesis, the *RLIM* cKO and control mice were first given a dosage of GSH supplements or vehicles from P3 to P10, and then the mice were harvested for OL development and myelination phenotyping and behavioral tests at the following indicated ages (Figure S14A). First of all, OPC cell ferroptosis was examined in the corpus callosum of mice with GSH supplement, and we found that cell death, shrunken mitochondria, upregulated PUFAs, and oxidative stress marker genes in OPC cells were all rescued by GSH supplement in *RLIM* cKO mice (Figure 4D–H). Then the OL developmental defects were checked. At P10, GSH supplements reversed the defect in OPC proliferation in the corpus callosum of the *RLIM* cKO mice, which is revealed by the BrdU pulse labeling and Ki67 co‐staining assays (Figure 6A–F). And also, CC1 and SOX10 co‐labeling analyses at P14 showed that the OPC differentiation defect was restored by GSH administration in the *RLIM* cKO mice (Figure 6G–I). Consistently, at P28, hypomyelination, indicated by MBP IF staining, examination of expression levels of myelin‐related genes by RT‐qPCR and Western blot, and transmission electron microscope analyses, was largely rescued by GSH supplementation in the *RLIM* cKO mice (Figure 6J–P; Figure S14B–K). Accordingly, at adult age, all the aberrant behaviors, including motor coordination defects, social and cognitive impairments, exhibited by the *RLIM* cKO mice, were significantly rectified to approach those of control mice (Figure S15). By contrast, GSH administration did not result in any discernible changes in OL development, myelination, and behavior in control mice (Figure 6; Figures S14 and S15).

### 2.6 *RLIM* Is Indispensable for Remyelination and GSH Administration Rescues Remyelination Defects in the *RLIM* icKO Adult Mice

**FIGURE 7:**
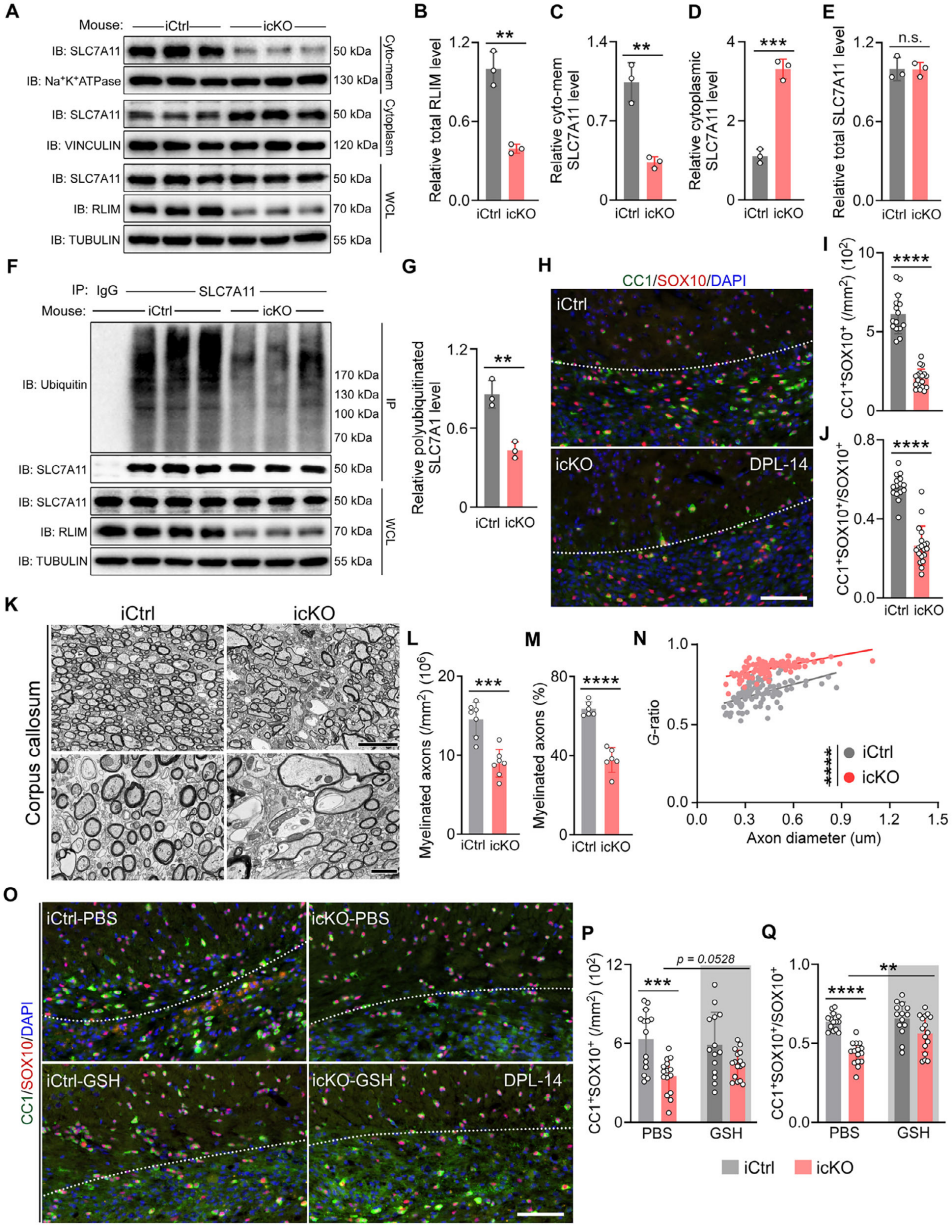
*RLIM* knockout in OL lineage cells impairs remyelination in adult mouse brains, and GSH administration restores the defect in remyelination in adult *RLIM* icKO mice. (A) Western blot assays showing the subcellular distribution of endogenous SLC7A11 in the cortical tissues from adult control (n = 3) and *RLIM* icKO (*n* = 3) mice with tamoxifen administration. (B‐E) Bar graphs (mean ± SD) (B) shows the normalized protein levels of RLIM in total cell lysates (B) (icontrol: 1.000 ± 0.1318 and *RLIM* icKO: 0.3930 ± 0.03400) or SLC7A11 in cytomembrane fractions (C) (icontrol: 1.000 ± 0.1719 and *RLIM* icKO: 0.3350 ± 0.04984), cytoplasmic fractions (D) (icontrol: 1.000 ± 0.1595 and *RLIM* icKO: 2.980 ± 0.2273), and total cell lysates (E) (icontrol: 1.000 ± 0.08803 and *RLIM* icKO: 0.9979 ± 0.05217). The protein levels of RLIM or SLC7A11 in total cell lysates, cytoplasmic fractions, or cytomembrane fractions were normalized against the levels of Na^+^K^+^ATPase, VINCULIN, or TUBULIN, respectively. The protein levels of RLIM or SLC7A11 in the control groups were set to 1. Unpaired two‐tailed Student's *t*‐test; n.s., not significant; ^**^, *p* < 0.01; ^***^, *p* < 0.001. (F, G) Analysis of ubiquitinated levels of SLC7A11 in cortical tissues of adult control (*n* = 3) and the *RLIM* icKO (*n* = 3) mice after tamoxifen administration. The bar graph (mean ± SD) shows normalized polyubiquitinated levels of SLC7A11 in icontrol and the *RLIM* icKO groups (icontrol: 1.000 ± 0.1220 and *RLIM* icKO: 0.5051 ± 0.07304). The respective expression level in the control group was set to 1. Unpaired two‐tailed Student's *t*‐test; ^**^, *p* < 0.01. (H‐J) Immunofluorescence staining of CC1 and SOX10 in lesion areas of brains from icontrol (*n* = 3) and the *RLIM* icKO (*n* = 3) at DPL 14. Bar graphs showing the quantification of the number of CC1^+^SOX10^+^ (I, icontrol: 611.8 ± 124.0 /mm^2^, and *RLIM* icKO: 202.6 ± 60.77 /mm^2^) and the ratio of CC1^+^SOX10^+^ cells among SOX10^+^ cells (J, icontrol: 0.5637 ± 0.06430, and *RLIM* icKO: 0.2656 ± 0.09807) in (H). Scale bar, 75 µm. Unpaired two‐tailed Student's *t*‐test; ^****^, *p* < 0.0001. (K–N) Electron microscopy images show the myelinated axons in the lesion area in the brains of icontrol (*n* = 3) and *RLIM* icKO (*n* = 3) at DPL 21. Scale bar: 4 µm for the upper panels and 1 µm for the lower panels. Bar graphs (mean ± SD) show quantification of the number of myelinated axons (L, icontrol: 1,456,450 ± 213,392 /mm^2^ and *RLIM* icKO: 898,144 ± 175,111 /mm^2^) and the percentage of myelinated axons (M, icontrol: 63.60 ± 3.527% and *RLIM* icKO: 37.78 ± 6.237%) in (K). Scatterplots (N) show the myelin *g*‐ratios (diameter of axon/diameter of entire fiber). At least 200 axons from 3 mice were involved in the analysis for each group. Unpaired two‐tailed Student's *t* test for (L) and (M); General linear regression model and ANCOVA analysis for (N); ^***^. *p* < 0.001; ^****^, *p* < 0.0001. (O–Q) Immunofluorescence staining of CC1 and SOX10 in lesion areas of brains from icontrol‐PBS (*n* = 3), *RLIM* icKO‐PBS (*n* = 3), icontrol‐GSH (*n* = 3), and *RLIM* icKO‐GSH (*n* = 3) at DPL 14. Bar graphs showing the quantification of the number of CC1^+^SOX10^+^ (P, icontrol‐PBS: 632.9 ± 225.7 /mm^2^, *RLIM* icKO‐PBS: 352.8 ± 133.3 /mm^2^, icontrol‐GSH: 588.9 ± 248.0 /mm^2^, *RLIM* icKO‐GSH: 440.3 ± 107.3 /mm^2^) and the ratio of CC1^+^SOX10^+^ cells among SOX10^+^ cells (Q, icontrol‐PBS: 0.6380 ± 0.05346, *RLIM* icKO‐PBS: 0.4370 ± 0.07134, icontrol‐GSH: 0.6567 ± 0.1049, *RLIM* icKO‐GSH: 0.5623 ± 0.1179) in (O). One‐way ANOVA with multiple comparisons; ^**^, *p* < 0.01; ^****^, *p* < 0.0001. iCtrl, icontrol; icKO, *RLIM* icKO.

Although the main signaling cascades and factors involved in the OL program of developmental myelination are shared by the remyelination program in adults [78], recent work has identified an OPC‐specific ubiquitin ligase that is only involved in remyelination [79]. We next investigated whether RLIM is required for OL regeneration and remyelination in adult mouse brains after demyelinating injury. The adult *RLIM* icKO mice were administered with tamoxifen for 7 days to fully induce the expected recombination, which was reflected by a specific and marked decrease in the protein level of RLIM in the adult OPCs of the *RLIM* icKO mice (Figure 7A,B). Consistently, we also confirmed that the ubiquitinated and membrane‐localized protein levels of SLC7A11 in OPCs of adult brains were significantly reduced when the *RLIM* icKO mice were treated with tamoxifen to induce recombination (Figure 7A–G). Then, the OL regeneration and remyelination after injury were examined in the *RLIM* icKO mice. One week after tamoxifen administration, LPC was injected into the corpus callosum for lesion induction, and the samples were harvested and examined on the day post lesion (DPL) 7, 14, and 21 (Figure S16A). The population of OL lineage cells at lesion sites was examined, and the results showed that although the number of SOX10^+^ OPCs at DPL 7 is comparable between the *RLIM* icKO and iControl mice (Figure S16B,C), a significant decrease in the number of SOX10^+^CC1^+^ OLs at DPL 14 was observed in the brains of the *RLIM* icKO mice (Figure 7H–J). These results indicate that *RLIM* depletion is detrimental to OL differentiation, but has no influence on OPC recruitment in the lesion sites during OL regeneration after injury in adult mouse brains. Consequently, it was shown that, at DPL 21, the number and percentage of remyelinated axons and the thickness of axonal myelin sheaths were severely reduced in the lesion sites of the corpus callosum of the *RLIM* icKO mice compared to controls, suggesting an impairment of the remyelination process (Figure 7K–N). Taken together, these data suggest that RLIM plays a crucial role in OL lineage cells regeneration in the context of white matter injury in mouse brains.

We further tested whether GSH administration could restore the RLIM deficiency‐induced OL regeneration and remyelination defects after injury in mouse brains. After 7 days of tamoxifen administration and the following LPC‐induced lesion, the control and *RLIM* icKO mice were resupplied with GSH supplements or vehicles for another 7 continuous days (Figure S16D). Then, the oligodendrocyte regeneration in lesion sites was assessed by IF staining assays. Similarly, the defect in OL regeneration, indicated by a decrease in the number of SOX10^+^CC1^+^ OLs in the lesion sites at DPL 14 in *RLIM* icKO, was reversed by GSH (Figure 7O–Q; Figure S16E,F), implying that GSH administration could also restore OL regeneration and myelination defects after injury in the *RLIM* icKO mice.

## 3 Discussion

This study not only uncovered an intricate cell survival checkpoint mechanism by which RLIM confers ferroptosis resistance to OPC to fight against lipid oxidation but also presented GSH administration as a promising strategy to restore *RLIM*‐affected neurodevelopmental disorders.

Although numerous studies focused on the pathological roles in tumor suppression and immunity, ferroptosis has also been recently reported to be implicated in the normal development and aging of many species [80, 81, 82]. One of the most critical axes for cell fate determination is how cells respond to oxidative stress, as most living organisms rely on oxygen as the ultimate electron acceptor in reductive/oxidative (redox)‐based metabolic processes [83]. Several pieces of evidence suggest that OL lineage OPCs are metabolically highly active cells in the CNS [13]. Our study elucidated a hitherto unknown protective mechanism by which OL lineage cells restrain ferroptosis, which extends a physiological function of ferroptotic resistance to OL development and myelination during normal neural development in mice.

GSH, the most abundant reductant in mammalian cells, is a cofactor for multiple enzymes responsible for ferroptotic resistance [28]. For GSH synthesis, cystine can be taken up from the environment by the system Xc^−^ cystine/glutamate antiporter, an SLC7A11‐containing transmembrane protein complex [27]. It has been demonstrated genetically that sustaining GSH synthesis or promoting the activity of system Xc^−^ can protect cells from death triggered by diverse oxidative stress conditions [84, 85, 86, 87]. As a major cystine transporter, SLC7A11 has been reported to be regulated by various biological processes. ATF3 and NRF2 induce SLC7A11 expression through transcriptional activation, while the transcriptional level of SLC7A11 can be repressed by p53 [29, 88, 89]. In addition to transcriptional modulation, the protein stability of SLC7A11 can be regulated by ubiquitination and deubiquitination [31, 33, 35]. In this study, we add a layer at the post‐translational level to SLC7A11 regulation, in which SLC7A11 membrane trafficking and function are facilitated by RLIM‐mediated non‐canonical polyubiquitination. Notably, GSH supplementation effectively restores OL development, myelination and remyelination, and reverses social and cognitive impairments in mice with *RLIM* depletion in OL lineage cells, underscoring the therapeutic potential of targeting ferroptosis in *RLIM*‐affected neurodevelopmental disorders.

It has been estimated that about 600 ubiquitin E3 ligase genes are present in the human genome, and mutations of more than 10% of them, including UBE3A, RNF220, and NEDD4L, have been reported to be associated with neurodevelopmental disorders [90, 91, 92, 93]. RLIM has been added to the list by recent studies showing that the patients harboring missense mutations, including Y44C, P77L, Y356C, R365C, Y387C, Y577H, R599C, and R611C, exhibit several brain abnormalities, such as intellectual disability, seizures, leukodystrophy, corpus callosum agenesis [47, 49]. These abnormalities are related to different brain regions and cell types, suggesting RLIM might regulate the respective biological processes through targeting various proteins. It is established that RLIM regulates cell fate determination in zebrafish early embryogenesis and mouse embryonic stem cells [71, 94, 95]. In this study, we found that, in OL lineage cells, RLIM is required for OL development and myelination, and thus social and cognitive performance, through regulating SLC7A11‐mediated ferroptotic resistance. Most of the pathological mutations of RLIM impair the ability to interact and/or ubiquitinate SLC7A11, suggesting deregulation of SLC7A11‐mediated ferroptotic resistance as the general pathological mechanism of RLIM‐associated neurodevelopmental disorders.

The insight garnered from our study of RLIM deficiency has profound implications beyond neurodevelopmental disorders. In addition to X chromosome inactivation, RLIM is indispensable for spermatogenesis in mice [57, 58, 96]. Aberrant expression of RLIM was also thought to be closely related to the progression of various cancers, including breast tumors, lung cancer, and medulloblastoma [72, 73, 97]. It is suggested that failure in ferroptotic resistance is a main driver for the progression of such diseases; therefore, overcoming it by GSH supplementation might be a powerful strategy for therapy. This notable postulation warrants further investigation, which will be instructive for developing therapies beneficial for affected patients.

While our study elucidates the critical role of the RLIM‐SLC7A11‐ferroptosis axis in oligodendrocyte development and myelination, and proposes GSH supplementation as a potential therapy for *RLIM*‐associated neurodevelopmental disorder, several limitations should be noted. First, while GSH administration rescued defects in mice, its efficacy in human models with *RLIM* mutations needs to be further tested. In addition, whether the observed rescue is specific to the SLC7A11–GSH axis or can be recapitulated by general antioxidants, such as vitamin E, remains to be determined. Furthermore, whether post‐developmental GSH administration could reverse established hypomyelination or behavioral deficits in adult *RLIM* cKO mice remains an open question and warrants further investigation. Second, although the key mechanistic findings were validated in primary OPCs, we acknowledge that our initial identification pipeline by TurboID proximity labeling in N2A cells does not fully recapitulate the oligodendrocyte lineage context; accordingly, oligodendrocyte lineage‐specific RLIM interactors/targets, which are involved in oligodendrocyte lineage progression, may remain undetected in the present screen, and their potential roles merit further investigation. Third, most RLIM mutations analyzed here were modeled by in vitro assays; generating knock‐in mice carrying the specific patient‐specific variant would better recapitulate human pathology. Addressing these limitations in future studies could strengthen the translational potential of targeting ferroptosis for neurodevelopmental disorders.

## 4 Methods and Materials

### 4.1 Animals

All mice with a C57BL/6 background were maintained and handled according to guidelines approved by the Animal Care and Use Committee of the Kunming Institute of Zoology, Chinese Academy of Sciences (IACUC‐PA‐2025‐03‐038). The RLIM conditional knockout mouse line *RLIM^flox/wt^* were obtained from GemPharmatech (strain no. T025874), the *Olig2‐Cre* and *PDGFRα‐CreER* mouse lines were gifts from Zengqiang Yuan's (Beijing Institute of Basic Medical Sciences) and Jianqin Niu's (Third Military Medical University) labs, respectively, and were used as previously described [60, 62, 98]. The genotypes were confirmed by PCR as previously described [99], using genomic DNA prepared from tail tips as a template and the following primers: 5’‐CGGCTGCTACTATATTTATTGCCTGG‐3’ (forward) and 5’‐GGATCAAATGAGACCATACCTCAGTG‐3’ (reverse) for the floxed and wild‐type *RLIM* alleles, and 5’‐GCCTGCATTACCGGTCGATGC‐3’ (forward) and 5’‐ CAGGGTGTTATAAGCAATCCC‐3’ (reverse) for the *Cre* allele.

To induce *RLIM* knockout in adult *RLIM^flox/flox^;PDGFRα‐CreER* mice, intragastric administration of tamoxifen (Sigma‐Aldrich, T5648) at a dose of 200 mg/kg body weight was given daily for 7 days continuously. The model of adult injury and remyelination was performed as described previously [100]. LPC (1 µL; Sigma‐Aldrich, L4219) of 1% solution was injected into the corpus callosum, at the coordinate: 0.8 mm lateral, 0.8 mm rostral to bregma, 1.2 mm deep to the brain surface, to induce a lesion 7 days after the tamoxifen‐administration. For BrdU pulse labeling, animals were injected intraperitoneally with BrdU (500 mg/kg; Sigma‐Aldrich, B5002) 2 h before sacrifice.

For in vivo rescue assays, the indicated mice were administered intraperitoneally with 10 mg/kg Liproxstatin‐1 or 0.5 g/kg GSH for a continuous seven days. Then the brains were harvested at the indicated timepoints.

### 4.2 MRI Data Acquisition

MRI data were obtained on a 7.0‐T Bruker MR system (BioSpec 70/20 USR, Bruker). The mice were anaesthetized by inhalation of 3% isoflurane (RWD, R510‐22‐10) before scanning, and physiological parameters were monitored and kept constant during the experiment. Tooth and ear bars were used to restrain the mice for imaging. A 2D rapid acquisition with relaxation enhancement sequence was applied to obtain T2‐weighted images with the following parameters: repetition time, 3500 ms; echo time, 45 ms; slice width, 0.6 mm.

### 4.3 Electron Microscopy

Mice were deeply anesthetized, perfused with cold PBS followed by 4% paraformaldehyde (PFA) (Sigma‐Aldrich, 158172). The corpus callosum tissues were dissected on ice and cut into small pieces. The indicated MOPC cells, isolated OPC cells, and pieces of corpus callosum were post‐fixed with 2.5% glutaraldehyde (Sigma‐Aldrich, G5882) overnight at 4°C; treated with 1% osmium tetroxide (Sigma‐Aldrich, 1.24505), ethanol (Sigma‐Aldrich, 459828), dehydrated, and acetone (Sigma‐Aldrich, 270725) transition; and embedded into epoxy resins (Structure Probe, SPI‐Pon 812). Ultrathin sections (60 nm) were stained with 2% uranyl acetate (Electron Microscopy Sciences, 22400–2) and lead citrate (Electron Microscopy Sciences, 22410) for electron microscopy imaging using JEM‐1400 plus (JEOL). The extent of axonal myelination was quantified by calculating the *g*‐ratio (the ratio between the inner and the outer diameter of the myelin sheath). ImageJ software (National Institute of Health) was used for measurements of the axonal caliber and axonal counting.

### 4.4 Immunofluorescence Analysis and Myelin Staining

Transverse brain sections of 12 µm thickness were prepared, and MOPC cells were seeded on LAB‐TEK chamber slides (ThermoFisher Scientific, 154453) for immunofluorescence assays as previously described [60, 99]. The following antibodies were used: anti‐RLIM (1:200; Sigma‐Aldrich, HPA0188895), anti‐RLIM (1:200; Abnova, H0051132‐A01), anti‐PDGFRα (1:400; R&D, AF1026), anti‐CC1 (1:200; Millipore, OP80), anti‐SOX10 (1:300; Oasis Biofarm, OB‐PGP001), anti‐MBP (1:200, Santa Cruz, sc‐271524), anti‐BrdU (1:200; Bio‐Rad, MCA6144), anti‐KI67 (1:200; Abcam, ab15580), and fluorescence‐conjugated secondary antibodies, Alexa Fluor 488/555/594 donkey/goat anti‐rabbit/mouse/rat/guinea pig immunoglobulin G (1:400; Invitrogen; A11076, A11055, A21202, A48269, A21206, A31572). Images were captured using a light microscope (Olympus, VS120) and analyzed with ImageJ software (National Institute of Health).

For myelination analysis, 20 µm brain coronal sections were prepared with a cryostat microtome (Leica, CM1850UV) and stained with the Black‐Gold II kit (Biosensis, TR‐100‐BG) according to the manufacturers’ instructions. Images were captured using a light microscope (Olympus, VS120) and analyzed with ImageJ software (National Institute of Health).

### 4.5 Cells, siRNAs, Plasmids, and Transfections

HEK293, MOPC, and N2A cells were used as previously described [60, 68, 101]. Generally, all the cells were grown in Dulbecco's Modified Eagle Medium (DMEM) (VivaCell Bioseiences, C3113‐0500) supplemented with 10% fetal bovine serum (FBS) (Gibico, 10099141C) and 100 mg/mL penicillin‐streptomycin (Biological Industries, 03‐031‐1B). All the cells were transfected using Lipofectamine 2000 (Invitrogen, 11668019) according to the manufacturer's instructions. The following small interfering RNAs (siRNAs) (RiboBio) were used to knockdown the expression of *RLIM* in MOPC cells: *siRLIM‐1#*: 5’‐GCAACAAACTTCGTAAACT‐3’, *siRLIM‐2#*: 5’‐GCCAAACTTCCGAGAATGA‐3’, and *siRLIM‐3#*: 5’‐GCAGAGAGATAGTATAGCT‐3’. Ubiquitin and RLIM expression plasmids were used as previously described [73, 102]. The coding region of mouse or human SLC7A11 was obtained from Origene (T233333) or SinoBiological (HG18882‐U) and sub‐cloned into the expression vector pCS2‐N‐Flag/His. Sequences encoding the CH domain of mouse SPTBN2 were synthesized by Tsingke and were cloned into the vector of PCMV‐Flag. To prepare the pathological mutation and KR mutation constructs of RLIM and SLC7A11, respectively, site‐directed mutagenesis was carried out by PCR‐driven overlap extension using *Pfu* DNA polymerase (TianGen, EP101‐02) as previously described [101].

Isolation of OL lineage cells was performed as described previously [59, 60]. In brief, mice at indicated developmental stages were deeply anesthetized, and then their forebrains were dissected and minced. Then, the tissues were incubated with PBS containing papain (Worthington, LS003119) and deoxyribonuclease I (Thermo Fisher Scientific, EN0521) for 30 min at 25°C. After digestion, the tissues were triturated using a Dounce homogenizer and then filtered through a 40 µm cell strainer. Single cell suspensions were incubated with anti‐ PDGFRα (Miltenyi, 130‐101‐502) or O4 (Miltenyi, 130‐096‐670) at 4°C for 4 h to 6 h. Cells bound to the beads were harvested for total RNA or protein extraction.

For rescue experiments in MOPC cells, plasmids encoding mouse RLIM and SLC7A11 wild‐type or their mutated proteins were transfected into the MOPC cells using a Lonza Nucleofector Kit 24 h after RLIM siRNAs were transfected. RLIM siRNA‐transfected MOPC cells were treated with 50 nm Liproxstatin‐1 (Lip1) (HY‐12726, MedChemExpress) and 50 mm GSH (HY‐D0187, MedChemExpress) for 48 h.

### 4.6 Ubiquitination, co‐IP, GST‐pulldown, and Western Blots Assays

Western blots (WB), in vitro and in vivo ubiquitination, and coimmunoprecipitation (co‐IP) assays were carried out as previously described [60, 101]. GST fusion protein expression and GST‐pulldown assays were carried out with the protocol as previously described [103]. Briefly, GST‐RLIM was expressed in BL21 (DE3) bacteria and purified using Glutathione Sepharose 4B (GE Healthcare). His‐SLC7A11 was expressed in HEK293 cells and purified by a His‐tag Protein Purification Kit (P2226, Beyotime). For GST pull‐down assay, GST‐RLIM fusion protein immobilized on Glutathione Sepharose beads was incubated with purified His‐SLC7A11 protein in 500 µL lysis buffer (50 mm Tris–HCl pH7.4, 150 mm NaCl, 0.5% NP 40 and protease inhibitor mixture) for 2 h at 4°C. The beads were washed five times with 500 µL lysis buffer, and immobilized proteins were eluted with SDS sample buffer and then detected by WB assays. MOPC cells or mouse cortical tissue were fractionated by Subcellular Protein Fractionation Kit for Cultured Cells (78840, ThermoFisher Scientific) or for Tissues (87790, ThermoFisher Scientific), respectively, according to the manufacturer's instructions as previously reported [99].

The following antibodies were used for immunoprecipitation or immunoblotting: anti‐HA (1:5000 for WB; Sigma‐Aldrich, H3663); anti‐Flag (1:5000 for WB and 5 µg for each IP; Sigma‐Aldrich, F7425), anti‐ubiquitin (1:1000 for WB and 10 µL for each IP; Santa Cruz Technology, sc‐8017), anti‐MBP (1:1000 for WB, SantaCruz, sc‐271524), anti‐PLP (1:1000 for WB, Cell Signaling Technology, 85971S), anti‐MOG (1:1000 for WB, Proteintech, 12690‐1‐AP), anti‐MAG (1:1000 for WB, Proteintech, 14386‐1‐AP), anti‐SLC7A11 (1:1000 for WB and 10 µL for each IP, Proteintech, 26864‐1‐AP), anti‐SPTBN2 (1:1000 for WB and 10 µL for each IP, Proteintech, 55107‐1‐AP), anti‐Na^+^K^+^ATPase (1:1000 for WB, Proteintech, 55187‐1‐AP), anti‐VINCULIN (1:1000 for WB, Proteintech, 26520‐1‐AP), anti‐TUBULIN (1:5000, Proteintech, 66031‐1‐Ig), and anti‐RLIM (1:1000 for WB and 10 µL for each IP, Sigma‐Aldrich, HPA018895). HRP‐coupled donkey anti‐mouse (1:5000; ThermoFisher Scientific, 31430) and anti‐rabbit (1:5000; ThermoFisher Scientific, 31460) secondary antibodies were used, and the following chemilluminescence detection was carried out using a kit (ThermoFisher Scientific, 34579) according to the manufacturer's instructions. Immunoreactive bands were captured by a molecular imager (ChemiDoc XRS^+^, BIO‐RAD) and then quantified using ImageJ software (National Institute of Health). All original, unaltered blot images of the main and supplementary figures were included in the supplementary information accompanying the manuscript.

### 4.7 Cystine Uptake and GSH Assay

Cystine uptake for MOPC cells was measured as previously described using the Cystine Uptake Assay Kit (Dojindo Molecular Technologies, UP05) according to the manufacturer's instructions [77]. Briefly, cells were seeded at a density of 3000 cells per well in a black 96‐well microplate. The assays were conducted when the cells were cultured until they reached approximately 80% confluence, ensuring optimal conditions for the assay. Then, the medium was replaced with a cystine‐free medium, and a cystine analog probe was introduced and incubated for 30 min at 37°C. After that, the cells were subsequently washed with cystine‐free medium. The cystine uptake capacity of the cells was quantified by measuring fluorescence at λ_ex_ = 490 nm and λ_em_ = 535 nm. The results were normalized to total protein using the BCA Protein Assay Kit (ThermoFisher Scientific, 23225).

The relative GSH concentration in cell lysates was detected as previously described using a reduced glutathione (GSH) test kit (BC1170, Solarbio) according to the manufacturer's instructions [77]. Cultured MOPC and isolated OPC cells were suspended and lysed in assay buffer. To ensure complete lysis, the cell suspension underwent 2–3 freeze‐thaw cycles (freezing in liquid nitrogen and thawing in a 37°C water bath). Then the lysates were centrifuged at 8,000 g for 10 min, and the supernatant was collected and placed on ice for subsequent analysis. The sample was then mixed with DTNB solution in a 96‐well microplate, and the reaction was initiated by adding NADPH solution. After a 20‐min incubation, the luminescence at 412 nm was measured by a microplate reader (BioTek).

### 4.8 TurboID‐Based Proximity Labeling Assay

TurboID‐based proximity labeling was performed in N2A cells as described previously [67, 68]. To prepare samples for mass spectrometry analysis, N2A cells were seeded in 10 cm culture dishes and transfected with plasmids encoding TurboID or RLIM‐TurboID. 48 hours after transfection, cells were treated with 500 mM biotin (Sigma‐Aldrich, B4501) for 15 min at 37°C. Biotin labeling was terminated by transferring the cells onto ice. Cells were washed three times with cold PBS, then collected and lysed with a RIPA lysis buffer (weak) (Beyotime, P0013D) supplemented with a protease inhibitor cocktail (Roche, 04693132001) on ice for 30 min. The lysates were clarified by centrifugation at 12 000 g for 10 min at 4°C. The biotinylated proteins were purified and analyzed as described previously [104]. Briefly, 100 µL streptavidin magnetic beads (ThermoFisher Scientific, 88817) were washed three times with RIPA buffer and then added to the lysate supernatant, and the mixture was incubated at 4°C with gentle rotation overnight. Then the supernatant was discarded and the beads were collected and washed with the following buffers: 1 mL RIPA lysis buffer (twice), 1 mL 1 m KCl (once), 1 mL 100 mm Na_2_CO_3_ buffer (once), 1 mL urea buffer [2 m urea in 10 mm Tris‐HCl (pH 8.0), once], and 1 mL RIPA lysis buffer (twice). Biotinylated proteins were eluted from the beads through boiling in a 2× SDS protein loading buffer (Beyotime, P0015L), and the supernatant was then collected and transferred to new tubes. Three technical replicates were carried out independently to purify the biotinylated proteins. The purified proteins were quantitatively analyzed by liquid chromatography‐tandem mass spectrometry.

### 4.9 Cell Death Detection and Lipid Peroxidation Measurements

For lipid ROS examination, MOPC cells cultured in 96‐well microplates were incubated with 10 mm BODIPY 581/591 C11 reagent (D3861, Invitrogen) for 30 min at 37°C. The cellular fluorescence was detected by a fluorescence microscope (IX73, OLYMPUS) or flow cytometry (LSRFortessa X‐20, BD) using 590 nm and 510 nm channels. Results of flow cytometry were analyzed by FlowJo v10 (BD).

For PI staining, MOPC cells were incubated with 100 ng/mL Propidium Iodide (PI, P4170, Sigma) for 30 min, and images were taken using a fluorescence microscope (IX73, OLYMPUS). At least 3 microscopic fields were captured and evaluated for each well. At least three wells were assigned to one group and served as an independent test. Cell death was calculated using ImageJ (National Institute of Health) as the percentage of PI^+^ cells.

### 4.10 BrdU Labeling and TUNEL Assays

For BrdU pulse labeling experiments to analyze cell proliferation in vivo, mice were given 1 injection of BrdU at 100 mg/kg body weight, and brains were removed for analysis 2 h later. Tissue sections were immersed in 0.01 M citrate buffer at 95°C for 5 min, 2 m HCl at 37°C for 20 min, 0.1 m sodium borate for 10 min, and then washed in PBS. Treated sections were immunostained with anti‐BrdU antibody as described previously [99]. TUNEL staining assays on tissue sections or cultured cells were performed using a TUNEL FITC Apoptosis Detection Kit (A111‐01, Vazyme). Images were captured using a fluorescence microscope (IX73, OLYMPUS) and quantified using the ImageJ software (National Institute of Health).

### 4.11 Reverse Transcription‐Quantitative PCR (RT‐qPCR) and RNA‐Sequencing (RNA‐seq) Analysis

The total RNAs were extracted with TRNzol reagent (DP424, TIANGEN) and cDNAs were obtained by performing reverse transcription with the RevertAid H Minus First Strand cDNA Synthesis Kit (K1632, ThermoScientific) according to the manufacturer's instructions. qPCR was performed with LightCycler480 SYBR Green I Master (Roche) on a LightCycler480 system (Roche). All reactions were run in replicates of at least 3. Primers were as follows: 5’‐GCCAACCGTGAAAAGATGAC‐3’ (forward) and 5’‐GAGGCATACAGGGACAGCAC‐3’ (reverse) for mouse *β‐ACTIN*, 5’‐GGTCCACCACCACAGAGC‐3’ (forward) and 5’‐TGACCACTTCTTGTTGTATTTCC‐3’ (reverse) for mouse *RLIM*, 5’‐GACCATCCAAGAAGACCCCAC‐3’ (forward) and 5’‐GCCATAATGGGTAGTTCTCGTGT‐3’ (reverse) for mouse *MBP*, 5’‐CCAGAATGTATGGTGTTCTCCC‐3’ (forward) and 5’‐GGCCCATGAGTTTAAGGACG‐3’ (reverse) for mouse *PLP*, 5’‐CTGCCGCTGTTTTGGATAATGA‐3’ (forward) and 5’‐CATCGGGGAAGTCGAAACGG‐3’ (reverse) for mouse *MAG*, 5’‐AGCTGCTTCCTCTCCCTTCTC‐3’ (forward) and 5’‐ACTAAAGCCCGGATGGGATAC‐3’ (reverse) for mouse *MOG*, 5’‐ATGCCGAAGCACGAGTTCTC‐3’ (forward) and 5’‐ATGCAGACCTTCTTGTTGGGC‐3’ (reverse) for mouse *ATOX1*, 5’‐AAGCCGAGAATGCTGAGTTCA‐3’ (forward) and 5’‐GCCGTGTAGATATGGTACAAGGA‐3’ (reverse) for mouse *HMOX1*, 5’‐TGACAGGGATGTACTGTGTGT‐3’ (forward) and 5’‐TGCAAAGATAGGTTCTTCTGGC‐3’ (reverse) for mouse *SCARA3*, 5’‐CAGACCTGCCTTACGACTATGG‐3’ (forward) and 5’‐CTCGGTGGCGTTGAGATTGTT‐3’ (reverse) for mouse *SOD2*, 5’‐CCTTCTTGTTCTACGGCTTGC‐3’ (forward) and 5’‐TCGCCTATCTTCTCAACCAGG‐3’ (reverse) for mouse *SOD3*, and 5’‐CATGCCGACCTTCCAGTTTTA‐3’ (forward) and 5’‐TTTCCTTGTTAGCACCGGAGA‐3’ (reverse) for mouse *TXN*.

RNA‐seq analysis was performed with the Illumina HiSeq platform to identify the differential expression gene in OPC cells purified from the *RLIM* cKO and control mice. Briefly, mRNAs were enriched with Oligo‐d(T) beads and reverse transcribed into cDNA. After the construction of the library, the libraries were sequenced by an Illumina HiSeq sequencer (PE‐150, USA). Fastq reads were trimmed and filtered, and then the clean reads were aligned to the reference genome, after which gene expression levels were quantified. The screened differentially expressed genes were analyzed by Gene Ontology (GO) function analysis (http://metascape.org) and Kyoto Encyclopedia of Genes and Genomes (KEGG) pathway function analysis.

### 4.12 Lipidomic Analysis

The indicated 10^6^ MOPC cells were collected in 60% methyl alcohol (diluted with water, containing 8.5 g/L ammonium bicarbonate). The suspensions were centrifuged to remove the supernatant. Cells were then frozen in liquid nitrogen and stored at ‐80°C. The subsequent operations were provided by Majorbio. Briefly, LC‐MS/MS analyses were performed using an UHPLC system (Vanquish, ThermoFisher Scientific), equipped with a Kinetex C18 column (2.1 × 100 mm, 1.7 µm, Phenomenex). The QE mass spectrometer was used for its ability to acquire MS/MS spectra on data‐dependent acquisition (DDA) mode in the control of the acquisition software (Lipidsearch, ThermoFisher Scientific, USA).

### 4.13 Mouse Behavioral Tests

All the neural behavioral experiments were approved by the Animal Care and Use Committee of the Kunming Institute of Zoology, Chinese Academy of Sciences. All behavioral experiments were performed in the light phase (9:00 a.m. to 6:00 p.m.) in a soundproof room with a neutral environment, and individual tests were performed at a relatively fixed time. 8‐week‐old mice were used for all tests. The mice were first given a 1‐hour habituation period after being transferred to the rooms for behavioral tests. The experimenter was blind to the group identity of the tested mice, and the inner surfaces of the instruments were cleaned with 75% alcohol after each test. Mice with different genotypes were tested in random order.

#### 4.13.1 Rotarod

Mice were tested for time or accelerating speed on the rotarod as previously described [105]. Three trials were administered in each test. Mice were allowed to have at least 10 min of rest between the trials. The best performance among the three trials was recorded for the performance of different genotypes.

#### 4.13.2 Open Field

Mice were placed individually in an open‐field arena housed within a sound‐attenuating cubicle and permitted to move freely. Trials lasted for 5 min. Animal motion and cumulative path length were recorded by the Activity Monitor software.

#### 4.13.3 New Object Recognition

The test consisted of three phases: habituation, training, and testing. In the habituation phase, the animals were allowed to explore an empty arena (a Plexiglas arena measuring 40 × 40 × 40 cm^3^) for 5 minutes. The training trial was performed 24 h later, and each mouse was individually placed into the arena containing two identical objects (A1 and A2), equidistant from each other, and allowed to explore the objects for 10 min. After 1 hour, during the test phase, one copy of the familiar object (A3) and a new object (B) were placed in the same location as during the training trial. The exploring time was recorded when the mouse touched the object with the tip of its nose or the front paws for a total of 5 min. The discrimination index was calculated as the difference in time exploring the novel and the familiar objects, expressed as the percentage ratio of the total time spent exploring both objects.

#### 4.13.4 Three‐Chamber Social Interaction

All strangers used were wild‐type C57BL/6 mice with matched age, body weight, and sex to the mice being tested. The social test apparatus was made of a clear glass box (60 × 40 × 30 cm^3^) with three equally divided chambers (20 × 40 × 30 cm^3^ each). The chambers were interconnected with openings (5 × 5 cm^2^) that could be opened or closed manually. The inverted cylindrical wire cups, which contain the strange mouse or an object (ball), were 10 cm in height and contained a 10 cm floor with the metal bars spaced 0.8 cm apart. The day before the test, each of the stranger mice was habituated inside the inverted wire cups, and each of the test mice was habituated to the apparatus with two empty wire cups inside the box for 15 min. On the test day, during the habituation phase, an empty wire cup was placed into the left and right chambers, the tested mouse was placed into the center chamber, and allowed to explore for 5 minutes, with all doors open between chambers. During the sociability test phase, an unfamiliar mouse (S1) was placed inside the inverted wire cup in one of the side chambers, a ball (B) was placed inside the inverted wire cup in another side chamber, and the test mouse was introduced to the center chamber with the doors to both side chambers closed. Then the doors between chambers were lifted simultaneously, and the test mouse was allowed to explore all three chambers for 10 minutes. During the social novelty test phase, the test mouse was placed in the central chamber with all doors closed between chambers. After a novel mouse (S2) was introduced into the inverted wired cup, replacing the ball (B) in one of the side chambers, the doors between chambers were lifted simultaneously, and the test mouse was allowed to explore all three chambers for an additional 10 minutes. Time spent in close proximity to the cup of the stranger mice (S1, S2) or ball (B) was analyzed.

#### 4.13.5 Morris Water Maze

The blue circular pool was 120 cm in diameter and was divided into four equal quadrants with two hypothetically crossed lines. The hidden circular platform located in the middle of the target quadrant was 10 cm in diameter and submerged 1 cm below the water surface. During the training period, mice were trained to find the hidden platform over 7 consecutive days with four trials each day using a semirandom set of start locations, with the restriction that one trial each day was from each of the four different starting positions. If a mouse failed to find the platform within 60 seconds, it was picked up and placed on the platform for another 20 seconds. On the 8^th^ day, 24 hours after the last training session, the platform was removed, and 60 s were given to each mouse to search for the platform in the pool, with the starting location opposite the previous position. The movement of the mice was monitored using Noldus software (EthoVision XT 8.0, Noldus Technology). Escape latency to find the platform, total distance moved, average velocity, total distance to platform, and duration in platform zone were automatically analyzed by the software.

### 4.14 Statistical Analysis

Data analysis was carried out in ImageJ (National Institute of Health), Excel (Microsoft), and GraphPad Prism (GraphPad Software), and data were presented by Mean ± SEM or Mean ± SD as indicated in figure legends. For biochemical analyses and immunostaining assays, all experiments were repeated at least three times independently, and statistics from at least three samples were analyzed by unpaired two‐tailed Student's *t*‐test or one‐way ANOVA with multiple comparisons as indicated in figure legends. For animal neural behaviors, differences between experimental and control mice were assessed by two‐tailed Student's *t*‐test or one‐way ANOVA with multiple comparisons as indicated in the figure legends.

## Author Contributions

P.M., S.Z and B.M. conceived and designed the project. P.M., Y.L., H.Z., J.Z., G.Y., and S.Y. conducted experiments and analyses. J.C. contributed the reagents. P.M. and Y.L. wrote the manuscript. Z.S., P.M., and B.M. revised the manuscript.

## Conflicts of Interest

The authors declare no conflict of interest.

## Supporting information




**Supporting File 1**: advs76315‐sup‐0001‐SuppMat.docx.

**Supporting File 2**: advs76315‐sup‐0002‐Data.zip.

**Supporting File 3**: advs76315‐sup‐0003‐TableS1.xlsx.

**Supporting File 4**: advs76315‐sup‐0004‐TableS2.xlsx.

**Supporting File 5**: advs76315‐sup‐0005‐TableS3.xlsx.

## Data Availability

The data that support the findings of this study are available from the corresponding author upon reasonable request.

## References

[1] C. Stadelmann , S. Timmler , A. Barrantes‐Freer , and M. Simons , “Myelin in the Central Nervous System: Structure, Function, and Pathology,” Physiological Reviews 99 (2019): 1381–1431, 10.1152/physrev.00031.2018. 31066630

[2] B. Zhou , Z. Zhu , B. R. Ransom , and X. Tong , “Oligodendrocyte Lineage Cells and Depression,” Molecular Psychiatry 26 (2020): 103–117, 10.1038/s41380-020-00930-0. 33144710 PMC7815509

[3] J. K. Knowles , A. Batra , H. Xu , and M. Monje , “Adaptive and Maladaptive Myelination in Health and Disease,” Nature Reviews Neurology 18 (2022): 735–746, 10.1038/s41582-022-00737-3. 36376595

[4] M. Simons , E. M. Gibson , and K. A. Nave , “Oligodendrocytes: Myelination, Plasticity, and Axonal Support,” Cold Spring Harbor Perspectives in Biology 16 (2024): a041359, 10.1101/cshperspect.a041359. 38621824 PMC11444305

[5] D. E. Bergles and W. D. Richardson , “Oligodendrocyte Development and Plasticity,” Cold Spring Harbor Perspectives in Biology 8 (2015): a020453, 10.1101/cshperspect.a020453. 26492571 PMC4743079

[6] B. Elbaz and B. Popko , “Molecular Control of Oligodendrocyte Development,” Trends in Neurosciences 42 (2019): 263–277, 10.1016/j.tins.2019.01.002. 30770136 PMC7397568

[7] R. J. M. Franklin and C. Ffrench‐Constant , “Regenerating CNS Myelin — From Mechanisms to Experimental Medicines,” Nature Reviews Neuroscience 18 (2017): 753–769, 10.1038/nrn.2017.136. 29142295

[8] C. Yi , A. Verkhratsky , and J. Niu , “Pathological Potential of Oligodendrocyte Precursor Cells: Terra Incognita,” Trends in Neurosciences 46 (2023): 581–596, 10.1016/j.tins.2023.04.003. 37183154

[9] B. Emery and T. L. Wood , “Regulators of Oligodendrocyte Differentiation,” Cold Spring Harbor Perspectives in Biology 16 (2024): a041358, 10.1101/cshperspect.a041358. 38503504 PMC11146316

[10] W. Xin and J. R. Chan , “Myelin Plasticity: Sculpting Circuits in Learning and Memory,” Nature Reviews Neuroscience 21 (2020): 682–694, 10.1038/s41583-020-00379-8. 33046886 PMC8018611

[11] O. de Faria, Jr. , H. Pivonkova , B. Varga , S. Timmler , K. A. Evans , and R. T. Káradóttir , “Periods of Synchronized Myelin Changes Shape Brain Function and Plasticity,” Nature Neuroscience 24 (2021): 1508–1521, 10.1038/s41593-021-00917-2. 34711959

[12] B. Yalçin , M. B. Pomrenze , K. Malacon , et al., “Myelin Plasticity in the Ventral Tegmental Area Is Required for Opioid Reward,” Nature 630 (2024): 677–685, 10.1038/s41586-024-07525-7. 38839962 PMC11186775

[13] J. Spaas , L. van Veggel , M. Schepers , et al., “Oxidative Stress and Impaired Oligodendrocyte Precursor Cell Differentiation in Neurological Disorders,” Cellular and Molecular Life Sciences 78 (2021): 4615–4637, 10.1007/s00018-021-03802-0. 33751149 PMC8195802

[14] W. Baron and D. Hoekstra , “On the Biogenesis of Myelin Membranes: Sorting, Trafficking and Cell Polarity,” FEBS Letters 584 (2009): 1760–1770, 10.1016/j.febslet.2009.10.085. 19896485

[15] S. Y. C. Chong , S. S. Rosenberg , S. P. J. Fancy , et al., “Neurite Outgrowth Inhibitor Nogo‐A Establishes Spatial Segregation and Extent of Oligodendrocyte Myelination,” Proceedings of the National Academy of Sciences 109 (2011): 1299–1304, 10.1073/pnas.1113540109. PMC3268264 22160722

[16] M. Bradl and H. Lassmann , “Oligodendrocytes: Biology and Pathology,” Acta Neuropathologica 119 (2009): 37–53, 10.1007/s00401-009-0601-5. 19847447 PMC2799635

[17] V. E. Miron , T. Kuhlmann , and J. P. Antel , “Cells of the Oligodendroglial Lineage, Myelination, and Remyelination,” Biochimica et Biophysica Acta (BBA)—Molecular Basis of Disease 1812 (2010): 184–193, 10.1016/j.bbadis.2010.09.010. 20887785

[18] M. Simons and K. A. Nave , “Oligodendrocytes: Myelination and Axonal Support,” Cold Spring Harbor Perspectives in Biology 8 (2015): a020479, 10.1101/cshperspect.a020479. 26101081 PMC4691794

[19] M. B. Rone , Q.‐L. Cui , J. Fang , et al., “Oligodendrogliopathy in Multiple Sclerosis: Low Glycolytic Metabolic Rate Promotes Oligodendrocyte Survival,” The Journal of Neuroscience 36 (2016): 4698–4707, 10.1523/JNEUROSCI.4077-15.2016. 27122029 PMC6601725

[20] C. Maffezzini , J. Calvo‐Garrido , A. Wredenberg , and C. Freyer , “Metabolic Regulation of Neurodifferentiation in the Adult Brain,” Cellular and Molecular Life Sciences 77 (2020): 2483–2496, 10.1007/s00018-019-03430-9. 31912194 PMC7320050

[21] N. Meyer and J. E. Rinholm , “Mitochondria in Myelinating Oligodendrocytes: Slow and out of Breath?,” Metabolites 11 (2021): 359, 10.3390/metabo11060359. 34198810 PMC8226700

[22] B. Valerio‐Gomes , D. M. Guimaraes , D. Szczupak , and R. Lent , “The Absolute Number of Oligodendrocytes in the Adult Mouse Brain,” Frontiers in Neuroanatomy 12 (2018): 90, 10.3389/fnana.2018.00090. 30425626 PMC6218541

[23] D. Tang , X. Chen , R. Kang , and G. Kroemer , “Ferroptosis: Molecular Mechanisms and Health Implications,” Cell Research 31 (2020): 107–125, 10.1038/s41422-020-00441-1. 33268902 PMC8026611

[24] X. Jiang , B. R. Stockwell , and M. Conrad , “Ferroptosis: Mechanisms, Biology and Role in Disease,” Nature Reviews Molecular Cell Biology 22 (2021): 266–282, 10.1038/s41580-020-00324-8. 33495651 PMC8142022

[25] D. Liang , A. M. Minikes , and X. Jiang , “Ferroptosis at the Intersection of Lipid Metabolism and Cellular Signaling,” Molecular Cell 82 (2022): 2215–2227, 10.1016/j.molcel.2022.03.022. 35390277 PMC9233073

[26] S. J. Dixon and J. A. Olzmann , “The Cell Biology of Ferroptosis,” Nature Reviews Molecular Cell Biology 25 (2024): 424–442, 10.1038/s41580-024-00703-5. 38366038 PMC12187608

[27] P. Koppula , L. Zhuang , and B. Gan , “Cystine Transporter SLC7A11/xCT in Cancer: Ferroptosis, Nutrient Dependency, and Cancer Therapy,” Protein & Cell 12 (2020): 599–620, 10.1007/s13238-020-00789-5. 33000412 PMC8310547

[28] X. Chen , J. Li , R. Kang , D. J. Klionsky , and D. Tang , “Ferroptosis: Machinery and Regulation,” Autophagy 17 (2020): 2054–2081, 10.1080/15548627.2020.1810918. 32804006 PMC8496712

[29] L. Jiang , N. Kon , T. Li , et al., “Ferroptosis as a p53‐mediated Activity during Tumour Suppression,” Nature 520 (2015): 57–62, 10.1038/nature14344. 25799988 PMC4455927

[30] Y. Zhang , J. Shi , X. Liu , et al., “BAP1 links Metabolic Regulation of Ferroptosis to Tumour Suppression,” Nature Cell Biology 20 (2018): 1181–1192, 10.1038/s41556-018-0178-0. 30202049 PMC6170713

[31] Q. Chen , W. Zheng , J. Guan , et al., “SOCS2‐enhanced Ubiquitination of SLC7A11 Promotes Ferroptosis and Radiosensitization in Hepatocellular Carcinoma,” Cell Death & Differentiation 30 (2022): 137–151, 10.1038/s41418-022-01051-7. 35995846 PMC9883449

[32] S.‐J. Chen , J. Zhang , T. Zhou , et al., “Epigenetically Upregulated NSUN2 Confers Ferroptosis Resistance in Endometrial Cancer via m5C Modification of SLC7A11 mRNA,” Redox Biology 69 (2023): 102975, 10.1016/j.redox.2023.102975. 38042059 PMC10711489

[33] Z. Wang , N. Shen , Z. Wang , et al., “TRIM3 facilitates Ferroptosis in Non‐small Cell Lung Cancer through Promoting SLC7A11/xCT K11‐linked Ubiquitination and Degradation,” Cell Death & Differentiation 31 (2023): 53–64, 10.1038/s41418-023-01239-5. 37978273 PMC10781973

[34] N. Wang , B. Shi , L. Ding , et al., “FMRP Protects Breast Cancer Cells from Ferroptosis by Promoting SLC7A11 Alternative Splicing through Interacting with hnRNPM,” Redox Biology 77 (2024): 103382, 10.1016/j.redox.2024.103382. 39388855 PMC11497378

[35] Q. Zhou , H. Yu , Y. Chen , J. Ren , Y. Lu , and Y. Sun , “The CRL3 KCTD10 Ubiquitin Ligase–USP18 Axis Coordinately Regulates Cystine Uptake and Ferroptosis by Modulating SLC7A11,” Proceedings of the National Academy of Sciences 121 (2024): 2320655121, 10.1073/pnas.2320655121. PMC11252818 38959043

[36] O. Pampliega , M. Domercq , F. N. Soria , P. Villoslada , A. Rodríguez‐Antigüedad , and C. Matute , “Increased Expression of Cystine/Glutamate Antiporter in Multiple Sclerosis,” Journal of Neuroinflammation 8 (2011): 63, 10.1186/1742-2094-8-63. 21639880 PMC3117706

[37] K. S. Evonuk , B. J. Baker , R. E. Doyle , et al., “Inhibition of System Xc− Transporter Attenuates Autoimmune Inflammatory Demyelination,” The Journal of Immunology 195 (2015): 450–463, 10.4049/jimmunol.1401108. 26071560 PMC4490999

[38] F. N. Soria , A. Zabala , O. Pampliega , et al., “Cystine/Glutamate Antiporter Blockage Induces Myelin Degeneration,” Glia 64 (2016): 1381–1395, 10.1002/glia.23011. 27247047

[39] S. Ottestad‐Hansen , Q. X. Hu , V. V. Follin‐Arbelet , et al., “The Cystine‐Glutamate Exchanger (xCT, Slc7a11) Is Expressed in Significant Concentrations in a Subpopulation of Astrocytes in the Mouse Brain,” Glia 66 (2018): 951–970, 10.1002/glia.23294. 29350434

[40] A. Nabeyama , A. Kurita , K. Asano , et al., “xCT Deficiency Accelerates Chemically Induced Tumorigenesis,” Proceedings of the National Academy of Sciences 107 (2010): 6436–6441, 10.1073/pnas.0912827107. PMC2852021 20308543

[41] G. Albertini , L. Deneyer , S. Ottestad‐Hansen , et al., “Genetic Deletion of X CT Attenuates Peripheral and Central Inflammation and Mitigates LPS ‐Induced Sickness and Depressive‐Like Behavior in Mice,” Glia 66 (2018): 1845–1861, 10.1002/glia.23343. 29693305

[42] D. Shen , W. Wu , J. Liu , et al., “Ferroptosis in Oligodendrocyte Progenitor Cells Mediates White Matter Injury after Hemorrhagic Stroke,” Cell Death & Disease 13 (2022): 259, 10.1038/s41419-022-04712-0. 35318305 PMC8941078

[43] V. Saverio , E. Ferrario , R. Monzani , et al., “AKRs Confer Oligodendrocytes Resistance to Differentiation‐stimulated Ferroptosis,” Redox Biology 79 (2024): 103463, 10.1016/j.redox.2024.103463. 39671850 PMC11699626

[44] D. Popovic , D. Vucic , and I. Dikic , “Ubiquitination in Disease Pathogenesis and Treatment,” Nature Medicine 20 (2014): 1242–1253, 10.1038/nm.3739. 25375928

[45] D. A. Cruz Walma , Z. Chen , A. N. Bullock , and K. M. Yamada , “Ubiquitin Ligases: Guardians of Mammalian Development,” Nature Reviews Molecular Cell Biology 23 (2022): 350–367, 10.1038/s41580-021-00448-5. 35079164

[46] C. Cai , Y. D. Tang , J. Zhai , and C. Zheng , “The RING Finger Protein family in Health and Disease,” Signal Transduction and Targeted Therapy 7 (2022): 300, 10.1038/s41392-022-01152-2. 36042206 PMC9424811

[47] H. Hu , S. A. Haas , J. Chelly , et al., “X‐exome Sequencing of 405 Unresolved Families Identifies Seven Novel Intellectual Disability Genes,” Molecular Psychiatry 21 (2015): 133–148, 10.1038/mp.2014.193. 25644381 PMC5414091

[48] E. Tønne , R. Holdhus , C. Stansberg , et al., “Syndromic X‐linked Intellectual Disability Segregating with a Missense Variant in RLIM,” European Journal of Human Genetics 23 (2015): 1652–1656, 10.1038/ejhg.2015.30. 25735484 PMC4795204

[49] S. G. M. Frints , A. Ozanturk , G. Rodríguez Criado , et al., “Pathogenic Variants in E3 Ubiquitin Ligase RLIM/RNF12 Lead to a Syndromic X‐linked Intellectual Disability and Behavior Disorder,” Molecular Psychiatry 24 (2019): 1748–1768, 10.1038/s41380-018-0065-x. 29728705

[50] E. E. Palmer , R. Carroll , M. Shaw , et al., “RLIM Is a Candidate Dosage‐Sensitive Gene for Individuals with Varying Duplications of Xq13, Intellectual Disability, and Distinct Facial Features,” The American Journal of Human Genetics 107 (2020): 1157–1169, 10.1016/j.ajhg.2020.10.005. 33159883 PMC7820564

[51] F. Bustos , C. Espejo‐Serrano , A. Segarra‐Fas , et al., “A Novel RLIM/RNF12 Variant Disrupts Protein Stability and Function to Cause Severe Tonne–Kalscheuer Syndrome,” Scientific Reports 11 (2021): 9560, 10.1038/s41598-021-88911-3. 33953269 PMC8100121

[52] S. Cuinat , C. Quélin , C. Effray , et al., “Extending the Clinical Spectrum of X‐linked Tonne‐Kalscheuer Syndrome (TOKAS): New Insights from the Fetal Perspective,” Journal of Medical Genetics 61 (2024): 824–832, 10.1136/jmg-2024-109854. 38849204 PMC11420740

[53] J. Shin , M. Bossenz , Y. Chung , et al., “Maternal Rnf12/RLIM Is Required for Imprinted X‐chromosome Inactivation in Mice,” Nature 467 (2010): 977–981, 10.1038/nature09457. 20962847 PMC2967734

[54] C. Gontan , E. M. Achame , J. Demmers , et al., “RNF12 initiates X‐chromosome Inactivation by Targeting REX1 for Degradation,” Nature 485 (2012): 386–390, 10.1038/nature11070. 22596162

[55] J. Shin , M. C. Wallingford , J. Gallant , et al., “RLIM Is Dispensable for X‐chromosome Inactivation in the Mouse Embryonic Epiblast,” Nature 511 (2014): 86–89, 10.1038/nature13286. 24870238 PMC4105192

[56] C. Gontan , H. Mira‐Bontenbal , A. Magaraki , et al., “REX1 is the Critical Target of RNF12 in Imprinted X Chromosome Inactivation in Mice,” Nature Communications 9 (2018): 4752, 10.1038/s41467-018-07060-w. PMC6232137 30420655

[57] F. Wang , M. G. Gervasi , A. Boskovic , et al., “Deficient Spermiogenesis in Mice Lacking Rlim,” Elife 10 (2021): 63556, 10.7554/eLife.63556. PMC7935487 33620316

[58] F. Wang , A. Chander , Y. Yoon , et al., “Roles of the Rlim–Rex1 Axis during X Chromosome Inactivation in Mice,” Proceedings of the National Academy of Sciences 120 (2023): 2313200120, 10.1073/pnas.2313200120. PMC10756295 38113263

[59] D. B. Swartzlander , N. E. Propson , E. R. Roy , et al., “Concurrent Cell Type–specific Isolation and Profiling of Mouse Brains in Inflammation and Alzheimer's Disease,” JCI Insight 3 (2018): 121109, 10.1172/jci.insight.121109. 29997299 PMC6124528

[60] Y. Li , L. P. Wan , N. N. Song , et al., “RNF220‐Mediated K63‐linked Polyubiquitination Stabilizes Olig Proteins during Oligodendroglial Development and Myelination,” Science Advances 10 (2024), 10.1126/sciadv.adk3931. PMC10849602 38324685

[61] U. Schüller , V. M. Heine , J. Mao , et al., “Acquisition of Granule Neuron Precursor Identity Is a Critical Determinant of Progenitor Cell Competence to Form Shh‐Induced Medulloblastoma,” Cancer Cell 14 (2008): 123–134, 10.1016/j.ccr.2008.07.005. 18691547 PMC2597270

[62] R. Wu , A. Li , B. Sun , et al., “A Novel m6A Reader Prrc2a Controls Oligodendroglial Specification and Myelination,” Cell Research 29 (2018): 23–41, 10.1038/s41422-018-0113-8. 30514900 PMC6318280

[63] S. H. Kang , M. Fukaya , J. K. Yang , J. D. Rothstein , and D. E. Bergles , “NG^2+^ CNS Glial Progenitors Remain Committed to the Oligodendrocyte Lineage in Postnatal Life and Following Neurodegeneration,” Neuron 68 (2010): 668–681, 10.1016/j.neuron.2010.09.009. 21092857 PMC2989827

[64] B. R. Stockwell , J. P. F. Angeli , H. Bayir , et al., “Ferroptosis: A Regulated Cell Death Nexus Linking Metabolism,” Redox Biology and Disease Cell 171 (2017): 273–285, 10.1016/j.cell.2017.09.021. 28985560 PMC5685180

[65] J. P. Friedmann Angeli , M. Schneider , B. Proneth , et al., “Inactivation of the Ferroptosis Regulator Gpx4 Triggers Acute Renal Failure in Mice,” Nature Cell Biology 16 (2014): 1180–1191, 10.1038/ncb3064. 25402683 PMC4894846

[66] H. Nobuta , N. Yang , Y. H. Ng , et al., “Oligodendrocyte Death in Pelizaeus‐Merzbacher Disease Is Rescued by Iron Chelation,” Cell Stem Cell 25 (2019): 531–541, 10.1016/j.stem.2019.09.003. 31585094 PMC8282124

[67] Q. Cui , H. Bi , Z. Lv , et al., “Diverse CMT2 Neuropathies Are Linked to Aberrant G3BP Interactions in Stress Granules,” Cell 186 (2023): 803–820, 10.1016/j.cell.2022.12.046. 36738734

[68] L. P. Wan , Y. Li , S. Zhao , et al., “The Pathogenic Factor of ZC4H2‐associated Rare Disorder Is a Postsynaptic Regulator for Synaptic Activity and Cognitive Function,” Proceedings of the National Academy of Sciences 122 (2025): 2426375122, 10.1073/pnas.2426375122. PMC12280958 40632560

[69] C. Espejo‐Serrano , C. Aitken , B. F. Tan , et al., “Chromatin Targeting of the RNF12/RLIM E3 Ubiquitin Ligase Controls Transcriptional Responses,” Life Science Alliance 7 (2024): 202302282, 10.26508/lsa.202302282. PMC10781586 38199845

[70] Y. R. Her and I. K. Chung , “Ubiquitin Ligase RLIM Modulates Telomere Length Homeostasis through a Proteolysis of TRF1,” Journal of Biological Chemistry 284 (2009): 8557–8566, 10.1074/jbc.M806702200. 19164295 PMC2659214

[71] L. Zhang , H. Huang , F. Zhou , et al., “RNF12 controls Embryonic Stem Cell Fate and Morphogenesis in Zebrafish Embryos by Targeting Smad7 for Degradation,” Molecular Cell 46 (2012): 650–661, 10.1016/j.molcel.2012.04.003. 22560923

[72] J.‐O. Jin , G. D. Lee , S. H. Nam , et al., “Sequential Ubiquitination of p53 by TRIM28, RLIM, and MDM2 in Lung Tumorigenesis, RLIM, and MDM2 in Lung Tumorigenesis,” Cell Death & Differentiation 28 (2020): 1790–1803, 10.1038/s41418-020-00701-y. 33328571 PMC8184939

[73] Y. Li , C. Yang , H. Wang , et al., “Sequential Stabilization of RNF220 by RLIM and ZC4H2 during Cerebellum Development and Shh‐group Medulloblastoma Progression,” Journal of Molecular Cell Biology 14 (2022): mjab082, 10.1093/jmcb/mjab082. 35040952 PMC8982406

[74] Y. Kulathu and D. Komander , “Atypical Ubiquitylation — The Unexplored World of Polyubiquitin beyond Lys48 and Lys63 Linkages,” Nature Reviews Molecular Cell Biology 13 (2012): 508–523, 10.1038/nrm3394. 22820888

[75] R. Yau and M. Rape , “The Increasing Complexity of the Ubiquitin Code,” Nature Cell Biology 18 (2016): 579–586, 10.1038/ncb3358. 27230526

[76] F. Ohtake and H. Tsuchiya , “The Emerging Complexity of Ubiquitin Architecture,” Journal of Biochemistry 161 (2016): 125–133, 10.1093/jb/mvw088. 28011818

[77] J. Deng , X. Lin , J. Qin , et al., “SPTBN2 suppresses Ferroptosis in NSCLC Cells by Facilitating SLC7A11 Membrane Trafficking and Localization,” Redox Biology 70 (2024): 103039, 10.1016/j.redox.2024.103039. 38241838 PMC10825533

[78] V. Gallo and B. Deneen , “Glial Development: The Crossroads of Regeneration and Repair in the CNS,” Neuron 83 (2014): 283–308, 10.1016/j.neuron.2014.06.010. 25033178 PMC4114724

[79] J. Niu , G. Yu , X. Wang , et al., “Oligodendroglial Ring Finger Protein Rnf43 Is an Essential Injury‐specific Regulator of Oligodendrocyte Maturation,” Neuron 109 (2021): 3104–3118, 10.1016/j.neuron.2021.07.018. 34390652 PMC8547708

[80] X. Chen , R. Kang , G. Kroemer , and D. Tang , “Ferroptosis in Infection, Inflammation, and Immunity,” Journal of Experimental Medicine 218 (2021): 20210518, 10.1084/jem.20210518. PMC8126980 33978684

[81] G. Lei , L. Zhuang , and B. Gan , “The Roles of Ferroptosis in Cancer: Tumor Suppression, Tumor Microenvironment, and Therapeutic Interventions,” Cancer Cell 42 (2024): 513–534, 10.1016/j.ccell.2024.03.011. 38593779

[82] K. Newton , A. Strasser , N. Kayagaki , and V. M. Dixit , “Cell Death,” Cell 187 (2024): 235–256, 10.1016/j.cell.2023.11.044. 38242081

[83] H. Sies , “Oxidative Stress: A Concept in Redox Biology and Medicine,” Redox Biology 4 (2015): 180–183, 10.1016/j.redox.2015.01.002. 25588755 PMC4309861

[84] F. Lin , W. Chen , J. Zhou , et al., “Mesenchymal Stem Cells Protect against Ferroptosis via Exosome‐mediated Stabilization of SLC7A11 in Acute Liver Injury,” Cell Death & Disease 13 (2022): 271, 10.1038/s41419-022-04708-w. 35347117 PMC8960810

[85] Y. Yang , Y. Ma , Q. Li , et al., “STAT6 inhibits Ferroptosis and Alleviates Acute Lung Injury via Regulating P53/SLC7A11 Pathway,” Cell Death & Disease 13 (2022): 530, 10.1038/s41419-022-04971-x. 35668064 PMC9169029

[86] J. Yin , J. Chen , J. H. Hong , et al., “4EBP1‐mediated SLC7A11 Protein Synthesis Restrains Ferroptosis Triggered by MEK Inhibitors in Advanced Ovarian Cancer,” JCI Insight 9 (2024): 177857, 10.1172/jci.insight.177857. PMC11383183 38842940

[87] Y. Shi , Q. Tang , S. Sheng , et al., “PSMD14 Stabilizes SLC7A11 to Ameliorate Glucocorticoid‐Induced Osteoporosis by Suppressing Osteocyte Ferroptosis,” Advanced Science 12 (2025): 14902, 10.1002/advs.202414902. PMC12376700 40444470

[88] L. Wang , Y. Liu , T. Du , et al., “ATF3 promotes Erastin‐induced Ferroptosis by Suppressing System Xc–,” Cell Death & Differentiation 27 (2019): 662–675, 10.1038/s41418-019-0380-z. 31273299 PMC7206049

[89] H. Dong , Y. Xia , S. Jin , et al., “Nrf2 Attenuates Ferroptosis‐Mediated IIR‐ALI by Modulating TERT and SLC7A11,” Cell Death & Disease 12 (2021): 1027, 10.1038/s41419-021-04307-1. 34716298 PMC8556385

[90] A. J. George , Y. C. Hoffiz , A. J. Charles , Y. Zhu , and A. M. Mabb , “A Comprehensive Atlas of E3 Ubiquitin Ligase Mutations in Neurological Disorders,” Frontiers in Genetics 9 (2018): 29, 10.3389/fgene.2018.00029. 29491882 PMC5817383

[91] P. Ma and B. Mao , “The Many Faces of the E3 Ubiquitin Ligase, RNF220, in Neural Development and Beyond,” Development, Growth & Differentiation 64 (2021): 98–105, 10.1111/dgd.12756. 34716995

[92] A. Sferra , P. Fortugno , M. Motta , et al., “Biallelic Mutations in RNF220 Cause Laminopathies Featuring Leukodystrophy, Ataxia and Deafness,” Brain 144 (2021): 3020–3035, 10.1093/brain/awab185. 33964137

[93] M. Hale and G. J. Bashaw , “Emerging Roles for E3 Ubiquitin Ligases in Neural Development and Disease,” Frontiers in Cell and Developmental Biology 13 (2025): 1557653, 10.3389/fcell.2025.1557653. 40496139 PMC12149146

[94] F. Bustos , A. Segarra‐Fas , V. K. Chaugule , et al., “RNF12 X‐Linked Intellectual Disability Mutations Disrupt E3 Ligase Activity and Neural Differentiation,” Cell Reports 23 (2018): 1599–1611, 10.1016/j.celrep.2018.04.022. 29742418 PMC5976579

[95] F. Bustos , A. Segarra‐Fas , G. Nardocci , et al., “Functional Diversification of SRSF Protein Kinase to Control Ubiquitin‐Dependent Neurodevelopmental Signaling,” Developmental Cell 55 (2020): 629–647, 10.1016/j.devcel.2020.09.025. 33080171 PMC7725506

[96] F. Wang , J. Shin , J. M. Shea , et al., “Regulation of X‐linked Gene Expression during Early Mouse Development by Rlim,” Elife 5 (2016): 19127, 10.7554/eLife.19127. PMC5059138 27642011

[97] S. A. Johnsen , C. Güngör , T. Prenzel , et al., “Regulation of Estrogen‐dependent Transcription by the LIM Cofactors CLIM and RLIM in Breast Cancer,” Cancer Research 69 (2009): 128–136, 10.1158/0008-5472.CAN-08-1630. 19117995 PMC2713826

[98] Y. Zhang , Z. Song , R. Wu , et al., “PRRC2B modulates Oligodendrocyte Progenitor Cell Development and Myelination by Stabilizing Sox2 mRNA,” Cell Reports 43 (2024): 113930, 10.1016/j.celrep.2024.113930. 38507412

[99] P. Ma , N.‐N. Song , Y. Li , et al., “Fine‐Tuning of Shh/Gli Signaling Gradient by Non‐proteolytic Ubiquitination during Neural Patterning,” Cell Reports 28 (2019): 541–553.e4, 10.1016/j.celrep.2019.06.017. 31291587

[100] M. Zhang , J. Wang , K. Zhang , et al., “Ten‐eleven Translocation 1 Mediated‐DNA Hydroxymethylation Is Required for Myelination and Remyelination in the Mouse Brain,” Nature Communications 12 (2021): 5091, 10.1038/s41467-021-25353-5. PMC8385008 34429415

[101] P. Ma , X. Yang , Q. Kong , et al., “The Ubiquitin Ligase RNF220 Enhances Canonical Wnt Signaling through USP7‐Mediated Deubiquitination of β‐Catenin,” Molecular and Cellular Biology 34 (2014): 4355–4366, 10.1128/MCB.00731-14. 25266658 PMC4248747

[102] X. Guo , P. Ma , Y. Li , et al., “RNF220 Mediates K63‐Linked Polyubiquitination of STAT1 and Promotes Host Defense,” Cell Death & Differentiation 28 (2020): 640–656, 10.1038/s41418-020-00609-7. 32814877 PMC7862670

[103] Q. Kong , W. Zeng , J. Wu , W. Hu , C. Li , and B. Mao , “RNF220, an E3 Ubiquitin Ligase That Targets Sin3B for Ubiquitination,” Biochemical and Biophysical Research Communications 393 (2010): 708–713, 10.1016/j.bbrc.2010.02.066. 20170641

[104] T. C. Branon , J. A. Bosch , A. D. Sanchez , et al., “Efficient Proximity Labeling in Living Cells and Organisms with TurboID,” Nature Biotechnology 36 (2018): 880–887, 10.1038/nbt.4201. PMC6126969 30125270

[105] P. Ma , Y. Li , H. Wang , and B. Mao , “Haploinsufficiency of the TDP43 Ubiquitin E3 Ligase RNF220 Leads to ALS‐Like Motor Neuron Defects in the Mouse,” Journal of Molecular Cell Biology 13 (2021): 374–382, 10.1093/jmcb/mjaa072. 33386850 PMC8373269

