# Supplementary material for: The E3 Ubiquitin Ligase RLIM Safeguards Oligodendrocyte Development and Myelination by Targeting SLC7A11 for Polyubiquitination to Regulate Ferroptotic Resistance
Source: Adv Sci (Weinh). 2026 Jul 23:e76315. Online ahead of print. doi: 10.1002/advs.76315 (PMC13395749; doi:10.1002/advs.76315)
Supplement: Supplementary file 1 — Supporting File 1: advs76315‐sup‐0001‐SuppMat.docx. [file ADVS-9999-e76315-s003.docx]

***Supplementary Information***

**The E3 ubiquitin ligase RLIM safeguards oligodendrocyte development and myelination by targeting SLC7A11 for polyubiquitination to regulate ferroptotic resistance**

Yuwei Li^#^, Haijun Zhang^#^, Jieya Zhou, Shiyu Yang, Jingmei Cha, Gaofeng Yan, Shuhua Zhao*, Bingyu Mao*, and Pengcheng Ma*

1. Supplementary figures and tables list………….………………………..…Page 2

2. Supplementary Figures and Figure Legends………….……….………..…Page 3-34

3. Supplementary Table Legends………….……………….…………....……Page 35

**Supplementary figures and tables list:**

Supplementary Figure 1, related to Figure 1.

Supplementary Figure 2, related to Figure 1.

Supplementary Figure 3, related to Figure 2.

Supplementary Figure 4, related to Figure 4.

Supplementary Figure 5, related to Figure 4.

Supplementary Figure 6, related to Figure 4.

Supplementary Figure 7, related to Figure 4.

Supplementary Figure 8, related to Figure 4.

Supplementary Figure 9, related to Figure 5.

Supplementary Figure 10, related to Figure 5.

Supplementary Figure 11, related to Figure 5.

Supplementary Figure 12, related to Figure 5.

Supplementary Figure 13, related to Figure 5.

Supplementary Figure 14, related to Figure 6.

Supplementary Figure 15, related to Figure 6.

Supplementary Figure 16, related to Figure 7.

Supplementary Table 1, related to Figure 4.

Supplementary Table 2, related to Figure 4.

Supplementary Table 3, related to Figure 5


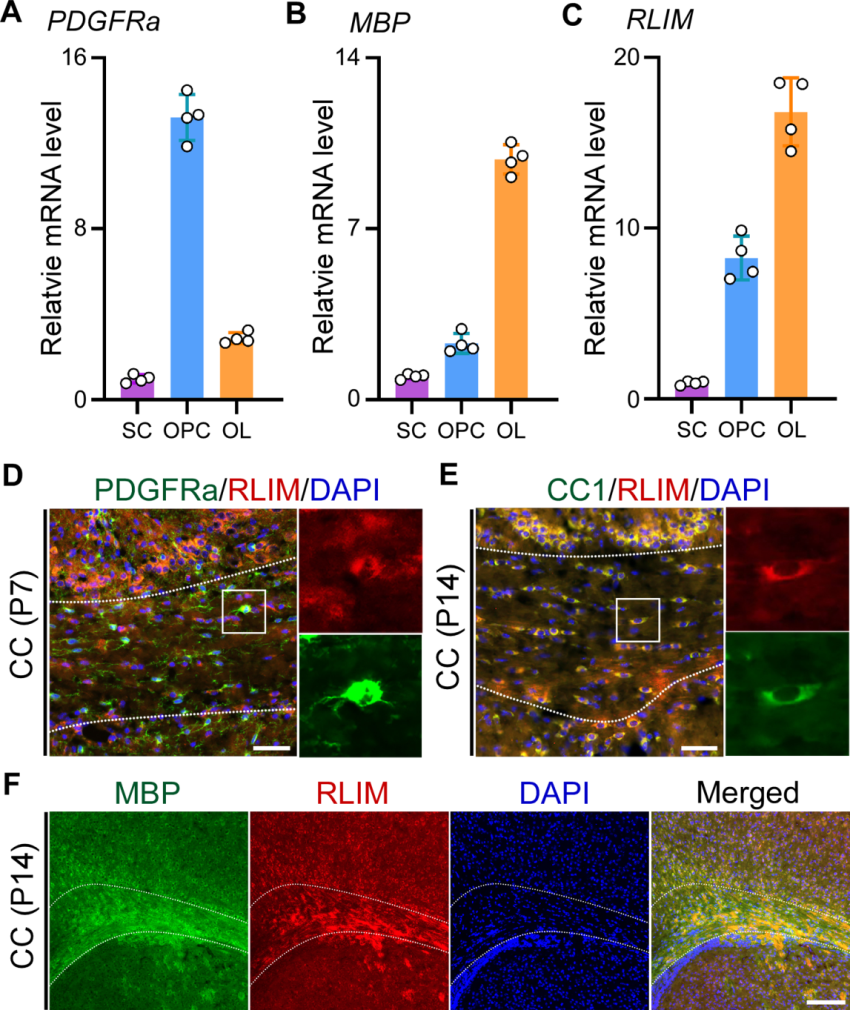


**Figure S1.** Expression of RLIM in OL lineage cells of mouse brains. (A-C) RT-qPCR results showing the relative expression levels of *PDGFRα* (A, SC: 1.000 ± 0.1719, OPC: 13.22 ± 1.074, OL: 2.878 ± 0.2599), *MBP* (B, SC: 1.000 ± 0.09323, OPC: 2.305 ± 0.4112, OL: 9.847 ± 0.5979), and *RLIM* (C, SC: 1.000 ± 0.05408, OPC: 8.261 ± 1.280, OL: 16.82 ± 1.993) in the indicated cells isolated from cortical tissue of wild-type P10 mice. The expression of the indicated genes in the mixed single cells were set to 1. *β-ACTIN* was used as an internal control. (D) Immunofluorescence staining assays show RLIM expression in PDGFRα^+^ OPC cells in corpus callosum regions of wild-type P7 mouse brains. Scale bar, 50 μm. (E) Immunofluorescence staining assays show RLIM expression in CC1^+^ OL cells in corpus callosum region of P14 mouse brains. Scale bar, 50 μm. (F) Immunofluorescence staining assays show RLIM expression in MBP^+^ mature OL cells in corpus callosum region of P21 mouse brains. Scale bar, 150 μm. CC, corpus callosum. SC, mixed single cells; OPC, oligodendrocyte precursor cells isolated by PDGFRα^+^ beads; OL, oligodendrocytes isolated by O4^+^ beads.


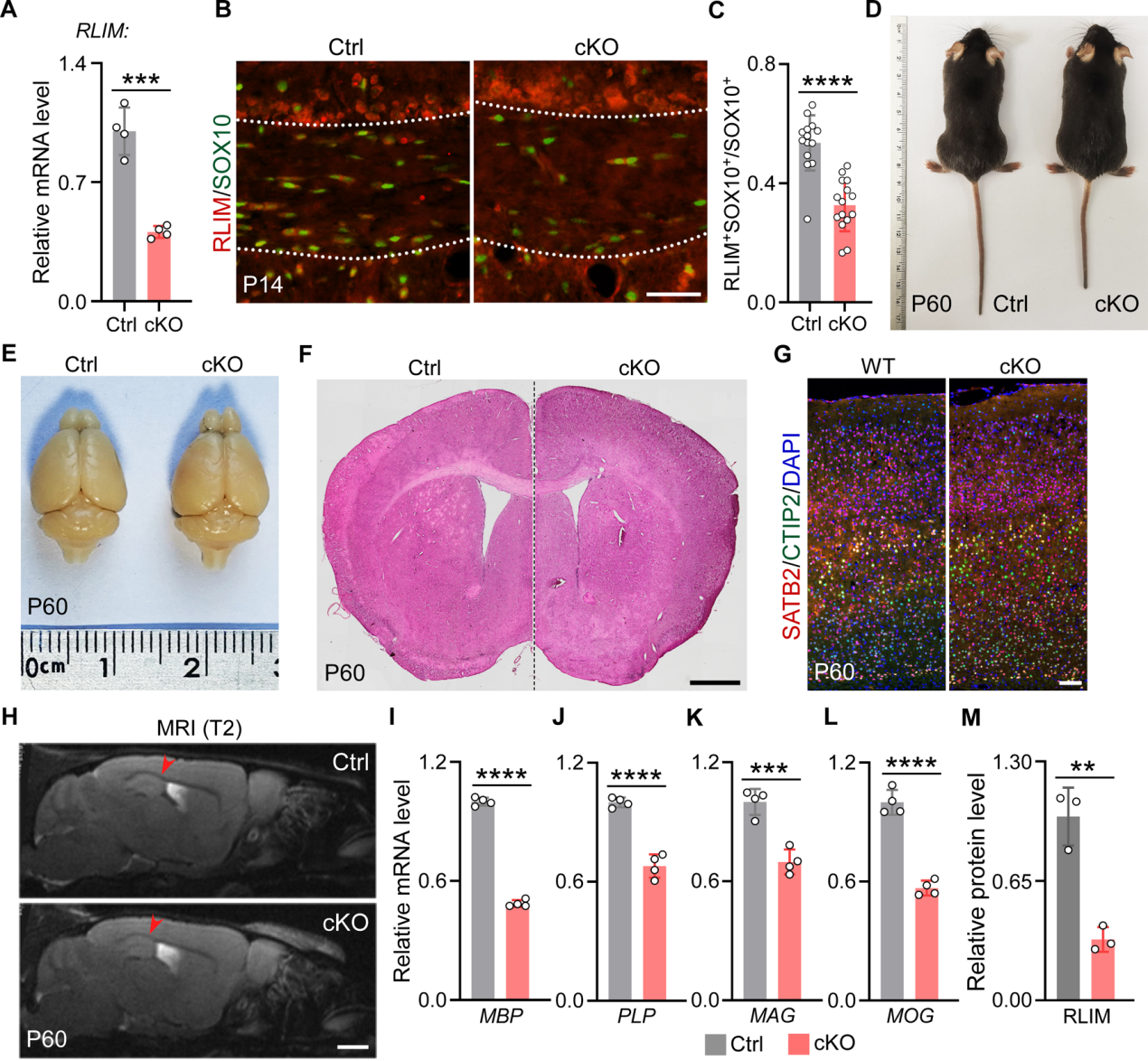


**Figure S2.** RLIM expression and brain morphology of the *RLIM* cKO mice. (A) RT-qPCR analysis of the expression of *RLIM* (control: 1.000 ± 0.1393, *RLIM* cKO: 0.4079 ± 0.03508) in cortical tissues of control (n = 3) and the *RLIM* cKO (n = 3) mice at P28. *β-ACTIN* was used as an internal control and the expression level of *RLIM* in the control group was set to 1. Unpaired two-tailed Student’s *t* test. (B) Representative images of immunofluorescence staining assays showing RLIM and SOX10 expression in the corpus callosum of control (n = 3) and the *RLIM* cKO (n = 3) mice at P7. Scale bar, 50 μm. (C) The bar graph shows the quantification of the percentage of RLIM^+^SOX10^+^ among SOX10^+^ cells in (B) (control: 0.5355 ± 0.09304 and *RLIM* cKO: 0.3264 ± 0.08735). Unpaired two-tailed Student’s *t* test. (D) Representative gross view of control and the *RLIM* cKO mice at P60. (E) Representative brain morphology of control and the *RLIM* cKO mice at P60. (F) Hematoxylin and eosin (H&E) staining of coronal brain slices shows the brain morphology of control and the *RLIM* cKO mice at P60. Scale bar, 1 mm. (G) Immunofluorescence staining analysis of CTIP2 and SATB2 shows the patterning of cerebral cortex layers in the cortices of control and the *RLIM* cKO mice at P60. Scale bar, 100 μm. (H) Representative T2-weighted mouse brain MRI sagittal scanning of P60 control and the *RLIM* cKO mice. The red arrows indicate the corpus callosum. Scale bar, 2 mm. (I-L) RT-qPCR analysis of the expression levels (mean ± SD) of myelin related genes, including *MBP* (control: 1.000 ± 0.01984 and *RLIM* cKO: 0.4862 ± 0.01758), *PLP* (control: 1.000 ± 0.02460 and *RLIM* cKO: 0.6794 ± 0.05890), *MAG* (control: 1.000 ± 0.06552 and *RLIM* cKO: 0.6980 ± 0.06250), and *MOG* (control: 1.000 ± 0.06291 and *RLIM* cKO: 0.5679 ± 0.03750), in cortical tissues of Control (n = 4) and the *RLIM* cKO (n = 4) mice at P28. *β-ACTIN* was used as an internal control. The expression level of each gene in the control group was set to 1. Unpaired two-tailed Student’s *t* test; **, *p* < 0.01; ****, *p* < 0.0001. (M) The bar graph shows the quantification of RLIM protein levels (control: 1.000 ± 0.1578 and *RLIM* cKO: 0.3321 ± 0.06742) in cortical tissues of control (n = 3) and the *RLIM* cKO (n = 3) mice in (Figure 1I). Ctrl, control; cKO, *RLIM* cKO; MRI, magnetic resonance imaging.


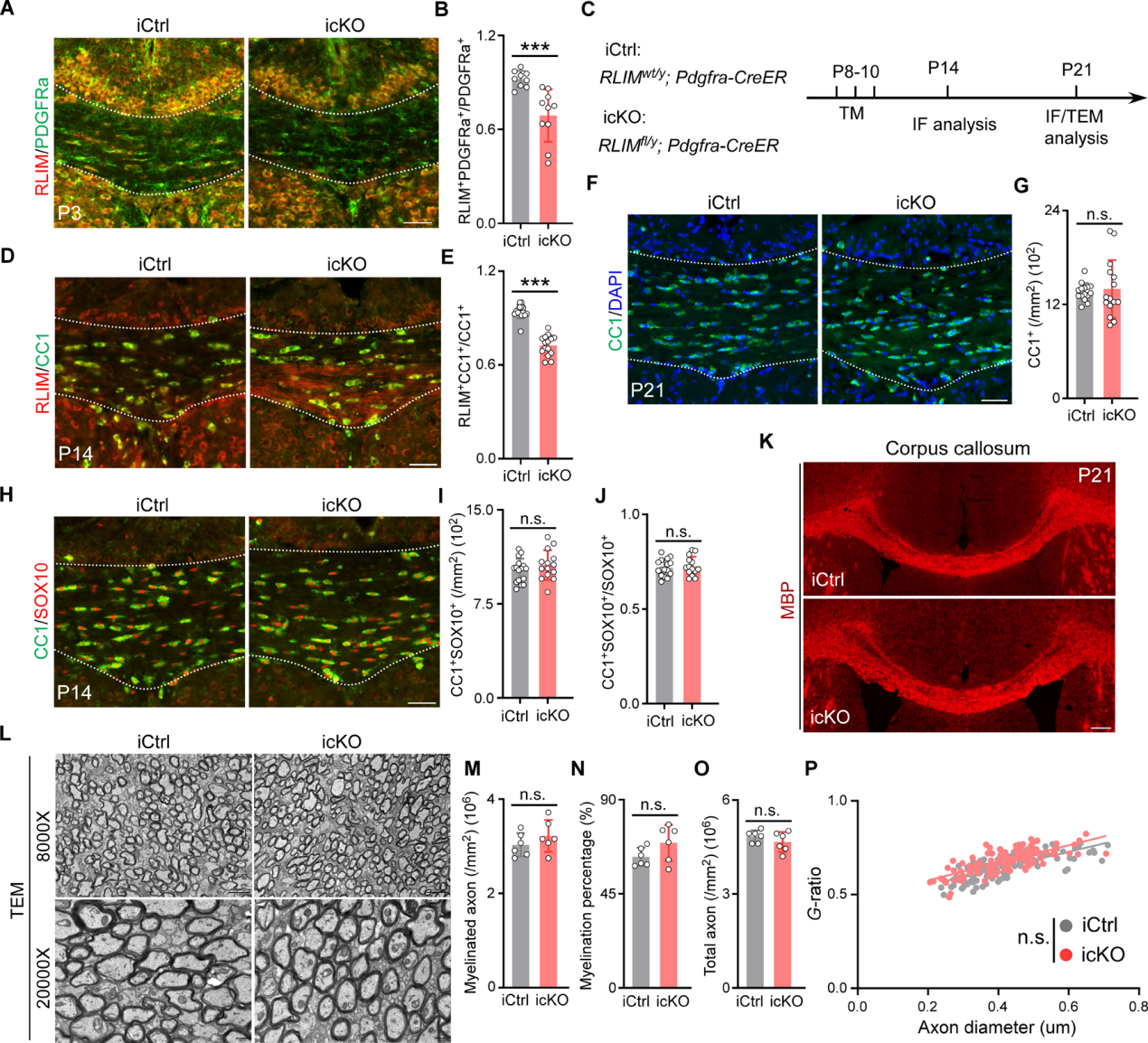


**Figure S3.** RLIM is not required for OL differentiation. (A) Representative images of immunofluorescence staining assays showing RLIM and PDGFRα expression in the corpus callosum of icontrol (n = 3) and the *RLIM* icKO (n = 3) mice at P3. Scale bar, 50 μm. (B) The bar graph shows the quantification of the percentage of RLIM^+^PDGFRα^+^ among PDGFRα^+^ cells in (A) (icontrol: 0.9217 ± 0.05234 and *RLIM* icKO: 0.6887 ± 0.1676). Unpaired two-tailed Student’s *t* test; ***, *p* < 0.001. (C) Schematic diagram of tamoxifen induced knockout and the following analysis for icontrol and *RLIM* icKO mice. (D) Representative images of immunofluorescence staining assays showing RLIM and CC1 expression in the corpus callosum of icontrol (n = 3) and the *RLIM* icKO (n = 3) mice at P14. Scale bar, 50 μm. (E) The bar graph shows the quantification of the percentage of RLIM^+^CC1^+^ among CC1^+^ cells in (D) (icontrol: 0.9422 ± 0.04393 and *RLIM* icKO: 0.7255 ± 0.06499). Unpaired two-tailed Student’s *t* test; ***, *p* < 0.001. (F) Representative images of immunofluorescence staining assays showing CC1 expression in the corpus callosum of icontrol (n = 3) and the *RLIM* icKO (n = 3) mice at P21. Scale bar, 50 μm. (G) The bar graph shows the quantification of the number of CC1^+^ cells in (F) (icontrol: 1360 ± 112.6 /mm^2^ and *RLIM* icKO: 1398 ± 369.1 /mm^2^). Unpaired two-tailed Student’s *t* test; n.s. not significant. (H) Representative images of immunofluorescence staining assays showing SOX10 and CC1 expression in the corpus callosum of icontrol (n = 3) and the *RLIM* icKO (n = 3) mice at P14. Scale bar, 50 μm. (I, J) The bar graph shows the quantification of SOX10^+^CC1^+^ cells (I) (icontrol: 1016 ± 95.74 /mm^2^ and *RLIM* icKO: 1060 ± 118.2 /mm^2^) or the percentage of SOX10^+^CC1^+^ among CC1^+^ cells (J) (icontrol: 0.7186 ± 0.04496 and *RLIM* icKO: 0.7266 ± 0.05053) in (H) . Unpaired two-tailed Student’s *t* test; n.s. not significant. (K) Representative images of immunofluoresence staining assays showing the expression of MBP in the corpus callosum of icontrol and the *RLIM* icKO mice at P21. Scale bar, 400 μm. (L) Transmission electron microscopy (TEM) images of the corpus callosum transverse sections from P21 icontrol (n = 3) and the *RLIM* icKO (n = 3) mice. Scale bars, 5 μm for the upper panels and 1 μm for the lower panels. (M) Bar graphs (mean ± SD) showing quantification of the number of myelinated axons in (L) (icontrol, 3031800 ± 249190 /mm^2^ and *RLIM* icKO, 3220445 ± 336912 /mm^2^). Unpaired two-tailed Student’s *t* test; n.s. not significant. (N) Bar graphs (mean ± SD) show quantification of the percentage of myelinated axons in (L) (icontrol, 62.67 ± 4.385 % and *RLIM* icKO, 69.37 ± 8.632 %). Unpaired two-tailed Student’s *t* test; n.s. not significant. (M) Bar graphs (mean ± SD) showing quantification of the total axon number in (L) (icontrol: 4837105 ± 194147 /mm^2^ and *RLIM* icKO: 4662235 ± 317956 /mm^2^). Unpaired two-tailed Student’s *t* test; n.s. not significant. (P) Scatterplots show the myelin *g*-ratios (diameter of axon/diameter of entire fiber). At least 100 axons from 3 mice were involved in the analysis for each group. General linear regression model and ANCOVA analysis; n.s. not significant. iCtrl, icontrol; icKO, *RLIM* icKO; IF, immunofluresence; TEM, transmission electron micorscopy; TM, tamoxifen.


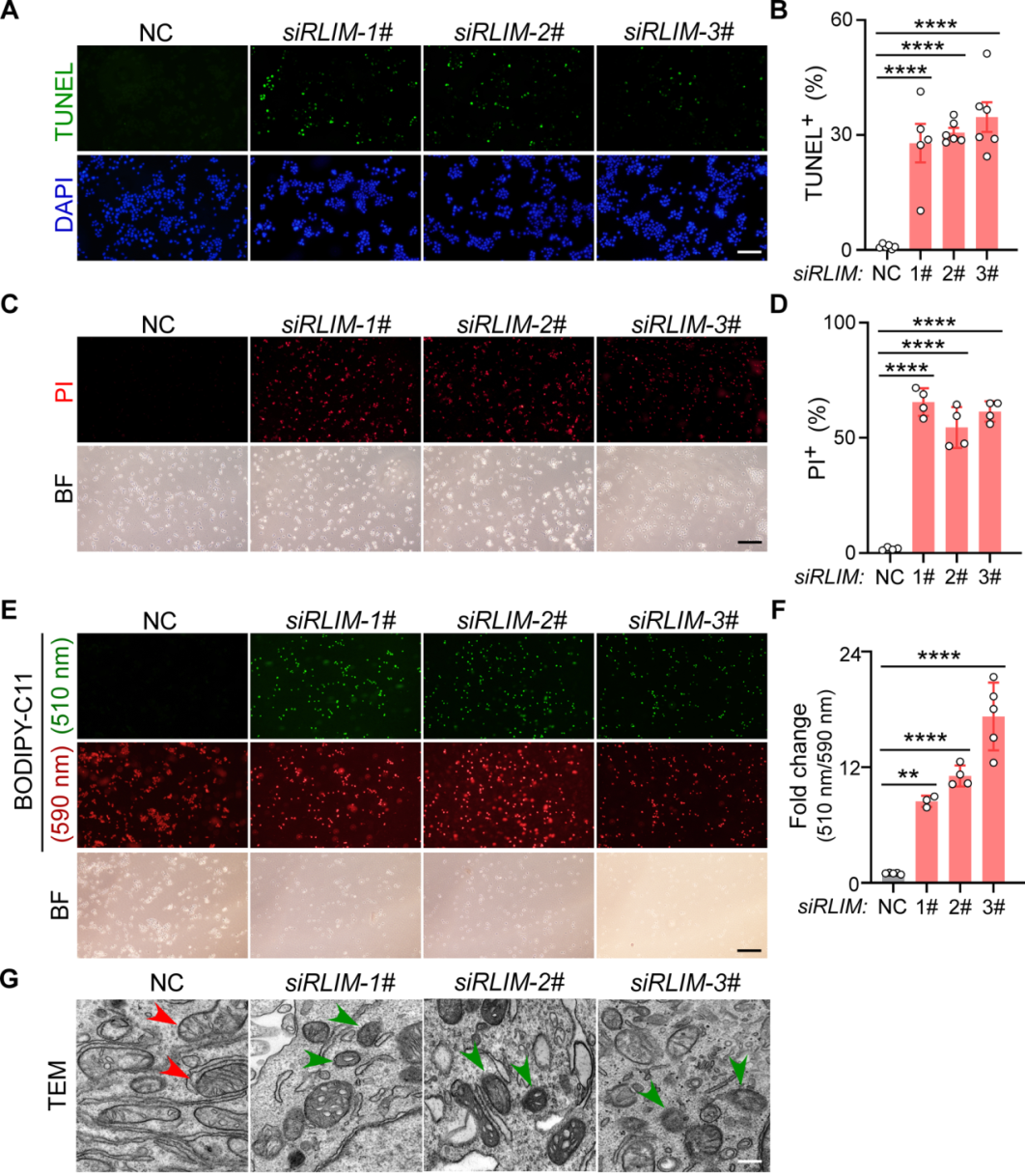


**Figure S4.** RLIM deficiency leads to ferroptosis in MOPC cells. (A) The representative images of TUNEL staining for MOPC cells transfected with control or *RLIM* siRNAs. Scale bar, 250 μm. (B) The bar graph shows the quantification of the percentage of TUNEL^+^ cells in each group in (A) (NC: n = 6, 0.8982 ± 0.5464 %; *RLIM* siRNA-1#: n = 5, 27.90 ± 11.25 %; *RLIM* siRNA-2#: n = 6, 30.69 ± 2.858 %; *RLIM* siRNA-3#: n = 6, 34.72 ± 9.502 %). One-way ANOVA with multiple comparisons; ****, *p* < 0.0001. (C) The representative images of PI staining for MOPC cells transfected with control or *RLIM* siRNAs. Scale bar, 250 μm. (D) The bar graph shows the quantification of the percentage of PI^+^ cells in each group in (C) (NC: n = 4, 1.853 ± 0.6158 %; *RLIM* siRNA-1#: n = 4, 65.53 ± 5.986 %; *RLIM* siRNA-2#: n = 4, 54.50 ± 8.890 %; *RLIM* siRNA-3#: n = 4, 61.43 ± 4.420 %). One-way ANOVA with multiple comparisons; ****, *p* < 0.0001. (E) The representative images show lipid ROS levels measured by the BODIPY-C11 probe in MOPC cells transfected with control or *RLIM* siRNAs. Scale bar, 250 μm. (F) The bar graph shows the quantification of lipid ROS levels (BODIPY-C11_510 nm_^+^ cells %/BODIPY-C11_590 nm_^+^ cells %) in each group in (E) (NC: n = 5, 1.000 ± 0.07596; *RLIM* siRNA-1#: n = 3, 8.468 ± 0.5869; *RLIM* siRNA-2#: n = 4, 11.10 ± 1.087; *RLIM* siRNA-3#: n = 5, 17.26 ± 3.518). The lipid ROS level of the control group was set to 1. One-way ANOVA with multiple comparisons; **, *p* < 0.01; ****, *p* < 0.0001. (G) MOPC cells transfected with the indicated siRNAs were subjected to transmission electron microscopy. Red arrows indicated mitochondria with obvious cristae, while green arrows represent shrunken mitochondria. Scale bar, 280 nm. NC, negative control; BF, bright field; TEM, transmission electron microscopy.


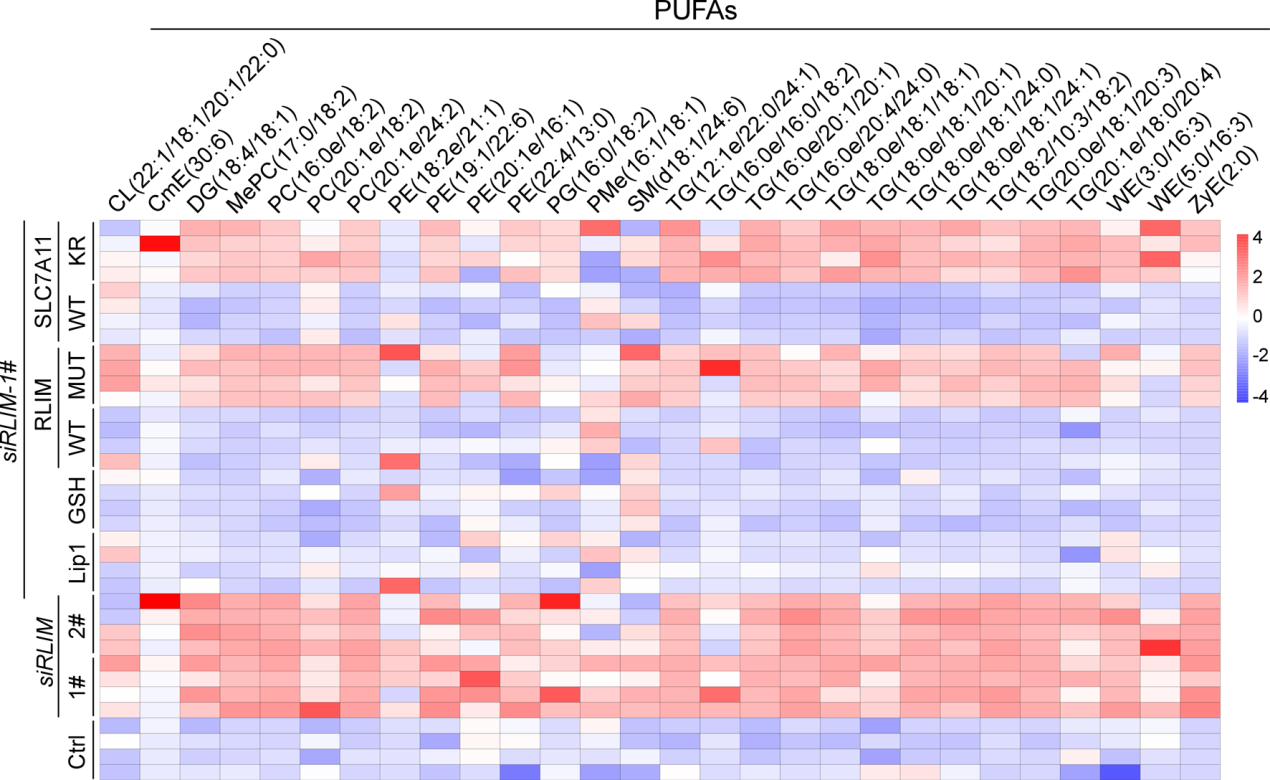


**Figure S5.** Lipidomic analysis of the quantification of lipid metabolites in the indicated MOPC cells. Heatmap shows the relative levels of PUFAs in the indicated groups (n = 4 for each group). The PUFAs increased in the *RLIM* knockdown MOPC cells were shown. PUFA, polyunsaturated fatty acids; PE, phosphatidylethanolamine; PC, phosphatidylcholine; TG, triacylglycerol; Cer, ceramide; WT, wildtype; Mut, ligase dead mutant; KR, K475R.


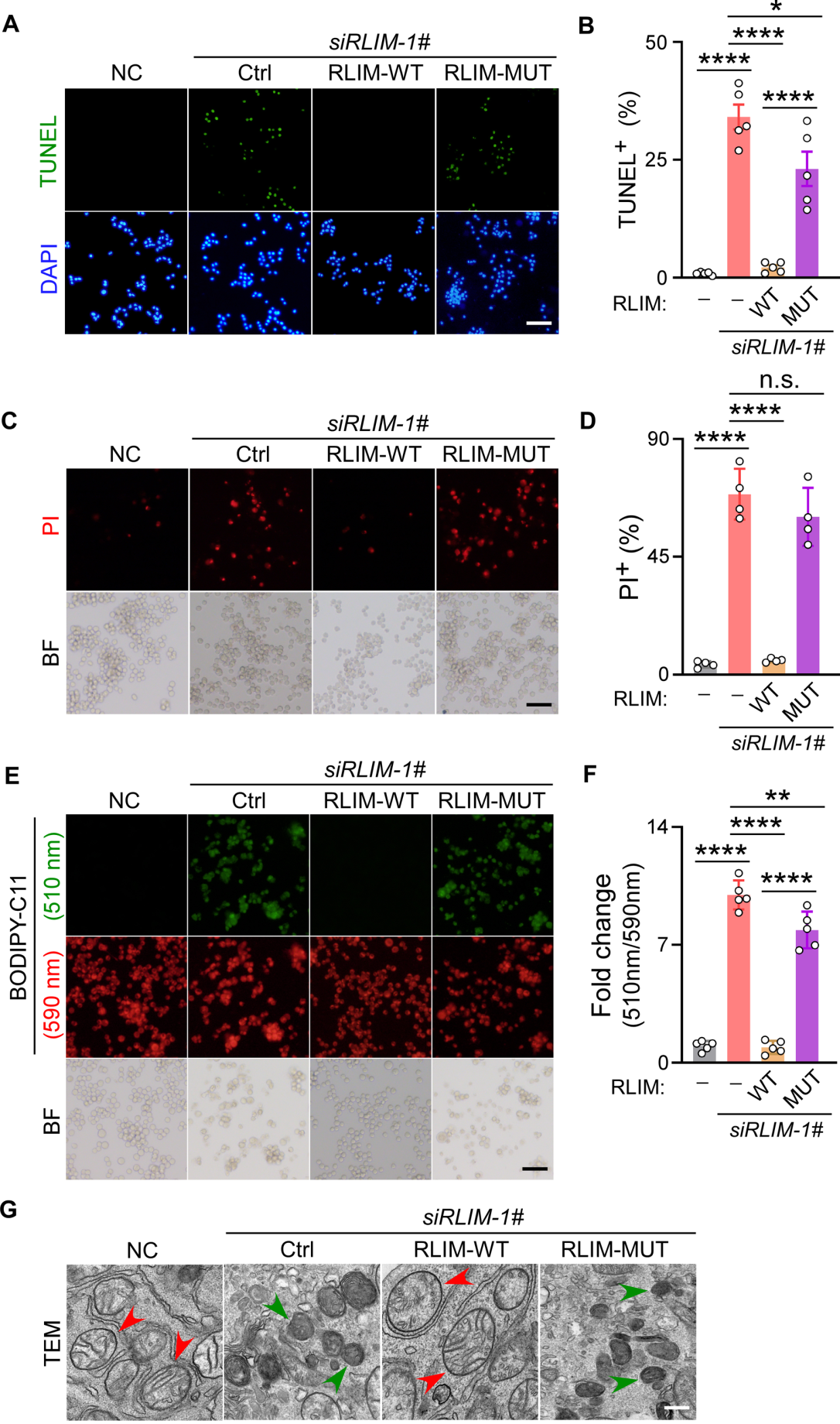


**Figure S6.** Expression of RLIM wild-type, but not its ligase dead mutant, resuced RLIM kockdown induced ferroptosis in MOPC cells. (A) The representative images of TUNEL staining for the indicated MOPC cells overexpressed with RLIM wild-type or its ligase dead mutant. Scale bar, 250 μm. (B) The bar graph shows the quantification of the percentage of TUNEL^+^ cells in each group in (A) (NC: n = 5, 0.8940 ± 0.3340 %; *RLIM* siRNA-1#: n = 5, 34.10 ± 5.843 %; *RLIM* siRNA-1#-RLIM WT: n = 5, 2.148 ± 1.070 %; *RLIM* siRNA-1#-RLIM MUT: n = 5, 23.08 ± 8.168 %). One-way ANOVA with multiple comparisons; *, *p* < 0.05; ****, *p* < 0.0001. (C) The representative images of PI staining for the indicated MOPC cells overexpressed with RLIM wild-type or its ligase dead mutant. Scale bar, 250 μm. (D) The bar graph shows the quantification of the percentage of PI^+^ cells in each group in (C) (NC: n = 4, 3.905 ± 1.227 %; *RLIM* siRNA-1#: n = 4, 68.91 ± 9.706 %; *RLIM* siRNA-1#-RLIM WT: n = 4, 5.438 ± 0.7527 %; *RLIM* siRNA-1#-RLIM MUT: n = 4, 60.25 ± 11.11 %). One-way ANOVA with multiple comparisons; ****, *p* < 0.001; n.s., not significant. (E) The representative images show lipid ROS levels measured by the BODIPY-C11 probe in the indicated MOPC cells overexpressed with RLIM wildtype or its ligase dead mutant. Scale bar, 250 μm. (F) The bar graph shows the quantification of lipid ROS levels (BODIPY-C11_510 nm_^+^ cells %/BODIPY-C11_590 nm_^+^ cells %) in each group in (E) (NC: n = 5, 1.000 ± 0.2939; *RLIM* siRNA-1#: n = 5, 9.968 ± 0.8612; *RLIM* siRNA-1#-RLIM WT: n = 5, 0.9120 ± 0.3884; *RLIM* siRNA-1#-RLIM MUT: n = 5, 7.884 ± 1.090). The lipid ROS level of the control group was set to 1. One-way ANOVA with multiple comparisons; **, *p* < 0.01; ****, *p* < 0.0001. (G) The indicated MOPC cells overexpressed with RLIM wild-type or its ligase dead mutant were subjected to transmission electron microscopy. Red arrows indicated mitochondria with obvious cristae, while green arrows represent shrunkedn mitochondria. Scale bar, 280 nm. NC, negative control; Ctrl, control; WT, wild-type; MUT, ligase dead mutant; TEM, transmission electron microscopy; BF, bright field.

**
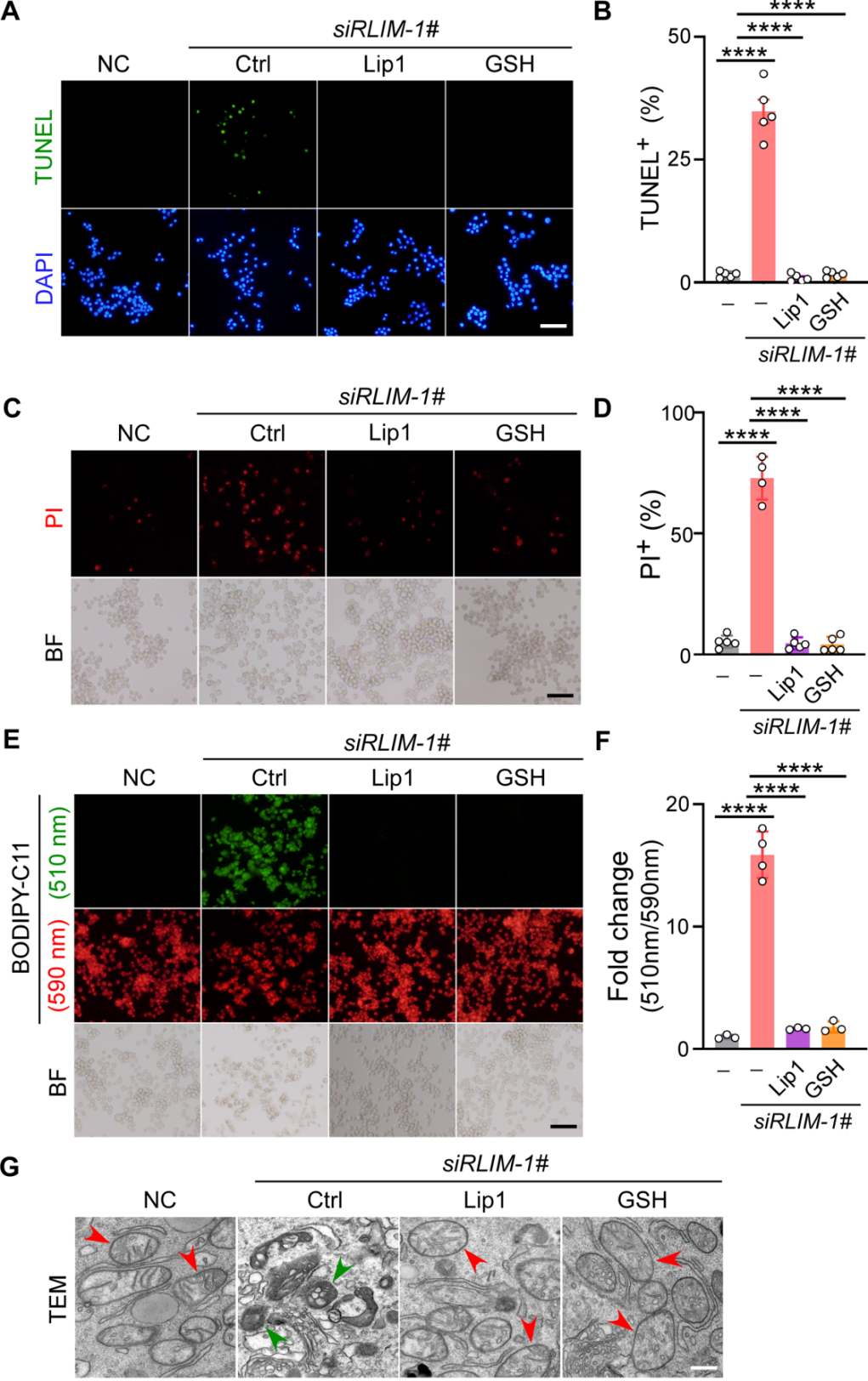
**

**Figure S7.** Lip1 and GSH treatment resuced *RLIM* kockdown induced ferroptosis in MOPC cells. (A) The representative images of TUNEL staining for the indicated MOPC cells treated with Lip1 or GSH. Scale bar, 250 μm. (B) The bar graph shows the quantification of the percentage of TUNEL^+^ cells in each group in (A) (NC: n = 5, 1.607 ± 0.6176 %; *RLIM* siRNA-1#: n = 5, 34.81 ± 5.384 %; *RLIM* siRNA-1#-Lip1: n = 5, 0.9427 ± 7534 %; *RLIM* siRNA-1#-GSH: n = 5, 1.659 ± 0.6433 %). One-way ANOVA with multiple comparisons; ****, *p* < 0.0001. (C) The representative images of PI staining for the indicated MOPC cells treated with Lip1 or GSH. Scale bar, 250 μm. (D) The bar graph shows the quantification of the percentage of PI^+^ cells in each group in (C) (NC: n = 5, 5.336 ± 2.512 %; *RLIM* siRNA-1#: n = 4, 72.83 ± 8.882 %; *RLIM* siRNA-1#-Lip1: n = 5, 4.672 ± 2.473 %; *RLIM* siRNA-1#-GSH: n = 5, 4.168 ± 3.084 %). One-way ANOVA with multiple comparisons; ****, *p* < 0.0001. (E) The representative images show lipid ROS levels measured by the BODIPY-C11 probe in the indicated MOPC cells treated with Lip1 or GSH. Scale bar, 250 μm. (F) The bar graph shows the quantification of lipid ROS levels (BODIPY-C11_510 nm_^+^ cells %/BODIPY-C11_590 nm_^+^ cells %) in each group in (E) (NC: n = 3, 1.000 ± 0.1678; *RLIM* siRNA-1#: n = 4, 15.87 ± 1.905; *RLIM* siRNA-1#-Lip1: n = 3, 1.672 ± 0.08855; *RLIM* siRNA-1#-GSH: n = 3, 1.817 ± 0.4526). The lipid ROS level of the control group was set to 1. One-way ANOVA with multiple comparisons; ****, *p* < 0.0001. (G) The indicated MOPC cells treated with Lip1 or GSH were subjected to transmission electron microscopy. Red arrows indicated mitochondria with obvious cristae, while green arrows represent shrunken mitochondria. Scale bar, 280 nm. NC, negative control; Ctrl, control; TEM, transmission electron microscopy; BF, bright field.


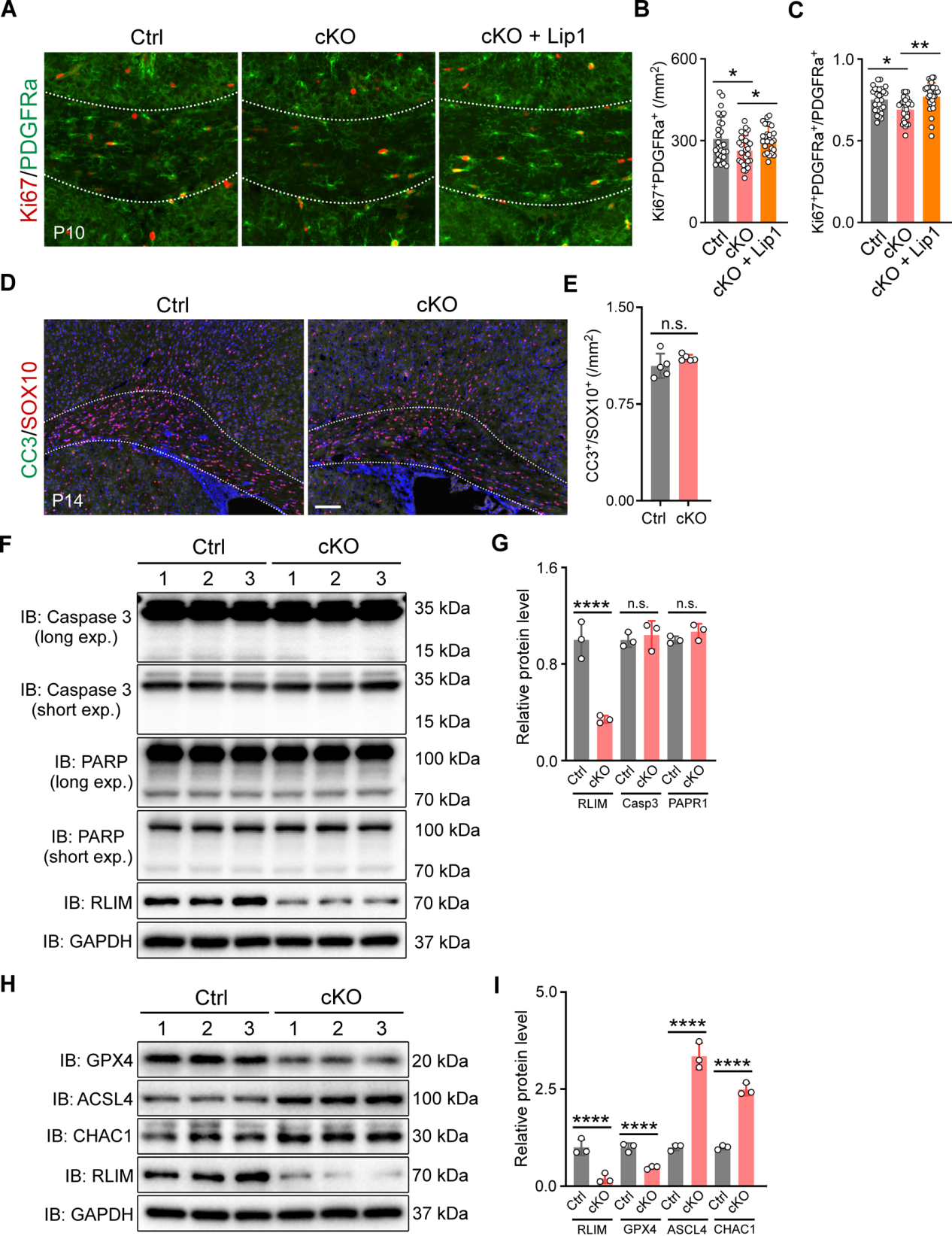


**Figure S8.** RLIM deficiency leads to ferroptosis in MOPC cells. (A) Representative images of immunofluorescence staining assays showing Ki67 and PDGFRα expression in the corpus callosum of control (n = 3), *RLIM* cKO (n = 3) or *RLIM* cKO mice with Lip1 administration (n = 3) at P10. Scale bar, 50 μm. (B) The bar graph shows the quantification of the number of KI67^+^PDGFRα^+^ cells in (L) (control: 306.9 ± 80.21 /mm^2^, *RLIM* cKO: 263.4 ±55.43 /mm^2^, and *RLIM* cKO-Lip1: 307.2 ± 47.07 /mm^2^). One-way ANOVA with multiple comparisons; *, *p* < 0.05. (C) The bar graph shows the quantification of the ratio of KI67^+^PDGFRα^+^ among PDGFRα^+^ cells in (A) (control: 0.7525 ± 0.07472, *RLIM* cKO: 0.6910 ±0.07144, and *RLIM* cKO-Lip1: 0.7707 ± 0.08487). One-way ANOVA with multiple comparisons; *, *p* < 0.05; **, *p* < 0.01. (D) Representative images of immunofluorescence staining assays showing cleaved caspase 3 (CC3) and SOX10 expression in the corpus callosum of control (n = 3) and the *RLIM* cKO (n = 3) mice at P14. Scale bar, 50 μm. (E) The bar graph shows the quantification of the number of CC3^+^SOX10^+^ cells in (D) (control: 1.047 ± 0.09505 and *RLIM* cKO: 1.108 ± 0.02518). One-way ANOVA with multiple comparisons; n.s., not significant. (F) Western blot assays showing the expression of Caspase 3 and PAPR in OPCs purified from control or *RLIM* cKO mice. No activation (cleaved caspase 3 or PAPR) of caspase 3 or PAPR was observed. (G) The bar graph (mean ± SD) shows the normalized protein levels of RLIM (control: n = 3, 1.000 ± 0.1527; *RLIM* cKO: n = 3, 0.3420 ± 0.03093), Caspase 3 (control: n = 3, 1.000 ± 0.06003; *RLIM* cKO: n = 3, 1.041 ± 0.1172), and PAPR (control: n = 3, 1.000 ± 0.03044; *RLIM* cKO: n = 3, 1.068 ± 0.06620) with TUBULIN as an internal control. Unpaired two-tailed Students’ *t* test; ****, *p* < 0.0001; n.s., not significant. (H) Western blot assays showing the expression of GPX4, ACSL4, CHAC1 and RLIM in OPCs purified from control or *RLIM* cKO mice. (I) The bar graph (mean ± SD) shows the normalized protein levels of RLIM (control: n = 3, 1.000 ± 0.2024; *RLIM* cKO: n = 3, 0.2008 ± 0.1244), GPX4 (control: n = 3, 1.000 ± 0.1111; *RLIM* cKO: n = 3, 0.4842 ± 0.04265), ACSL4 (control: n = 3, 1.000 ± 0.08280; *RLIM* cKO: n = 3, 3.346 ± 0.3448), and CHAC1 (control: n = 3, 1.000 ± 0.05627; *RLIM* cKO: n = 3, 2.493 ± 0.1579) with TUBULIN as an internal control. Unpaired two-tailed Student’s *t* test. n.s., not significant. ****, *p* < 0.001.


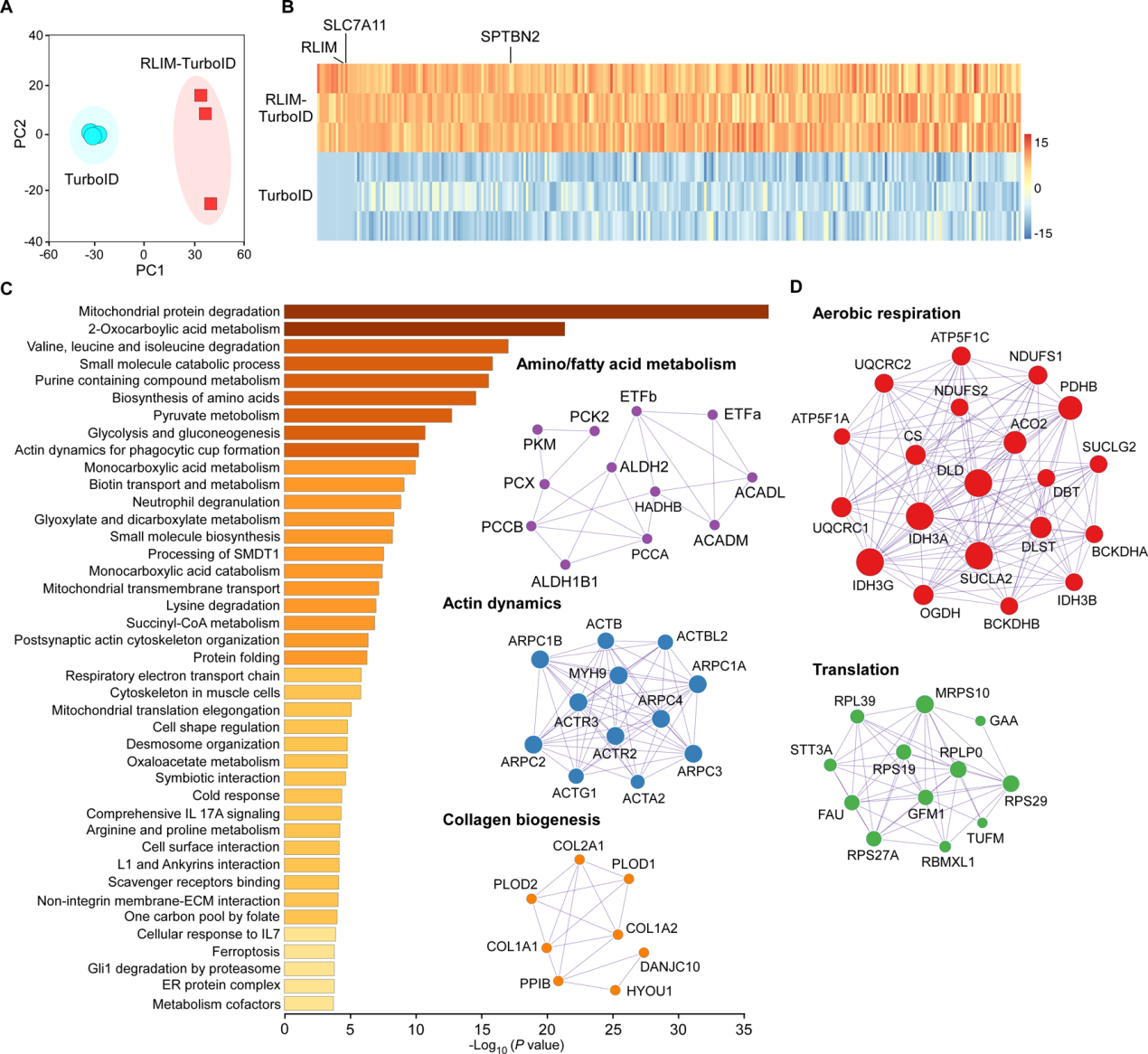


**Figure S9.** TurboID-based proximity labeling analysis of the protein interactome of RLIM in N2A cells. (A) The principal component analysis of RLIM-TurboID and TurboID proximal proteins with three biological replicates. (B) The proteins significantly enriched in RLIM-TurboID samples (n = 3) compared to TurboID samples (n = 3) with the statistics cut-offs of *p* < 0.01 and fold change ≥ 2. (C) GO enrichment analysis of the enriched protein identified in (A). (D) STRING analysis and functional clustering of the RLIM proximal proteins indenfied in (A).

**
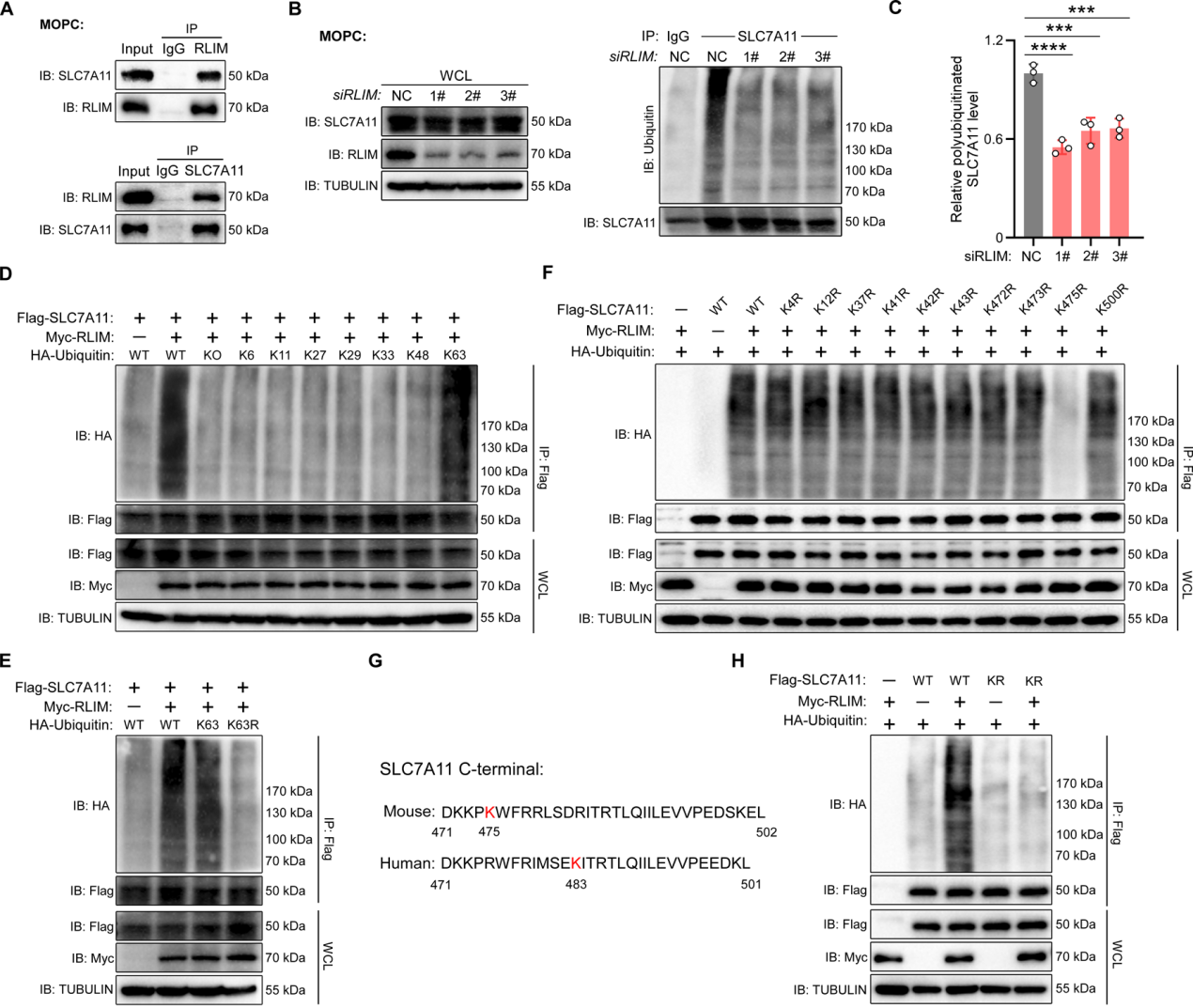
**

**Figure S10.** RLIM targets SLC7A11 for K63-linked polyubiquitination. (A) *In vivo* co-IP analysis of the interaction between endogenous RLIM and SLC7A11 in MOPC cells. The whole cell lysates were immunoprecipitated by IgG or RLIM (the upper panels) / SLC7A11 (the lower panels) antibody, and the inputs and IPs were immunoblotted by the indicated antibodies. (B) Ubiquitination analysis of the endogenous SLC7A11 polyubiquitination in MOPC cells transfected with control or *RLIM* siRNAs as indicated. (C) The bar graph (mean ± SD) shows the relative level of polyubiquitinated SLC7A11 in MOPC cells transfected with control of *RLIM* siRNAs as indicated (siNC, n = 3, 1.000 ± 0.05555; *siRLIM-1#*: n = 3, 0.5510 ± 0.04271; *siRLIM-2#*: n = 3, 0.6507 ± 0.07967; and *siRLIM-3#*: n = 3, 0.6656 ± 0.05926). The level of polyubiquitinated SLC7A11 in control MOPC cells was set to 1. One-way ANOVA with multiple comparisons; ***, *p* < 0.001; ****, *p* < 0.0001 (D, E) Ubiquitination analysis of RLIM-mediated polyubiquitination of SCL7A11 in the presence of wild-type or the indicated ubiquitin mutants in HEK293 cells. (F) Ubiquitination analysis of RLIM-mediated polyubiquitination of SLC7A11 mutants with the indicated lysines in N- or C-terminal regions of SLC7A11 mutated into arginines individually in HEK293 cells. (G) The C terminal amino acids of mouse and human SLC7A11. (H) Ubiquitination analysis of RLIM-mediated polyubiquitination of human SCL7A11 wild-type or K483R mutant in HEK293 cells. IB, immunoblotting; IP, immunoprecipitation; WT, wild-type; WCL, whole cell lysate; IP, immunoprecipitation.


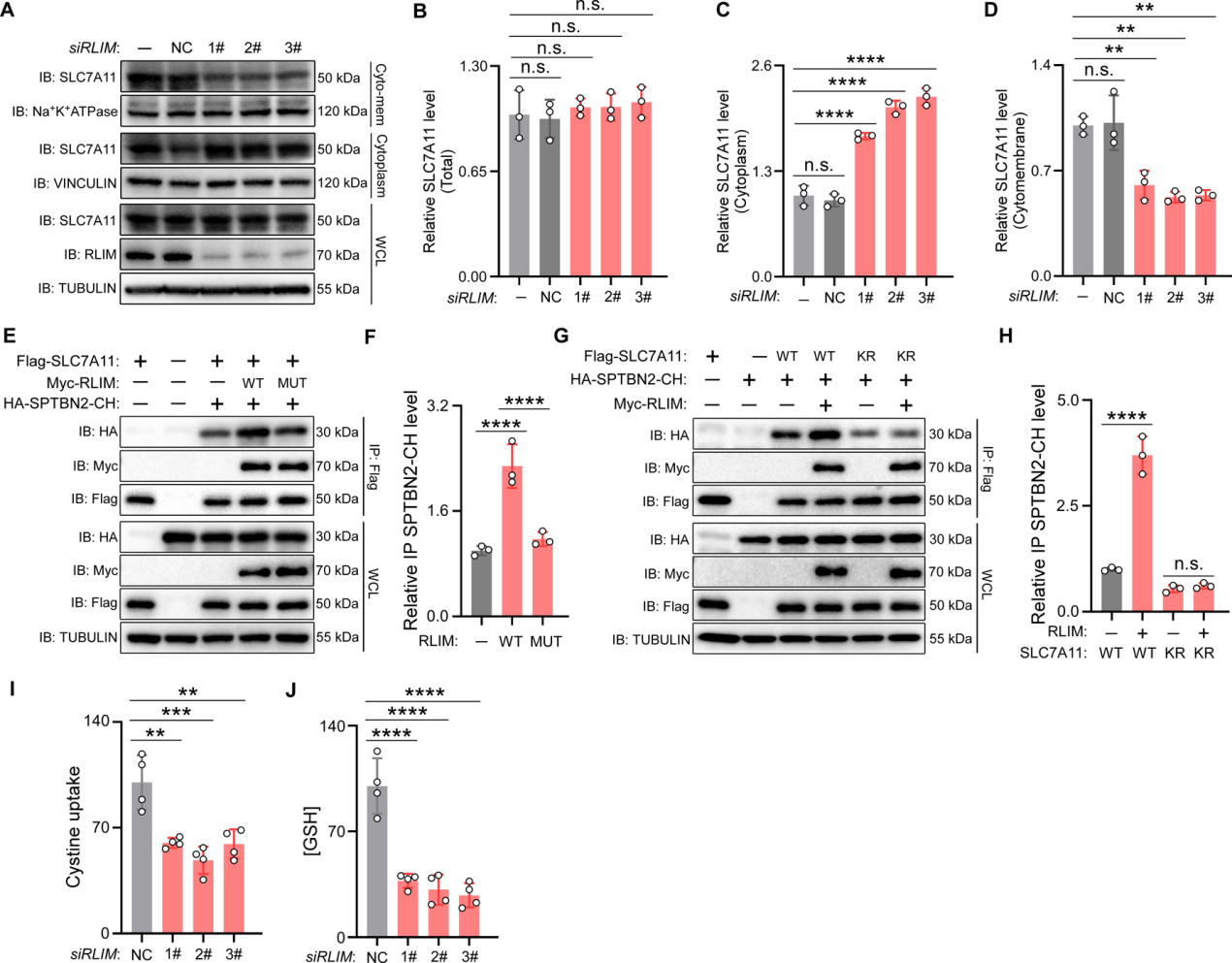


**Figure S11.** RLIM-mediated SLC7A11 polyubiquitinaiton is required for its membrane localization. (A) Western blot assays showing the subcellular distribution of endogenous SLC7A11 in MOPC cells transfected with control or *RLIM* siRNAs. (B) The bar graph (mean ± SD) shows the normalized protein levels of SLC7A11 in total cell lysates with TUBULIN as an internal control (control: n = 3, 1.000 ± 0.1509; siNC: n = 3, 0.9743 ± 0.1179; *siRLIM-1#*: n = 3, 1.046 ± 0.05423; *siRLIM-2#*: n = 3, 1.048 ± 0.08360; and *siRLIM-3#*: n = 3, 1.078 ± 0.09440). One-way ANOVA with multiple comparisons; n.s., not significant. (C) The bar graph (mean ± SD) shows the normalized protein levels of SLC7A11 in cytoplasmic fractions with VINCULIN as an internal control (control: n = 3, 1.000 ± 0.1203; siNC: n = 3, 0.9412 ± 0.07786; *siRLIM-1#*: n = 3, 1.732 ± 0.04038; *siRLIM-2#*: n = 3, 2.087 ± 0.08005; and *siRLIM-3#*: n = 3, 2.217 ± 0.1067). One-way ANOVA with multiple comparisons; n.s., not significant; ****, *p* < 0.0001. (D) The bar graph (mean ± SD) shows the normalized protein levels of SLC7A11 in cytomembrane fractions with Na^+^K^+^ATPase as an internal control (control: n = 3, 1.000 ± 0.06041; siNC: n = 3, 1.018 ± 0.1813; *siRLIM-1#*: n = 3, 0.6043 ± 0.09483; *siRLIM-2#*: n = 3, 0.5246 ± 0.03602; and *siRLIM-3#*: n = 3, 0.5364 ± 0.03441). One-way ANOVA with multiple comparisons; n.s., not significant; **, *p* < 0.01. (E) co-IP analysis of the interaction between SPTBN2-CH domain and SLC7A11 in HEK293 cells transfected with wild-type RLIM or its ligase dead mutant. HEK293 cells were transfected with the indicated combination of Flag-tagged SLC7A11, myc-tagged RLIM, or HA-tagged SPTBN2-CH and were harvested 48 hours after transfection for co-IP analysis. The inputs and IPs were immunoblotted by the indicated antibodies. (F) Bar graphs (mean ± SD) shows the normalized protein levels of immunoprecipitated SPTBN2-CH by SLC7A11 in (E) (Ctrl: 1.000 ± 0.07208, RLIM-WT: 2.283 ± 0.3330, RLIM-MUT: 1.175 ± 0.1053). One-way ANOVA with multiple comparisons. The protein level of immunoprecipiated SPTBN2 in control group was set to 1. One-way ANOVA with multiple comparisons; n.s., not significant; ****, *p* < 0.0001. (G) co-IP analysis of the interaction between SPTBN2-CH domain and wild-type or KR mutated SLC7A11 in HEK293 cells when RLIM is present or not. HEK293 cells were transfected with the indicated combination of Flag-tagged SLC7A11, myc-tagged RLIM, or HA-tagged SPTBN2-CH and were harvested 48 hours after transfection for co-IP analysis. The inputs and IPs were immunoblotted by the indicated antibodies. (H) Bar graphs (mean ± SD) shows the normalized protein levels of immunoprecipitated SPTBN2-CH by SLC7A11 in (G) (Ctrl-SLC7A11 WT: 1.000 ± 0.04300, RLIM-SLC7A1 WT: 3.695 ± 0.4452, Ctrl-SLC7A11 KR: 0.5412 ± 0.08611, RLIM-SLC7A11 KR: 0.6121 ± 0.06583). One-way ANOVA with multiple comparisons; n.s., not significant; ****, *p* < 0.0001. The protein level of immunoprecipiated SPTBN2 in Ctrl-SLC7A11 control group was set to 1. (I) The bar graph showing the relative levels of cystine uptaked by MOPC cells transfected with control or *RLIM* siRNAs (siNC: n = 4, 1.000 ± 0.1817; *siRLIM-1#*: n = 4, 0.5997 ± 0.03286; *siRLIM-2#*: n = 4, 0.4849 ± 0.09125; and *siRLIM-3#:* n = 4, 0.5921 ± 0.09668). The level of cystine uptaked by control cells was set to 1. One-way ANOVA with multiple comparisons; **, *p* < 0.01; ***, *p* < 0.001. (J) The bar graph showing intracellular GSH levels in MOPC cells transfected with control or *RLIM* siRNAs (siNC: n = 4, 1.000 ± 0.1845; *siRLIM-1#*: n = 4, 0.3733 ± 0.04772; *siRLIM-2#*: n = 4, 0.3154 ± 0.09968; and *siRLIM-3#*: n = 4, 0.2768 ± 0.08064). The GSH level in the control group was set to 1. One-way ANOVA with multiple comparisons; ****, *p* < 0.0001. IB, immunoblotting; WCL, whole cell lysate; KR, K475R; MUT, ligase dead mutant; IP, immunoprecipitation; WT, wild-type; NC, negative control.


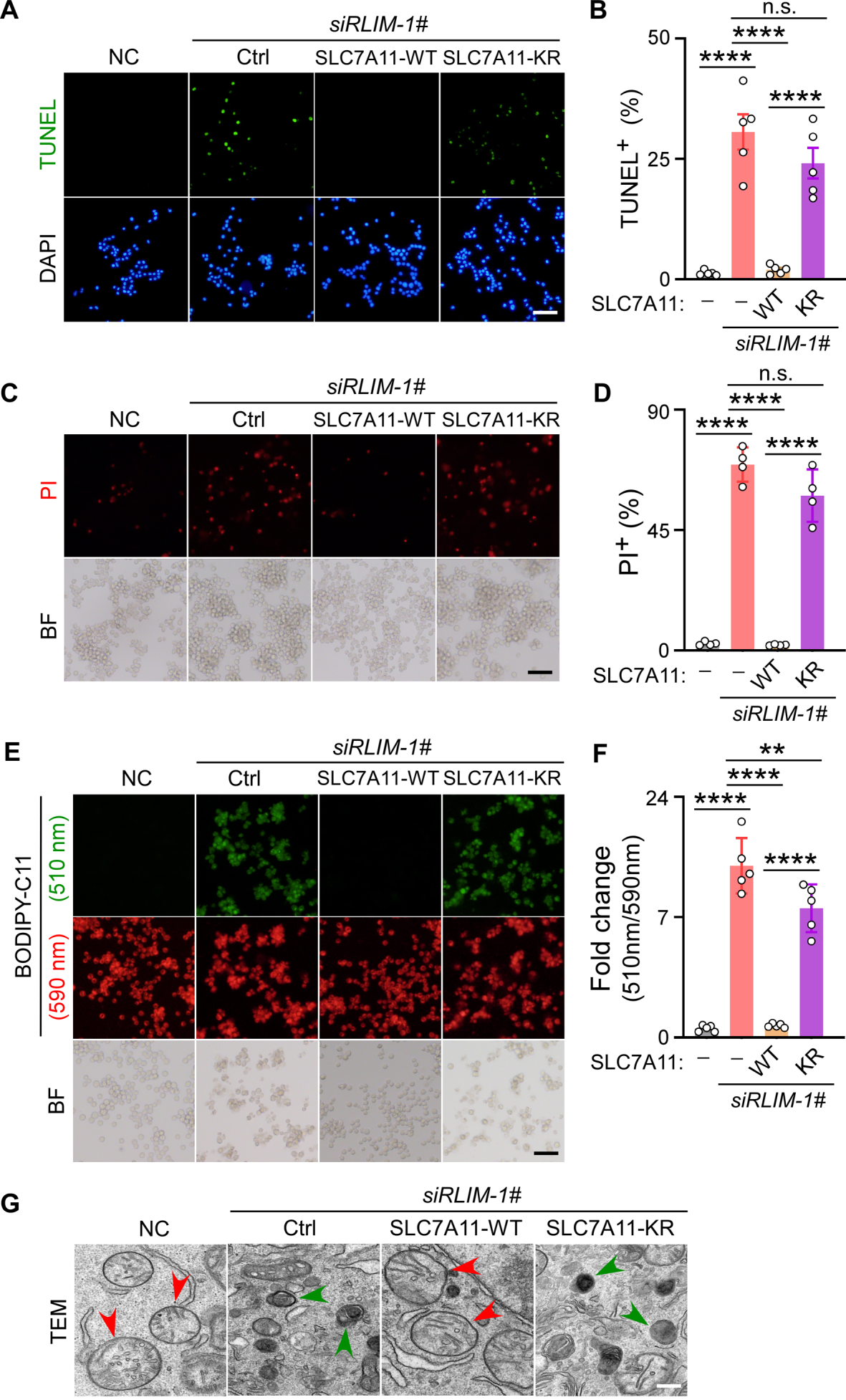


**Figure S12.** Expression of SLC7A11 wild-type, but not its KR mutant, resuced RLIM kockdown induced ferroptosis in MOPC cells. (A) The representative images of TUNEL staining for the indicated MOPC cells overexpressed with SLC7A11 wild-type or its KR mutant. Scale bar, 250 μm. (B) The bar graph shows the quantification of the percentage of TUNEL^+^ cells in each group in (A) (NC: n = 5, 1.276 ± 0.5174 %; *RLIM* siRNA-1#: n = 5, 30.60 ± 8.205 %; *RLIM* siRNA-1#-SLC7A11 WT: n = 5, 1.998 ± 0.9132 %; *RLIM* siRNA-1#-SLC7A11 KR: n = 5, 24.12 ± 7.121 %). One-way ANOVA with multiple comparisons; n.s., not significant; ****, *p* < 0.0001. (C) The representative images of PI staining for the indicated MOPC cells overexpressed with SLC7A11 wild-type or its KR mutant. Scale bar, 250 μm. (D) The bar graph shows the quantification of the percentage of PI^+^ cells in each group in (C) (NC: n = 4, 2.578 ± 0.7258 %; *RLIM* siRNA-1#: n = 4, 69.51 ± 6.420 %; *RLIM* siRNA-1#-SLC7A11 WT: n = 4, 2.078 ± 0.2235 %; *RLIM* siRNA-1#-SLC7A11 KR: n = 4, 57.88 ± 9.807 %). One-way ANOVA with multiple comparisons; n.s., not significant; ****, *p* < 0.0001. (E) The representative images show lipid ROS levels measured by the BODIPY-C11 probe in the indicated MOPC cells overexpressed with SLC7A11 wildtype or its KR mutant. Scale bar, 250 μm. (F) The bar graph shows the quantification of lipid ROS levels (BODIPY-C11_510 nm_^+^ cells %/BODIPY-C11_590 nm_^+^ cells %) in each group in (E) (NC: n = 5, 1.000 ± 0.2038; *RLIM* siRNA-1#: n = 5, 17.14 ± 2.759; *RLIM* siRNA-1#-SLC7A11 WT: n = 5, 1.186 ± 0.1964; *RLIM* siRNA-1#-SLC7A11 KR: n = 5, 12.89 ± 2.387). The lipid ROS level of the control group was set to 1. One-way ANOVA with multiple comparisons; **, *p* < 0.01; ****; *p* < 0.0001. (G) The indicated MOPC cells overexpressed with RLIM wild-type or its ligase dead mutant were subjected to transmission electron microscopy. Red arrows indicated mitochondria with obvious cristae, while green arrows represent shrunken mitochondria. Scale bar, 280 nm. WT, wild-type; Ctrl, control; KR, K475R; NC, negative control; BF, bright field.


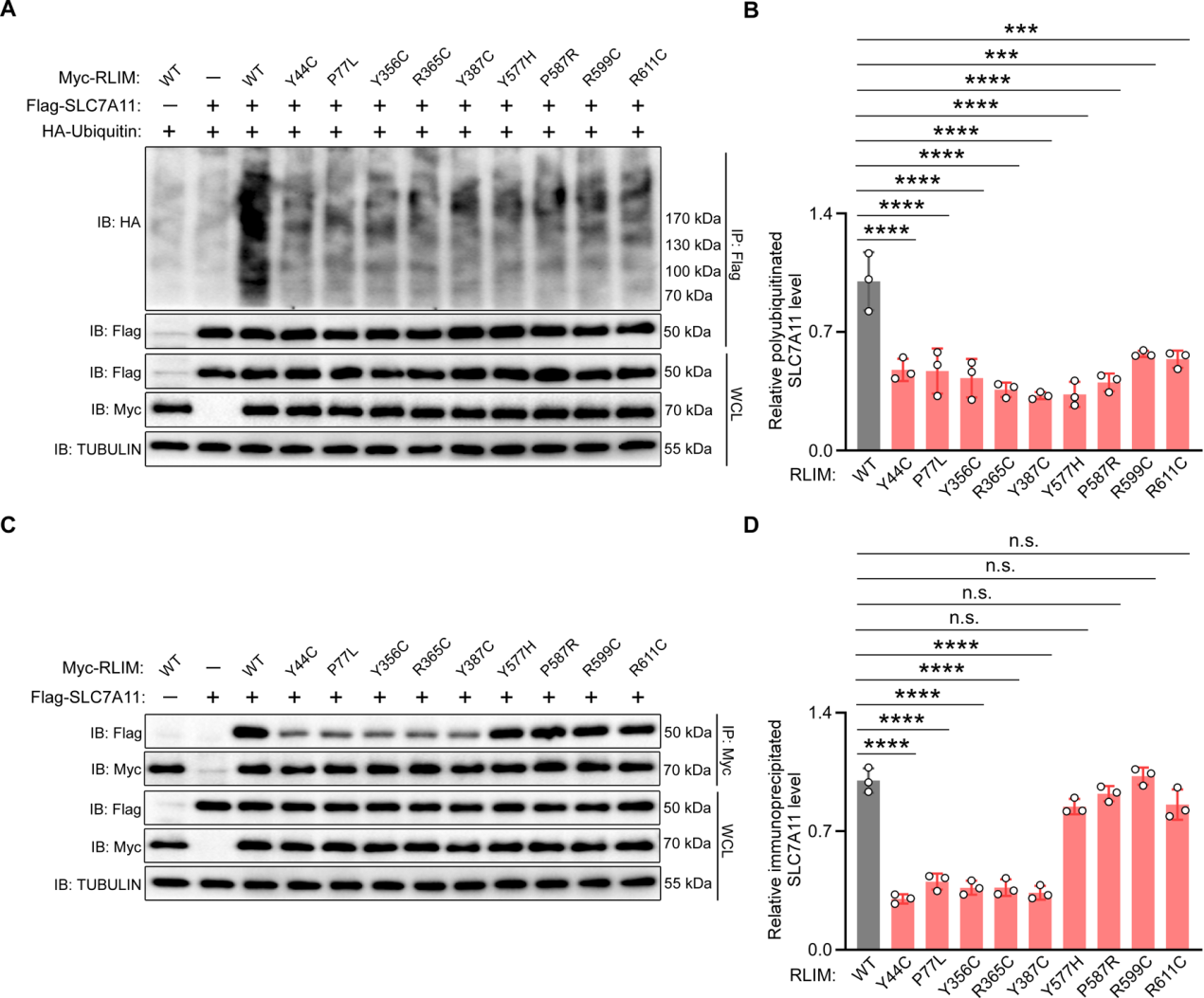


**Figure S13.** Regulation of SLC7A11 by RLIM is impaired by its human pathogenic mutations. (A) Polyubiquitination analysis of SLC7A11 mediated by wild-type or the indicated human pathogenic mutated RLIM in HEK293 cells. (B) The bar graph (mean ± SD) shows relative polyubiquitinated SLC7A11 levels (n = 3 for each group). The ubiquitination level of SLC7A11 in cells transfected with wild-type RLIM was set to 1 (RLIM-WT: 1.000 ± 0.1711, RLIM-Y44C: 0.4765 ± 0.06592, RLIM-P77L: 0.4695 ± 0.1333, RLIM-Y356C: 0.4275 ± 0.1138, RLIM-R365C: 0.3590 ± 0.04280, RLIM-Y387C: 0.3242 ± 0.02119, RLIM-Y577H: 0.3315 ± 0.07434, RLIM-P587R: 0.4028 ± 0.05224, RLIM-R599C: 0.5711 ± 0.01797, and RLIM-R611C: 0.5394 ± 0.05057). One-way ANOVA with multiple comparisons; ***; *p* < 0.001; ****, *p* < 0.0001. (C) Coimmunoprecipitation analysis of SLC7A11 interaction with wild-type or the indicated human pathogenic mutated RLIM in HEK293 cells. (D) The bar graph (mean ± SD) shows relative coimmunoprecipitated SLC7A11 levels (n = 3 for each group). The coimmunoprecipitated SLC7A11 level in cells transfected with wild type RLIM was set to 1 (RLIM-WT: 1.000 ± 0.07240, RLIM-Y44C: 0.3006 ± 0.02758, RLIM-P77L: 0.4028 ± 0.04752, RLIM-Y356C: 0.3667 ± 0.04158, RLIM-R365C: 0.3677 ± 0.04926, RLIM-Y387C: 0.3370 ± 0.04072, RLIM-Y577H: 0.8462 ± 0.04589, RLIM-P587R: 0.9222 ± 0.04485, RLIM-R599C: 1.026 ± 0.05069, and RLIM-R611C: 0.8576 ± 0.08984). One-way ANOVA with multiple comparisons; n.s., not significant; ****, *p* < 0.0001. IB, immuoblotting; IP, immunoprecipitation; WCL, whole cell lysate; WT, wild type.


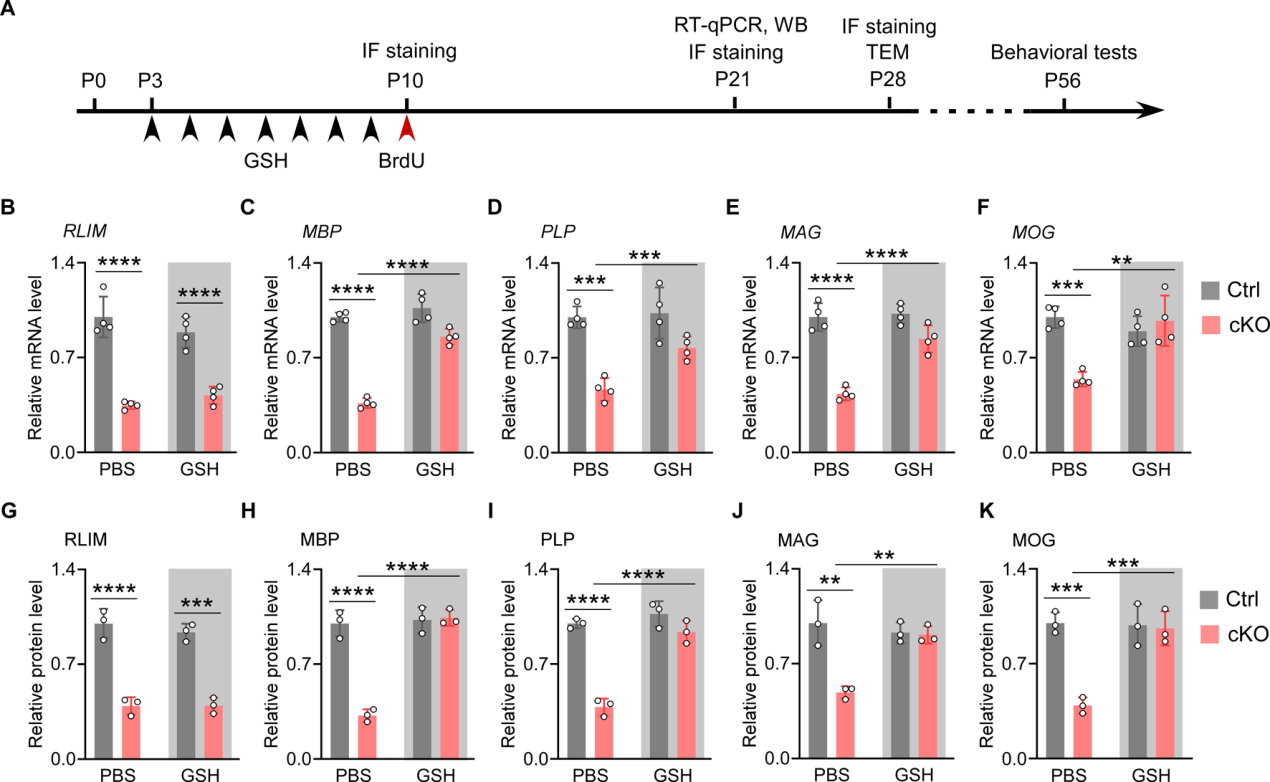


**Figure S14.** Neonatal GSH administration restores the defects in OL lineage development and myelination in the *RLIM* cKO mice. (A) Schematic diagram of GSH administration and the indicated analysis for control and the *RLIM* cKO mice. (B-F) Bar graphs showing the results of real time RT-PCR analysis of the expression levels of *RLIM* (B) (control-PBS: n = 4, 1.000 ± 0.1505; *RLIM* cKO-PBS: n = 4, 0.3475 ± 0.02807; control-GSH: n = 4, 0.8867 ± 0.1144; and *RLIM* cKO-GSH: n = 4, 0.4210 ± 0.06466) and myelin related genes, including *MBP* (C) (control-PBS: n = 4, 1.000 ± 0.03409; *RLIM* cKO-PBS: n = 4, 0.3654 ± 0.03677; control-GSH: n = 4, 1.068 ± 0.1041; and *RLIM* cKO-GSH: n = 4, 0.8562 ± 0.05727), *PLP* (D) (control-PBS: n = 4, 1.000 ± 0.08023; *RLIM* cKO-PBS: n = 4, 0.4674 ± 0.08414; control-GSH: n = 4, 1.030 ± 0.1898; and *RLIM* cKO-GSH: n = 4, 0.7740 ± 0.08510), *MAG* (E) (control-PBS: n = 4, 1.000 ± 0.1028; *RLIM* cKO-PBS: n = 4, 0.4336 ± 0.04861; control-GSH: n = 4, 1.024 ± 0.07215; and *RLIM* cKO-GSH: n = 4, 0.8388 ± 0.1008), and *MOG* (F) (control-PBS: n = 4, 1.000 ± 0.07877; *RLIM* cKO-PBS: n = 4, 0.5422 ± 0.05388; control-GSH: n = 4, 8964 ± 0.1109; and *RLIM* cKO-GSH: n = 4, 0.9734 ± 0.1859) in the cortical tissues of control and the *RLIM* cKO mice administrated with GSH or not at P21. *β-ACTIN* was used as an internal control. The expression level of the indicated gene in the control group was set to 1. One-way ANOVA with multiple comparisons; **, *p* < 0.01; ***, *p* < 0.001; ****, *p* < 0.0001. (G-K) Bar graphs showing the statistics of western blot analysis of the protein levels of RLIM in Figure 6P (G) (control-PBS: n = 3, 1.00 ± 0.1090; *RLIM* cKO-PBS: n = 3, 0.3921 ± 0.06444; control-GSH: n = 3, 0.9360 ± 0.06369; and *RLIM* cKO-GSH: n = 3, 0.3953 ± 0.05822) and myelin related proteins, including MBP (H) (control-PBS: n = 3, 1.00 ± 0.09844; *RLIM* cKO-PBS: n = 3, 0.3214 ± 0.04613; control-GSH: n = 3, 1.027 ± 0.09467; and *RLIM* cKO-GSH: n = 3, 1.043 ± 0.05895), PLP (I) (control-PBS: n = 3, 1.00 ± 0.03519; *RLIM* cKO-PBS: n = 3, 0.3826 ± 0.06245; control-GSH: n = 3, 1.070 ± 0.09401; and *RLIM* cKO-GSH: n = 3, 9370 ± 0.08405), MAG (J) (control-PBS: n = 3, 1.000 ± 0.1686; *RLIM* cKO-PBS: n = 3, 4885 ± 0.04559; control-GSH: n = 3, 9302 ± 0.07536; and *RLIM* cKO-GSH: n = 3, 0.9141 ± 0.06513), and MOG (K) (control-PBS: n = 3, 1.000 ± 0.07914; *RLIM* cKO-PBS: n = 3, 0.3920 ± 0.05976; control-GSH: n = 3, 0.9844 ± 0.1549; and *RLIM* cKO-GSH: n = 3, 0.9617 ± 0.1255) in the cortical tissues of control and the *RLIM* cKO mice administrated with GSH or not at P21. TUBULIN was used as an internal control. The indicated protein level in the control group was set to 1. One-way ANOVA with multiple comparisons; **, *p* < 0.01; ***, *p* < 0.001; ****, *p* < 0.0001. Ctrl, control; IF, immunofluorescence; WB, Western blot; TEM, transmission electron microscopy.


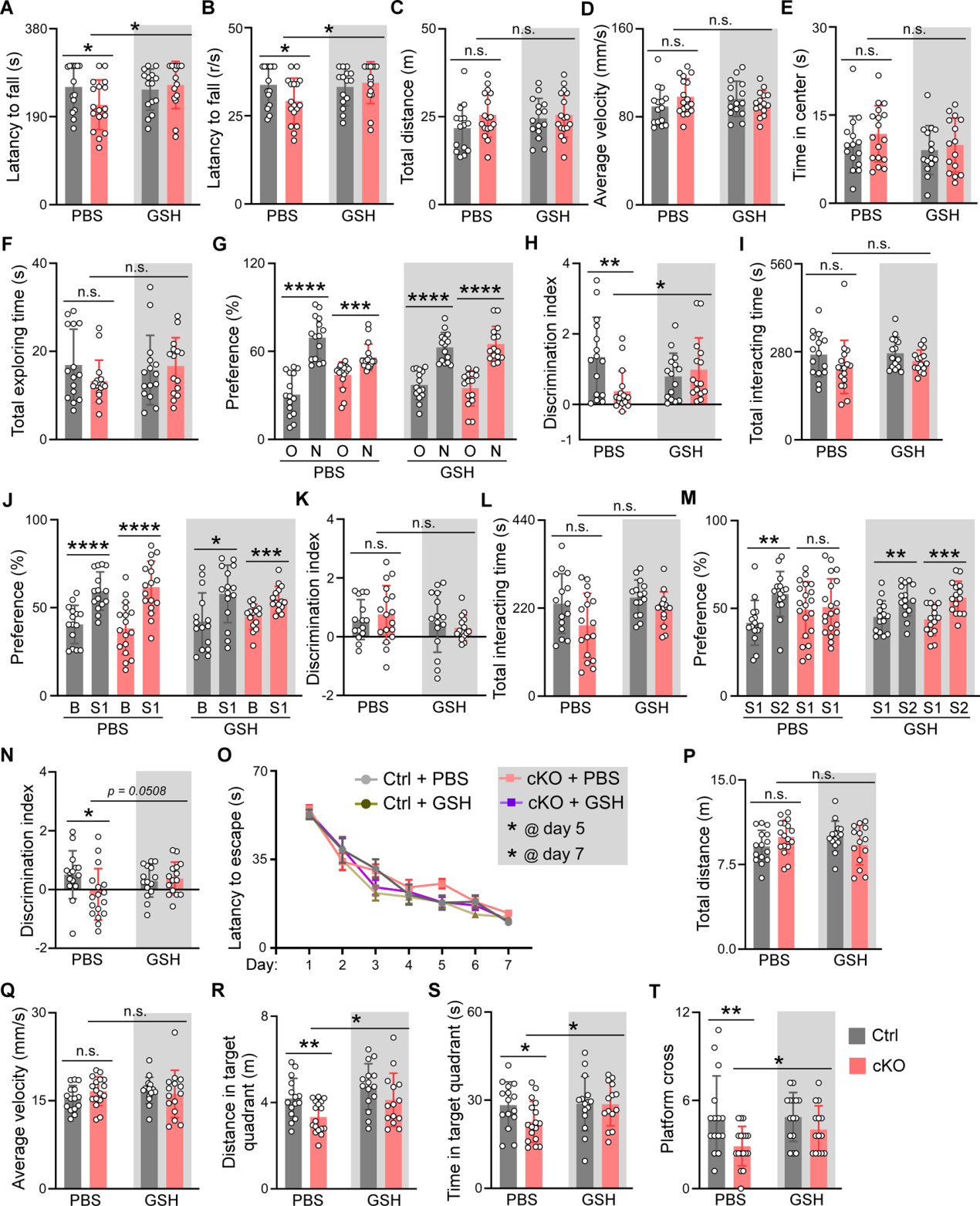


**Figure S15.** GSH administration reverses behavioral impairments in motor, social, learning and memory in the *RLIM* cKO mice. (A, B) Mouse performances in rotarod test (control-PBS, n = 15; *RLIM* cKO-PBS, n = 15; control-GSH, n = 17; and *RLIM* cKO-GSH, n = 15), including latent time (mean ± SD, control-PBS: 254.3 ± 49.68 s, *RLIM* cKO-PBS: 248.6 ± 44.32 s, control-GSH: 215.6 ± 53.75 s, and *RLIM* cKO-GSH: 258.4 ± 50.99 s) on a running rotarod (A) and the maximal rate (mean ± SD, control-PBS: 33.80 ± 5.722 r/s, *RLIM* cKO-PBS: 29.24 ± 6.447 r/s, control-GSH: 33.20 ± 5.308 r/s, and *RLIM* cKO-GSH: 34.40 ± 5.914 r/s) when mice fell on an accelerated rotarod (B). Unpaired two-tailed Student’s *t*-test. The comparison between Ctrl (n = 15 ) and cKO (n = 17) yielded P = 0.0442, Cohen's d = 0.76, post-hoc power = 0.75 (A) and P = 0.0437, Cohen's d = 0.87, post-hoc power = 0.82 (B), while the comparison between cKO (n = 17 ) and cKO-GSH (n = 15) yielded P = 0.008, Cohen's d = 1.00, post-hoc power = 0.735 (A) and P = 0.025, Cohen's d = 0.832, post-hoc power = 0.506 (B). (C-E) Mouse performances in open field test (control-PBS: n = 15, *RLIM* cKO-PBS: n = 17, control-GSH: n = 15, and *RLIM* cKO-GSH, n = 17), including total moving distance (C) (mean ± SD, control-PBS: 21,781 ± 6,734 mm, *RLIM* cKO-PBS: 25,519 ± 6,355 mm, control-GSH: 24,541 ± 5,700 mm, and *RLIM* cKO-GSH: 25,519 ± 6,355 mm), average velocity (D) (mean ± SD, control-PBS: 89.46 ± 17.18 mm/s, *RLIM* cKO-PBS: 98.29 ± 16.38 mm/s, control-GSH: 96.48 ± 15.99 mm/s, and *RLIM* cKO-GSH: 92.44 ± 11.27 mm/s), and time in center (E) (mean ± SD, control-PBS: 10.03 ± 4.818 s, *RLIM* cKO-PBS: 11.83 ± 4.789 s, control-GSH: 9.053 ± 4.155 s, and *RLIM* cKO-GSH: 9.915 ± 4.579 s). One-way ANOVA with multiple comparisons; *, *p* < 0.05; n.s., not significant. (F-H) Mouse performance in the novel object recognition test (control-PBS: n = 15, *RLIM* cKO-PBS: n = 17, control-GSH: n = 15, and *RLIM* cKO-GSH, n = 15). (F) The bar graph (mean ± SD) shows the total exploring time (control-PBS: 16.93 ± 8.102 s, *RLIM* cKO-PBS: 13.28 ± 4.733 s, control-GSH: 15.76 ± 7.866 s, and *RLIM* cKO-GSH: 16.68 ± 6.436 s) close to new (N) and old (O) objects. (G) The bar graph (mean ± SD) shows the percentage of exploring time close to the old and new objects (control-PBS: old, 30.59 ± 14.36 %; new, 69.41 ± 14.36 %; *RLIM* cKO-PBS: old, 43.95 ± 8.955 %; new, 56.05 ± 8.955 %; control-GSH: old, 37.23 ± 10.01 %; new, 62.77 ± 10.01 %; and *RLIM* cKO-GSH: old, 34.96 ± 11.96 %; new, 65.04 ± 11.96 %). (H) The bar graph (mean ± SD) shows the logarithm of discrimination index (control-PBS: 1.345 ± 1.126, *RLIM* cKO-PBS: 0.3733 ± 0.5748, control-GSH: 0.7935 ± 0.6603, and *RLIM* cKO-GSH: 0.9880 ± 0.9012). One-way ANOVA with multiple comparisons; n.s., not significant; *, *p* < 0.05; **, *p* < 0.01; ***, *p* < 0.001; ****, *p* < 0.0001. (I-N) Mouse performances in the three-chamber social test (control-PBS: n = 15, *RLIM* cKO-PBS: n = 17, control-GSH: n = 15, and *RLIM* cKO-GSH, n = 15). (I) The bar graph (mean ± SD) shows the total interacting time close to the inanimate ball and stranger mouse (control-PBS: 271.6 ± 70.54 s, *RLIM* cKO-PBS: 232.0 ± 84.57 s, control-GSH: 275.6 ± 50.75 s, and *RLIM* cKO-GSH: 250.8 ± 35.16 s). (J) The bar graph (mean ± SD) shows the percentage of interacting time to the inanimate ball and stranger mouse (control-PBS: ball, 40.53 ± 10.88 % and stranger, 59.47 ± 10.88 %; *RLIM* cKO-PBS: ball, 38.31 ± 14.74 % and stranger, 61.69 ± 14.74 %; control-GSH: ball, 42.13 ± 16.34 % and stranger, 57.87 ± 16.34 %; and *RLIM* cKO-GSH: ball, 44.98 ± 7.088 % and stranger, 55.02 ± 7.088 %). (K) The bar graph (mean ± SD) shows the logarithm of social ratio in (J) (control-PBS: 0.5832 ± 0.6788, *RLIM* cKO-PBS: 0.7598 ± 0.9752, control-GSH: 0.4921 ± 1.022, and *RLIM* cKO-GSH: 0.2987 ±0.4272). (L) The bar graph (mean ± SD) shows the total interacting time close to the familiar and stranger mice (control-PBS: 231.6 ± 74.72 s, *RLIM* cKO-PBS: 177.3 ± 79.89 s, control-GSH: 246.7 ± 43.58 s, and *RLIM* cKO-GSH: 218.1 ± 42.68 s). (M) The bar graph (mean ± SD) shows the percentage of interacting time to the familiar and stranger mouse (control-PBS: familiar, 41.77 ± 12.76 % and stranger, 58.23 ± 12.76 %; *RLIM* cKO-PBS: familiar, 49.19 ± 16.04 % and stranger, 50.81 ± 16.04 %; control-GSH: familiar, 45.13 ± 9.196 % and stranger, 54.87 ± 9.196 %; and *RLIM* cKO-GSH: familiar, 43.79 ± 9.235 %; stranger, 56.21 ± 9.235 %). (N) The bar graph (mean ± SD) shows the logarithm of social ratio in (M) (control-PBS: 0.5035 ± 0.8133, *RLIM* cKO-PBS: -0.1624 ±0.8743, control-GSH: 0.2883 ± 0.5455, and *RLIM* cKO-GSH: 0.3736 ± 0.5573). One-way ANOVA with multiple comparisons; n.s., not significant; *, *p* < 0.05; **, *p* < 0.01; ***, *p* < 0.001; ****, *p* < 0.0001. (O-T) Mouse performances in the Morris water maze test (control-PBS: n = 15, *RLIM* cKO-PBS: n = 17, control-GSH: n = 15, and *RLIM* cKO-GSH: n = 14). (O) Escape latencies (mean ± SEM) to find the platform throughout the 7-day learning trials (control-PBS: day 1, 52.80 ± 1.920 s; day 2, 38.78 ± 5.107 s; day 3, 31.29 ± 3.784 s; day 4, 21.13 ± 4.029 s; day 5, 17.91 ± 2.797 s; day 6, 18.31 ± 2.559 s; day 7, 10.13 ± 1.384 s; *RLIM* cKO-PBS: day 1, 54.94 ± 1.534 s; day 2, 34.22 ± 3.297 s; day 3, 30.57 ± 2.536 s; day 4, 24.00 ± 3.001 s; day 5, 25.41 ± 1.897 s; day 6, 18.27 ± 2.172 s; day 7, 13.75 ± 0.9925 s; control-GSH: day 1, 53.49 ± 1.960 s; day 2, 34.76 ± 4.041 s; day 3, 21.71 ± 2.934 s; day 4, 20.16 ± 2.675 s; day 5, 18.22 ± 2.611 s; day 6, 13.22 ± 1.401 s; day 7, 12.16 ± 2.009 s; *RLIM* cKO-GSH: day 1, 53.57 ± 1.851 s; day 2, 39.19 ± 4.221 s; day 3, 24.00 ± 2.929 s; day 4, 22.26 ± 2.065 s; day 5, 18.12 ± 2.102 s; day 6, 16.86 ± 1.858 s; day 7, 11.10 ± 0.5496 s). (P-T) Spatial memory retrieval of these mice used in (O) was examined when the platform was removed. (P) The bar graph (mean ± SD) shows the total moving distance during spatial memory tests (control-PBS: 9,099 ± 1,441 mm, *RLIM* cKO-PBS: 9,956 ± 1,476 mm, control-GSH: 10,009 ± 1,346 mm, and *RLIM* cKO-GSH: 9,234 ± 1,762 mm). (Q) The bar graph (mean ± SD) shows the average moving velocity during spatial memory tests (control-PBS: 154.3 ± 21.66 mm/s, *RLIM* cKO-PBS: 166.2 ± 24.60 mm/s, control-GSH: 167.7 ± 22.34 mm/s, and *RLIM* cKO-GSH: 161.8 ± 40.24 mm/s). (R) The bar graph (mean ± SD) shows the moving distance in the target quadrant during spatial memory tests (control-PBS: 4,192 ± 932.4 mm, *RLIM* cKO-PBS: 3,333 ± 727.2 cm, control-GSH: 4,739 ± 1.060 cm, and *RLIM* cKO-GSH: 4,125 ± 1,234 mm). (S) The bar graph (mean ± SD) shows the duration in the target quadrant during spatial memory tests (control-PBS: 28.33 ± 7.879 s, *RLIM* cKO-PBS: 22.64 ± 6.966 s， control-GSH: 28.84 ± 9.278 s, and *RLIM* cKO-GSH: 28.62 ± 7.332 s). (T) The bar graph (mean ± SD) shows the number of target crosses during spatial memory tests (control-PBS: 4.133 ± 2.264, *RLIM* cKO-PBS: 2.412 ± 1.121, control-GSH: 4.067 ± 1.387, and *RLIM* cKO-GSH: 3.357 ± 1.336). One-way ANOVA with multiple comparisons; n.s., not significant; *, *p* < 0.05; **, *p* < 0.01; ***, *p* < 0.001; ****, *p* < 0.0001. Ctrl, control; cKO, *RLIM* cKO; N: the new object; O, the old object; B, ball; S1, stranger 1 or the familiar; S2, stranger 2 or the stranger.


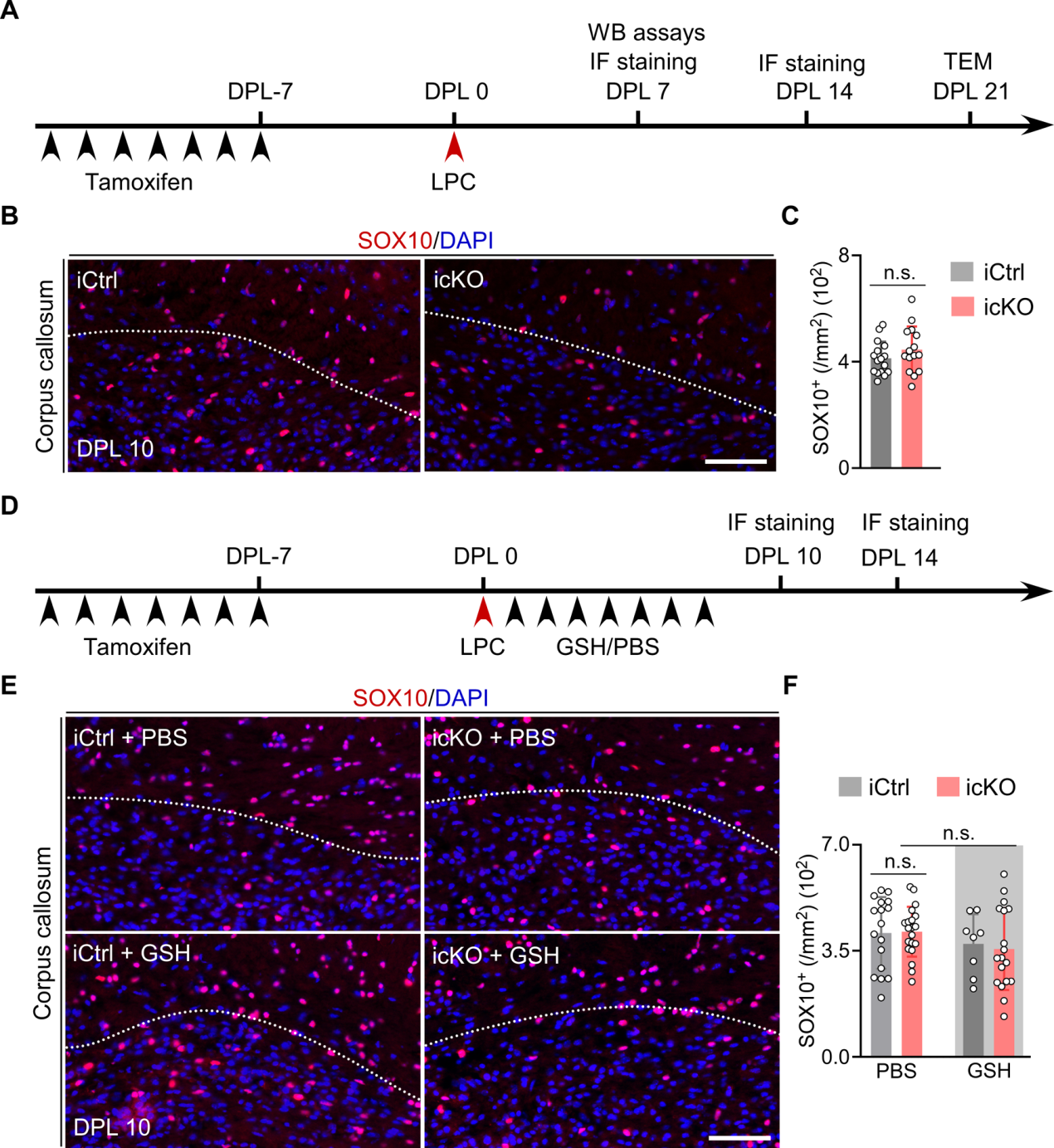


**Figure S16.** RLIM is required for remyelination and GSH administration rescues the defects in remyelination in adult *RLIM* cKO mice. (A) Schematic diagram of L-α-lysophosphatidycholine (LPC) induced lesioning in the corpus callosum regions of icontrol and the *RLIM* icKO mice and the indicated analysis at the indicated stages. (B, C) Immunofluorescence staining of SOX10 in lesion areas of brains from icontrol (n = 3) and the *RLIM* icKO (n = 3) mice at DPL 7. Bar graphs (C) showing the quantification of the number of SOX10^+^ cells in (B) (icontrol: 413.1 ± 62.57 /mm^2^ and *RLIM* icKO: 445.8 ± 87.26 /mm^2^). Unpaired two-tailed Student’s *t* test; n.s., not significant. (D) Schematic diagram of LPC induced lesioning in the corpus callosum regions of icontrol and the *RLIM* icKO mice, GSH administration, and the indicated analysis at the indicated stages. (E, F) Immunofluorescence staining of SOX10 in lesion areas of brains from icontrol-PBS (n = 3), *RLIM* icKO-PBS (n = 3), icontrol-GSH (n = 3), and *RLIM* icKO-GSH (n = 3) mice at DPL 10. Bar graphs (F) showing the quantification of the number of SOX10^+^ cells in (E) (icontrol-PBS: 409.3 ± 118.2 /mm^2^, *RLIM* icKO-PBS: 416.1 ± 82.17 /mm^2^, icontrol-GSH: 372.9 ± 97.86 /mm^2^, and *RLIM* icKO-GSH: 356.0 ± 135.0 /mm^2^). One-way ANOVA with multiple comparisons; n.s., not significant. DPL, day post lesion; IF, immunofluorescence; WB, Western blot; TEM, transmission electronic microscopy.

**Supplementary Table 1 (separate excel file), related to Figure 4. Differentially expressed genes (DEG) list identified by RNA-seq in purified PDGFRα^+^ OPC cells from cortical tissues of P7 Control and *RLIM* cKO mice.**

**Supplementary Table 2 (separate excel file), related to Figure 4. Lipidomics analysis showing the quantification of identified lipids in differernt cell samples.**

**Supplementary Table 3 (separate excel file), related to Figure 5. Potential RLIM interacting proteins identified by TurboID-based proximity labeling and mass spectrometry.**
