# Supplementary material for: The E3 Ubiquitin Ligase RLIM Safeguards Oligodendrocyte Development and Myelination by Targeting SLC7A11 for Polyubiquitination to Regulate Ferroptotic Resistance
Source: Adv Sci (Weinh). 2026 Jul 23:e76315. Online ahead of print. doi: 10.1002/advs.76315 (PMC13395749; doi:10.1002/advs.76315)

Figure 1I:

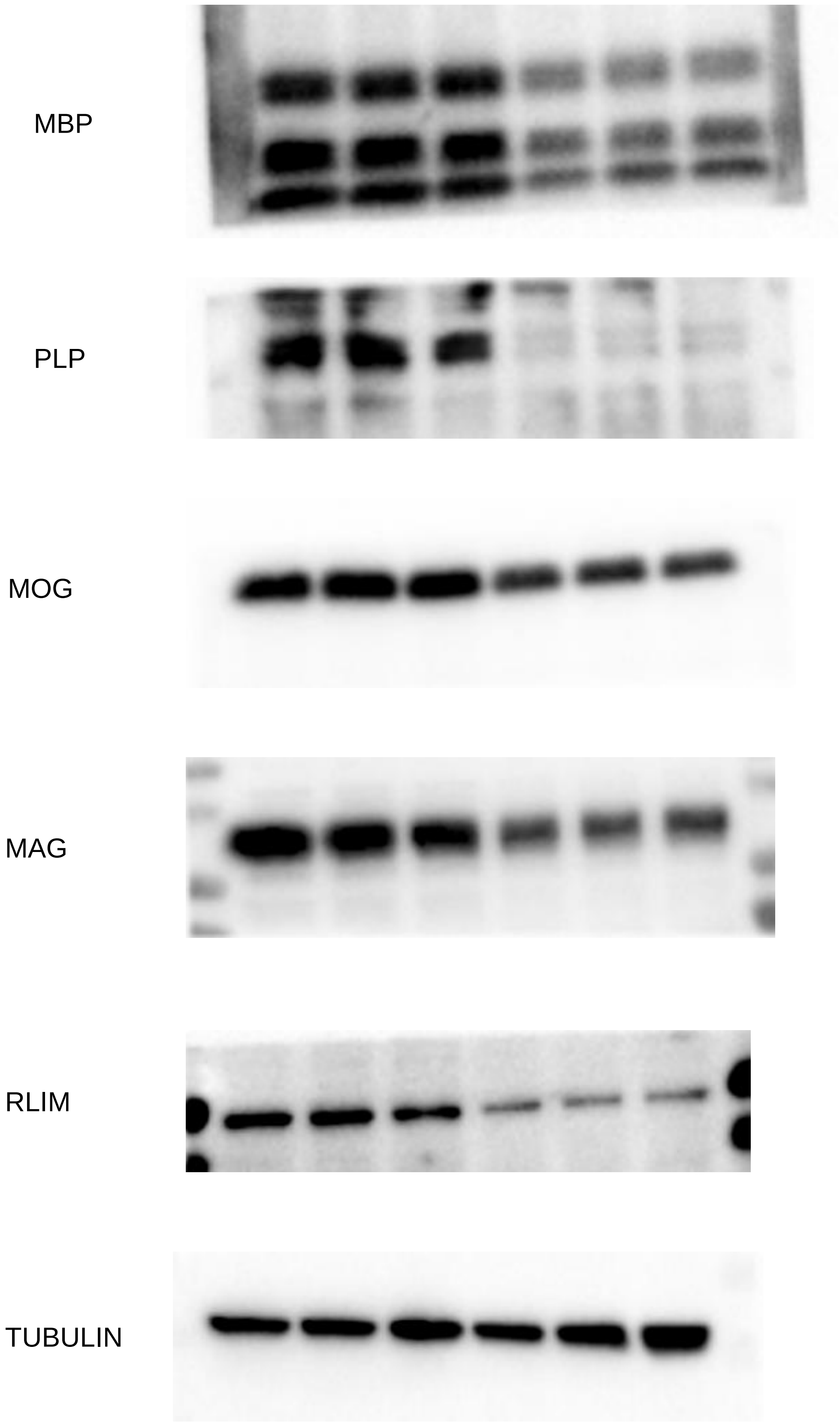

Figure 5A:

Upper panel: IP-HA-RLIM

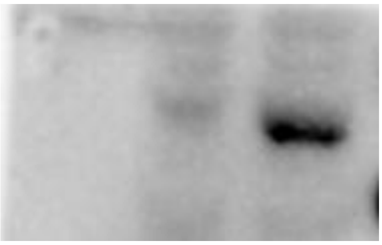

Upper panel: IP-Flag-SLC7A11

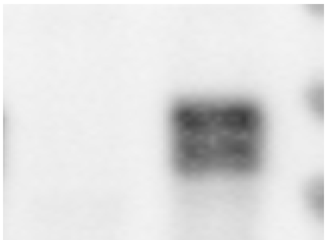

Upper panel: WCL-HA-RLIM

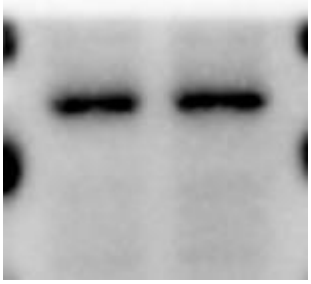

Upper panel: WCL-Flag-SLC7A11

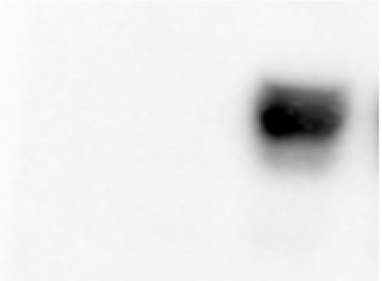

Lower panel: IP-Flag-SLC7A11

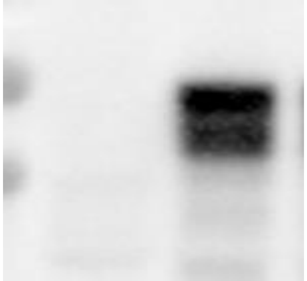

Lower panel: IP-HA-RLIM

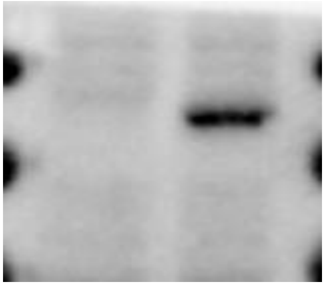

Lower panel: WCL-Flag-SLC7A11

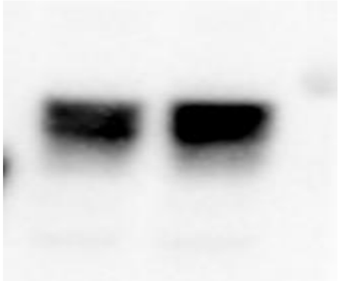

Lower panel: WCL-HA-RLIM

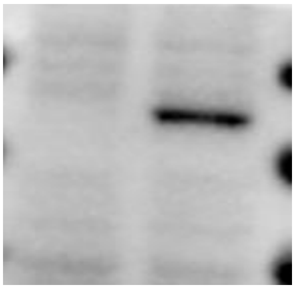

Figure 5B:

Upper panel: SLC7A11

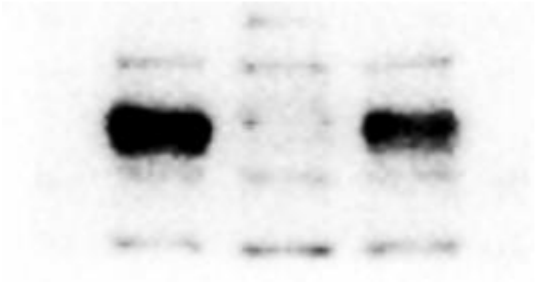

Upper panel: RLIM

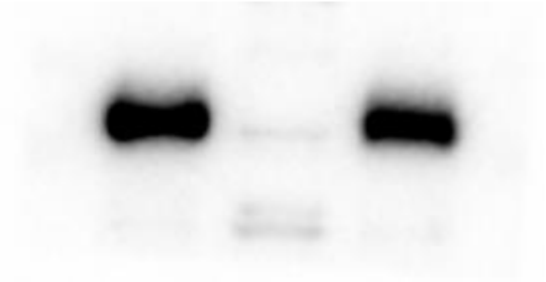

Lower panel: RLIM

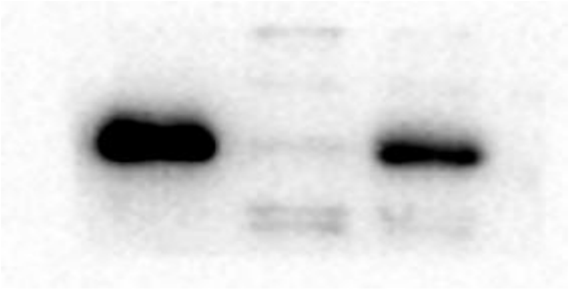

Lower panel: SLC7A11

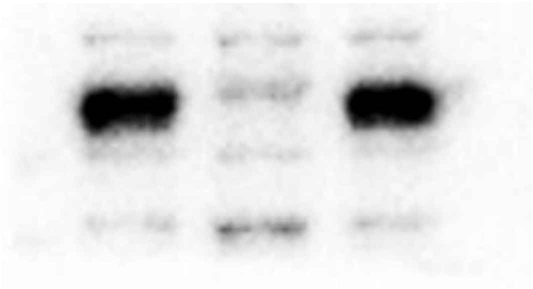

Figure 5C:

Input---SLC7A11

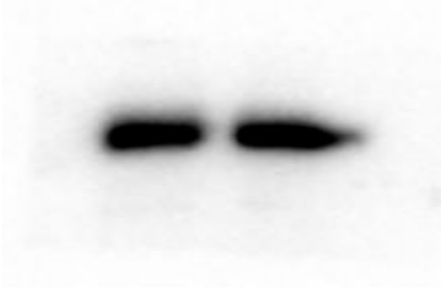

Pulldown---SLC7A11

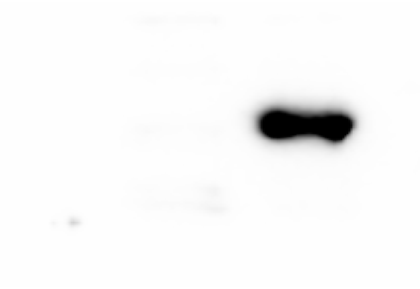

Input---RLIM

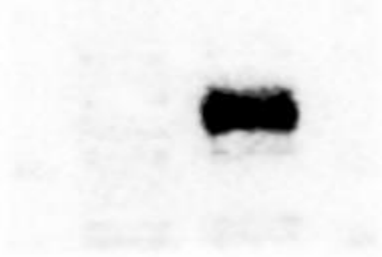

Pulldown---RLIM

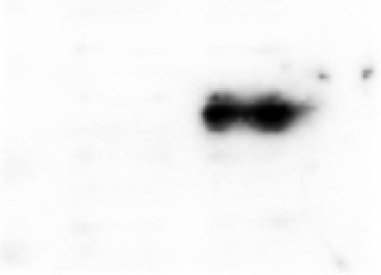

Figure 5D:

IP---HA

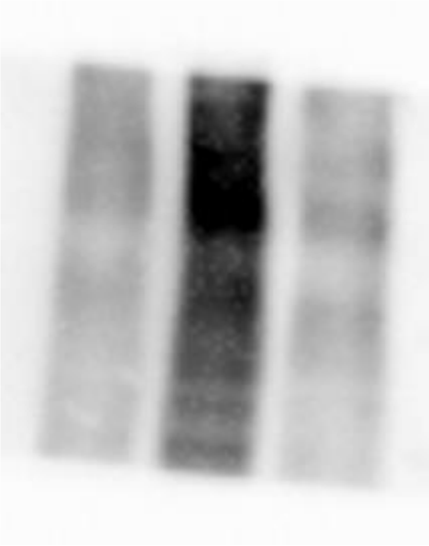

IP---Flag-SLC7A11

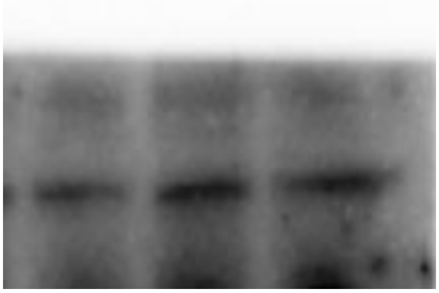

WCL---Flag-SLC7A11

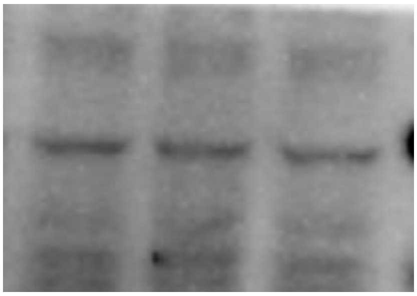

WCL---Myc-RLIM

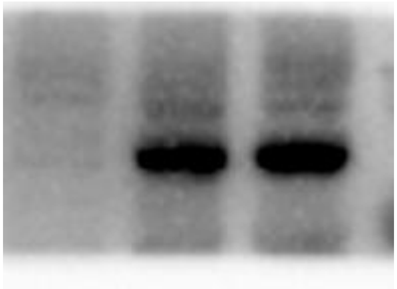

WCL---TUBULIN

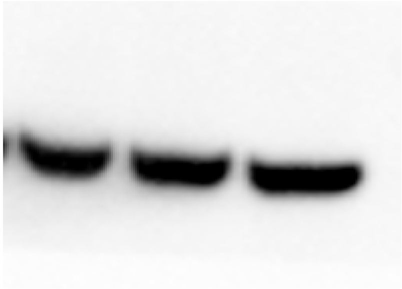

Figure 5E:

HA

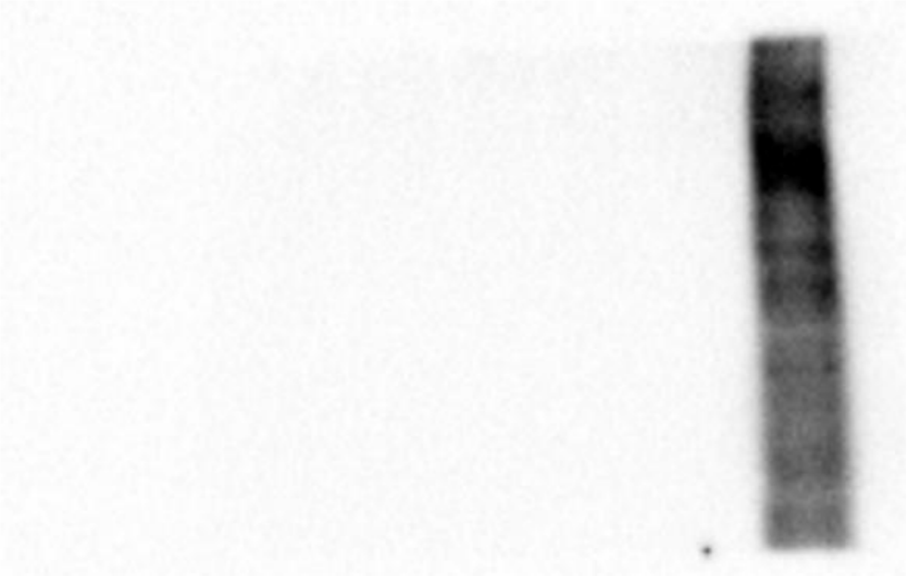

SLC7A11

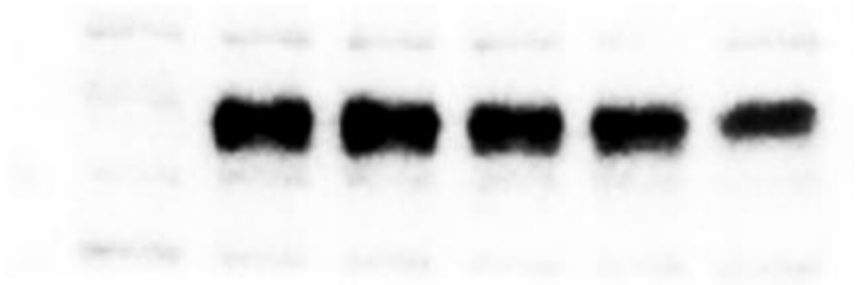

Figure 5F:

IP---Ubiquitin

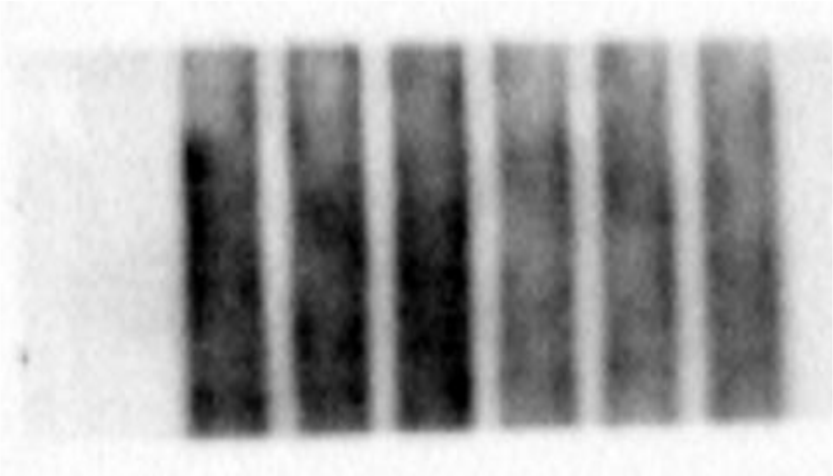

IP---SLC7A11

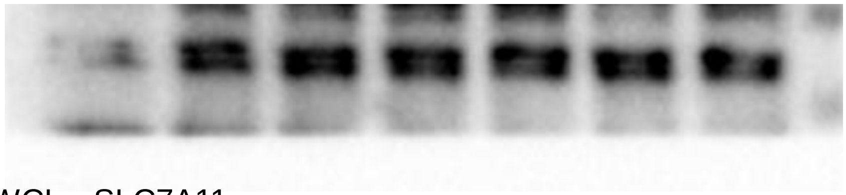

WCL---SLC7A11

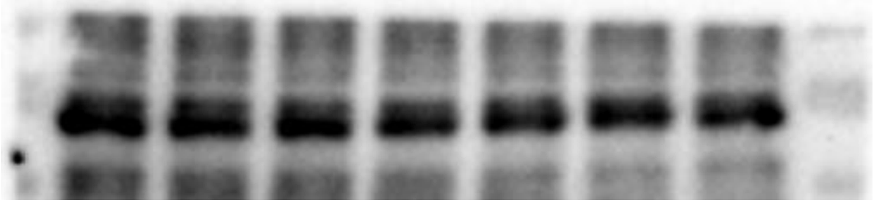

WCL---RLIM

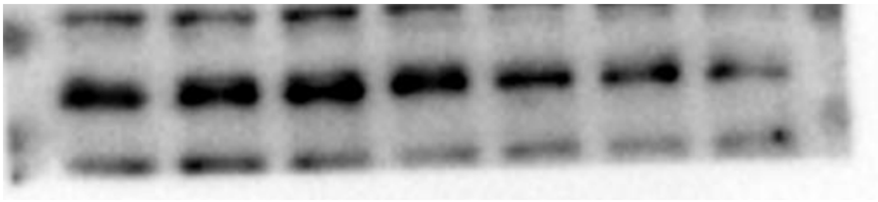

WCL---Tubulin

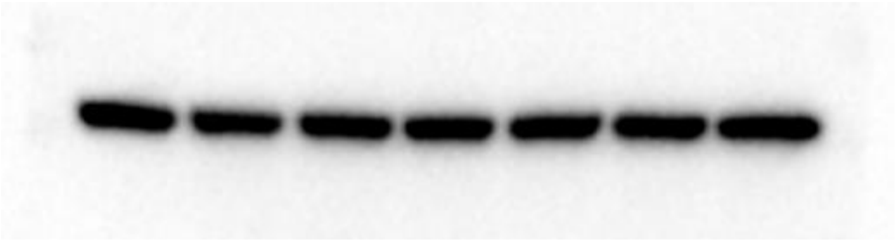

Figure 5H:

Cytomembrane---SLC7A11

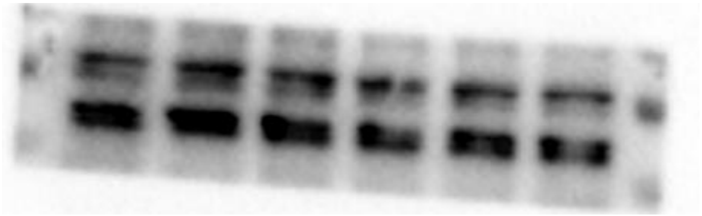

Cytomembrane---NaKATPase

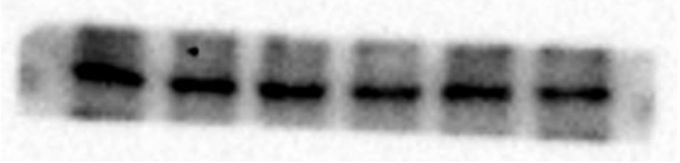

Cytoplasm---SLC7A11

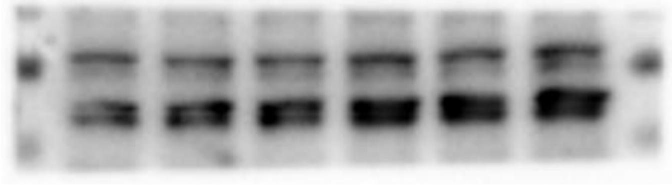

Cytoplasm---VINCULIN

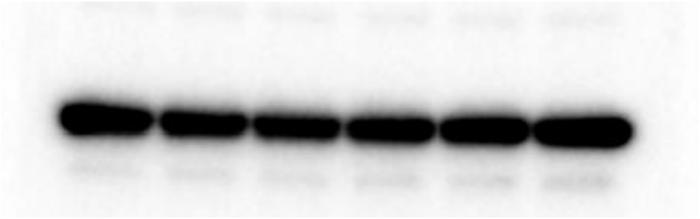

WCL---SLC7A11

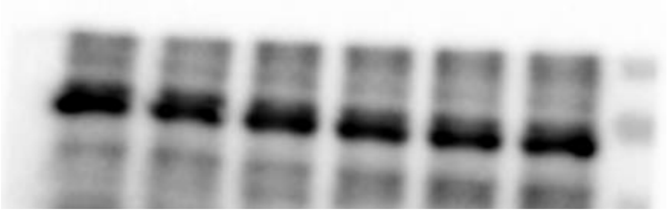

WCL---RLIM

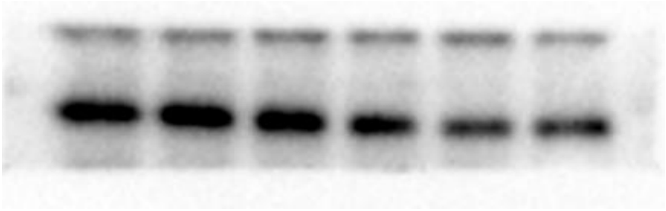

WCL---Tubulin

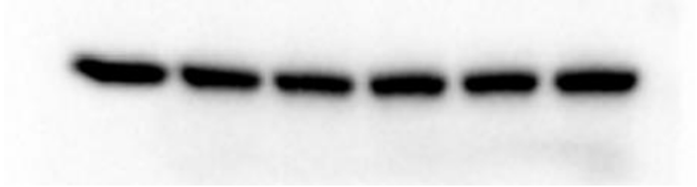

Figure 5J:

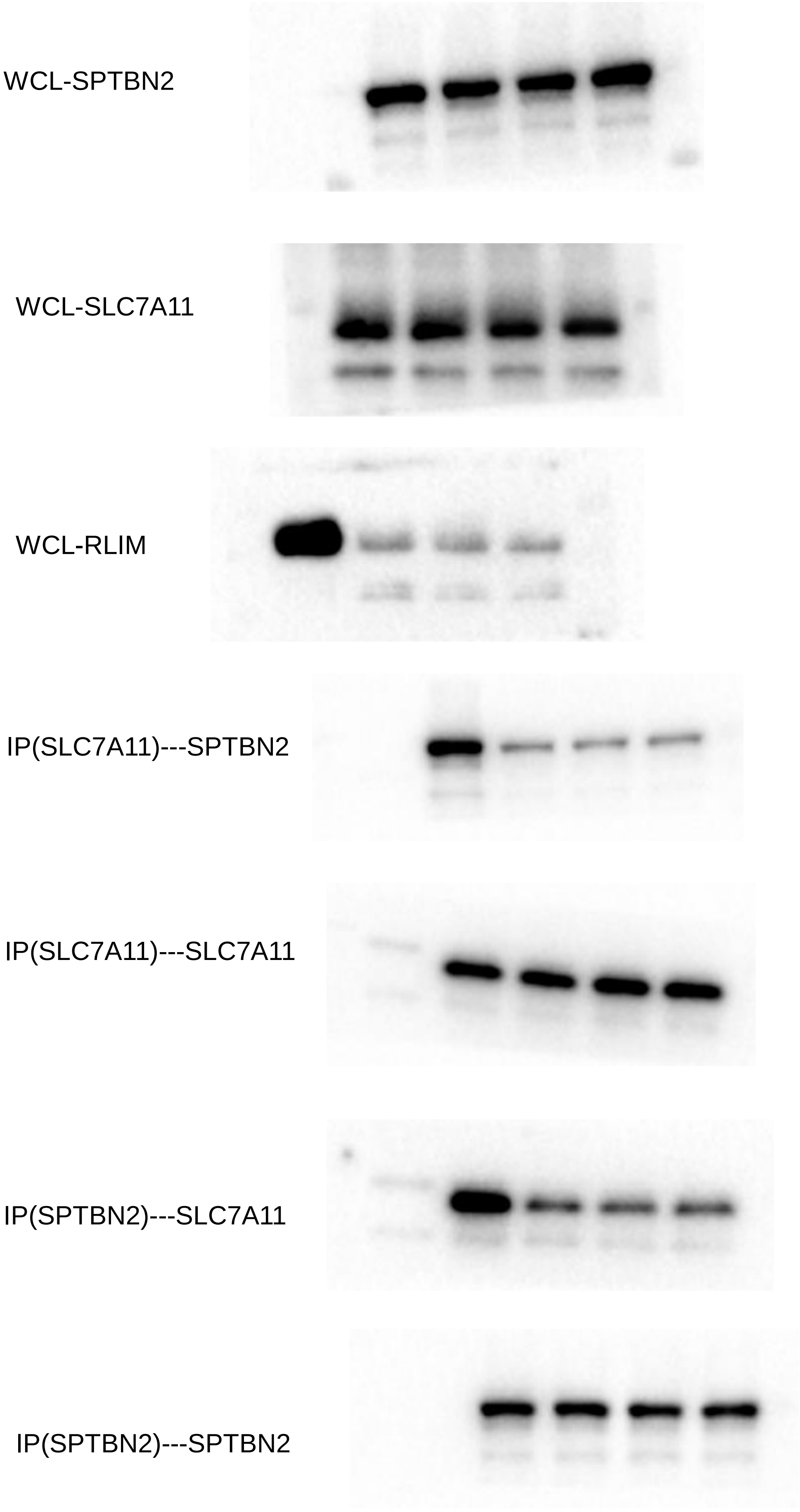
WCL-SPTBN2
WCL-SLC7A11
WCL-RLIM
IP(SLC7A11)---SPTBN2
IP(SLC7A11)---SLC7A11
IP(SPTBN2)---SLC7A11
IP(SPTBN2)---SPTBN2

Figure 6P:

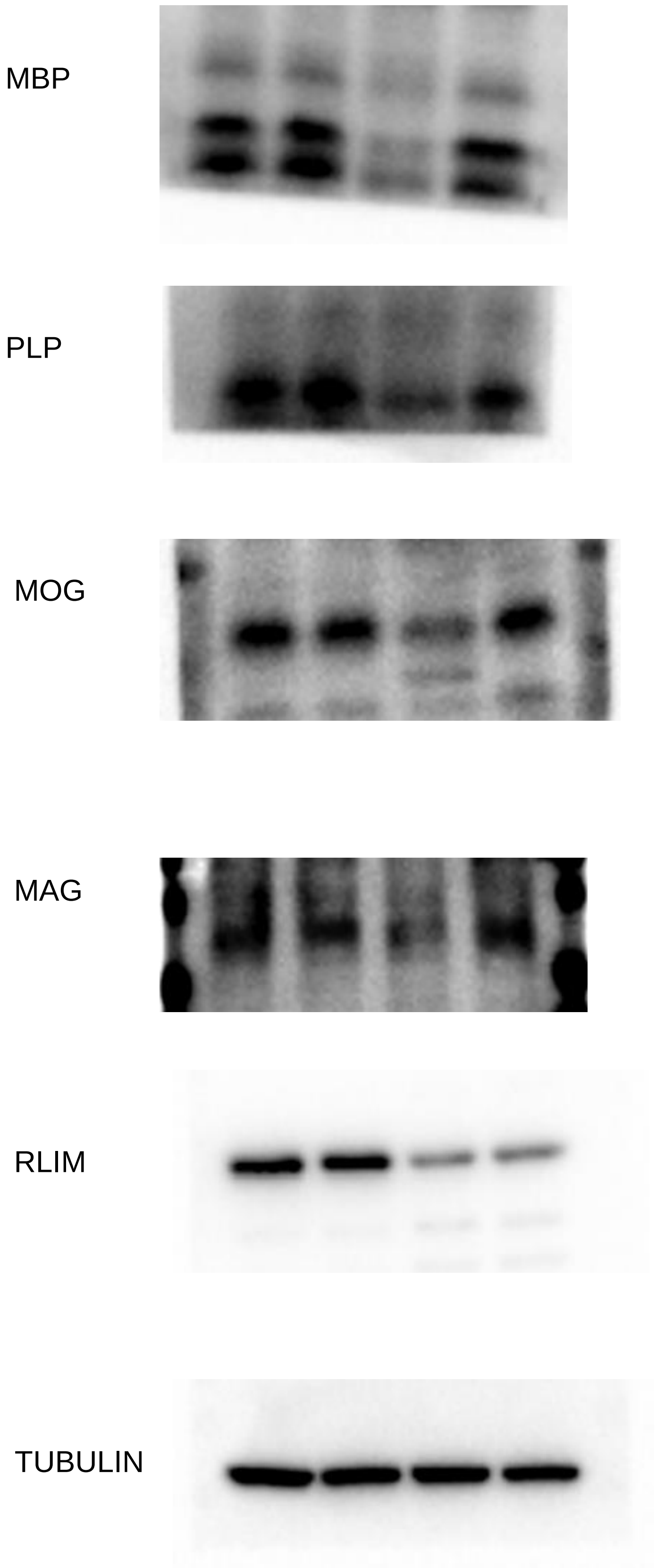

Figure 7A:

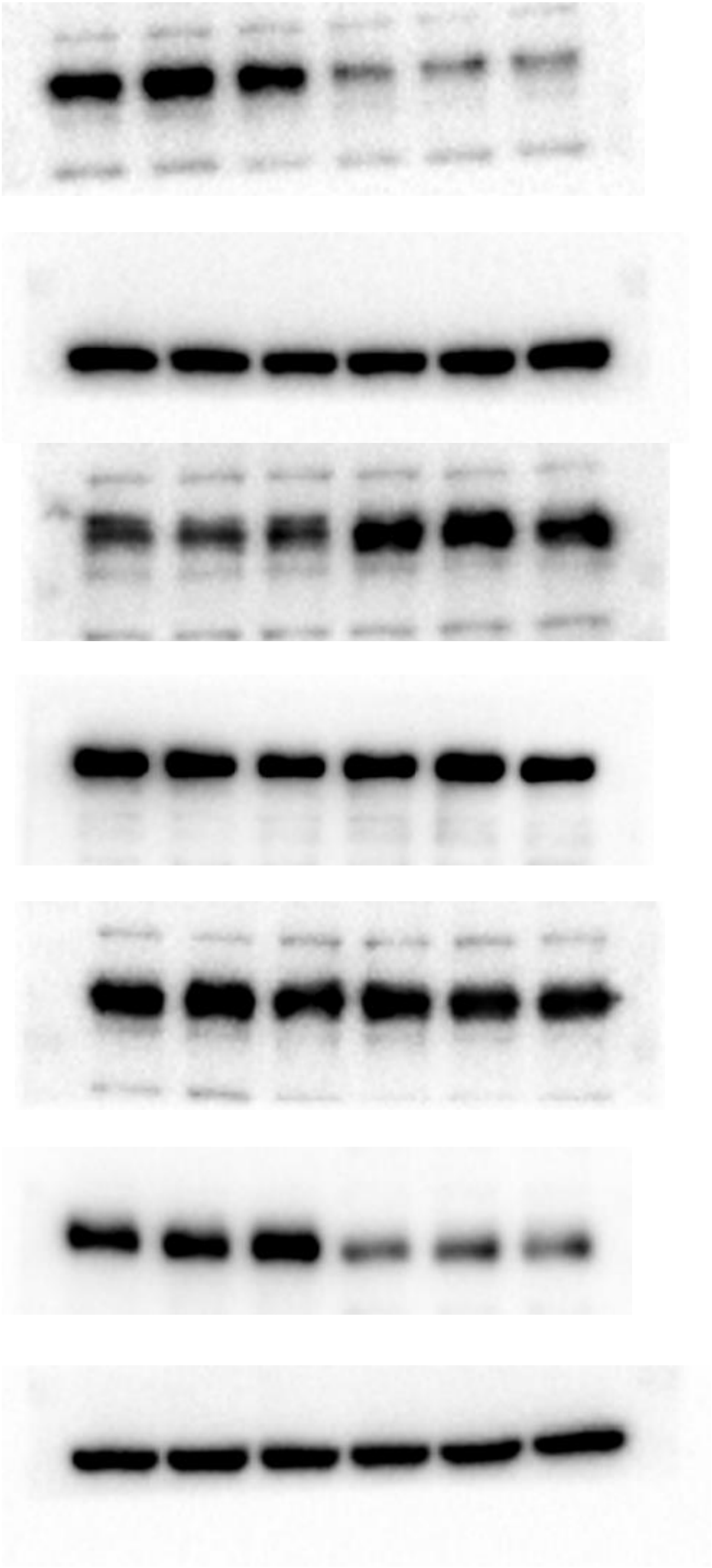

Cytomemebrane---SLC7A11

Cytomemebrane---NaKATPase

Cytoplasm---SLC7A11

Cytoplasm---VINCULIN

WCL---SLC7A11

WCL---RLIM

WCL---TUBULIN

Figure 7F:

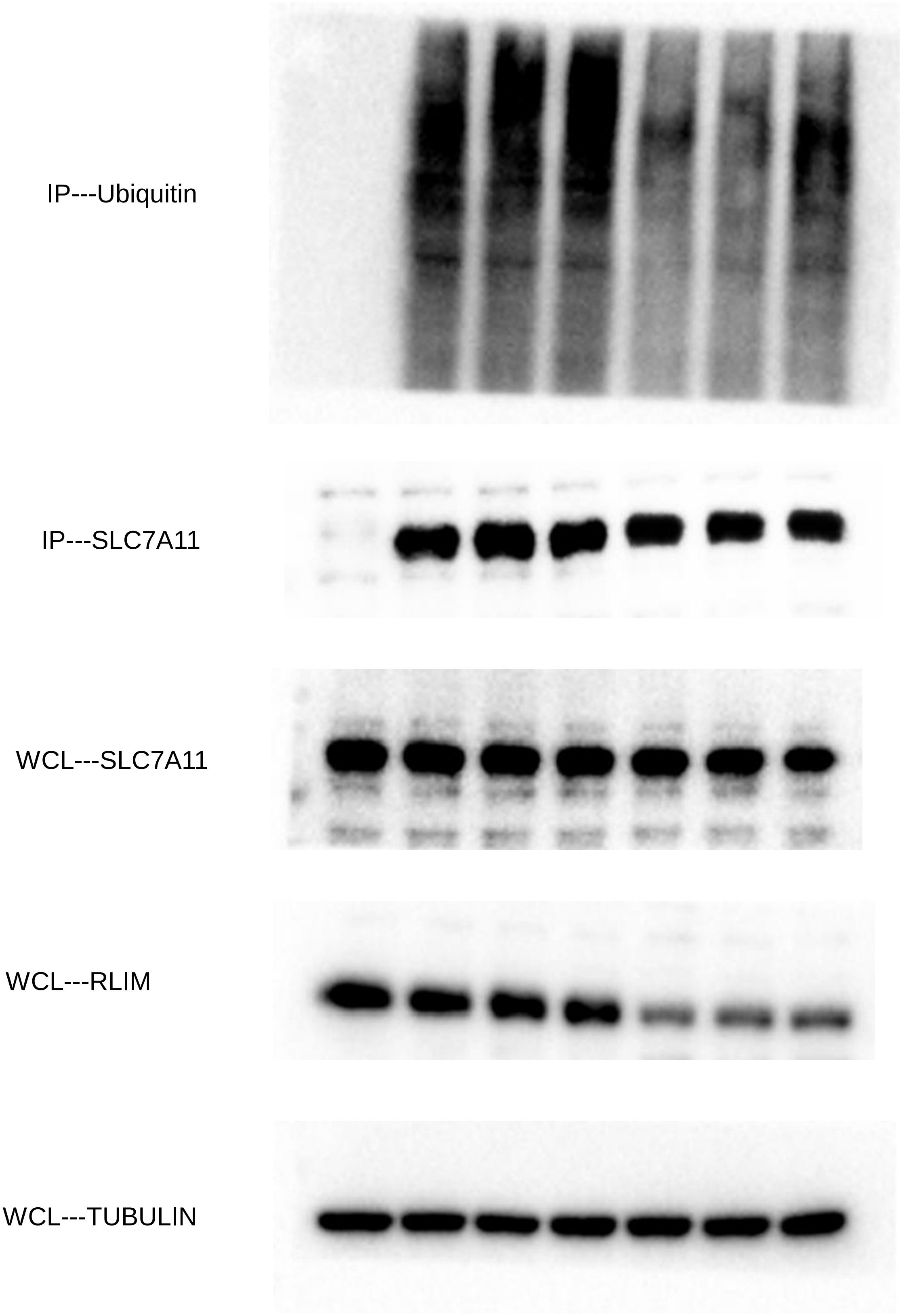

Figure S8F:

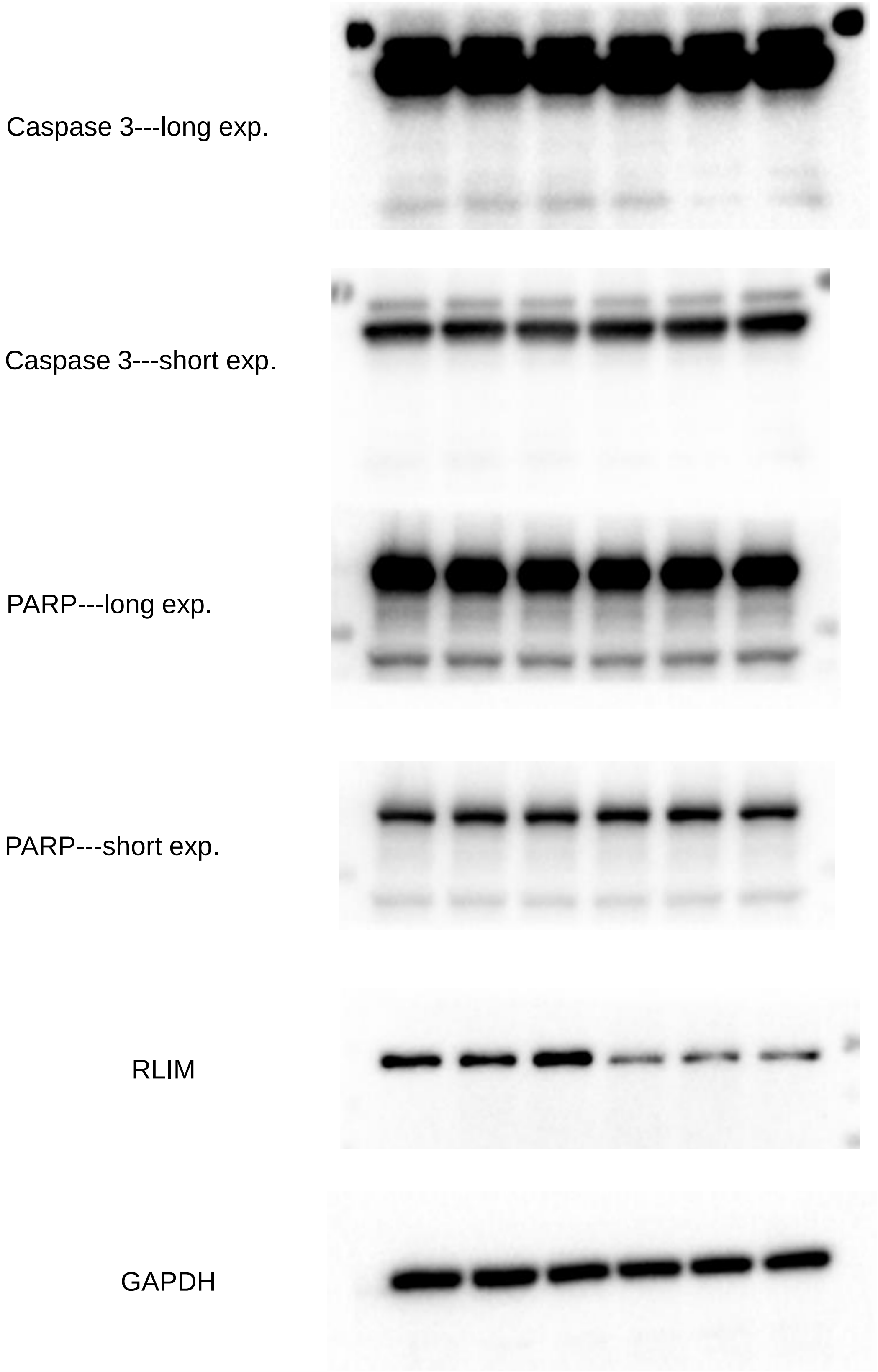
Caspase 3---long exp.
Caspase 3---short exp.
PARP---long exp.
PARP---short exp.
RLIM
GAPDH

Figure S8H:

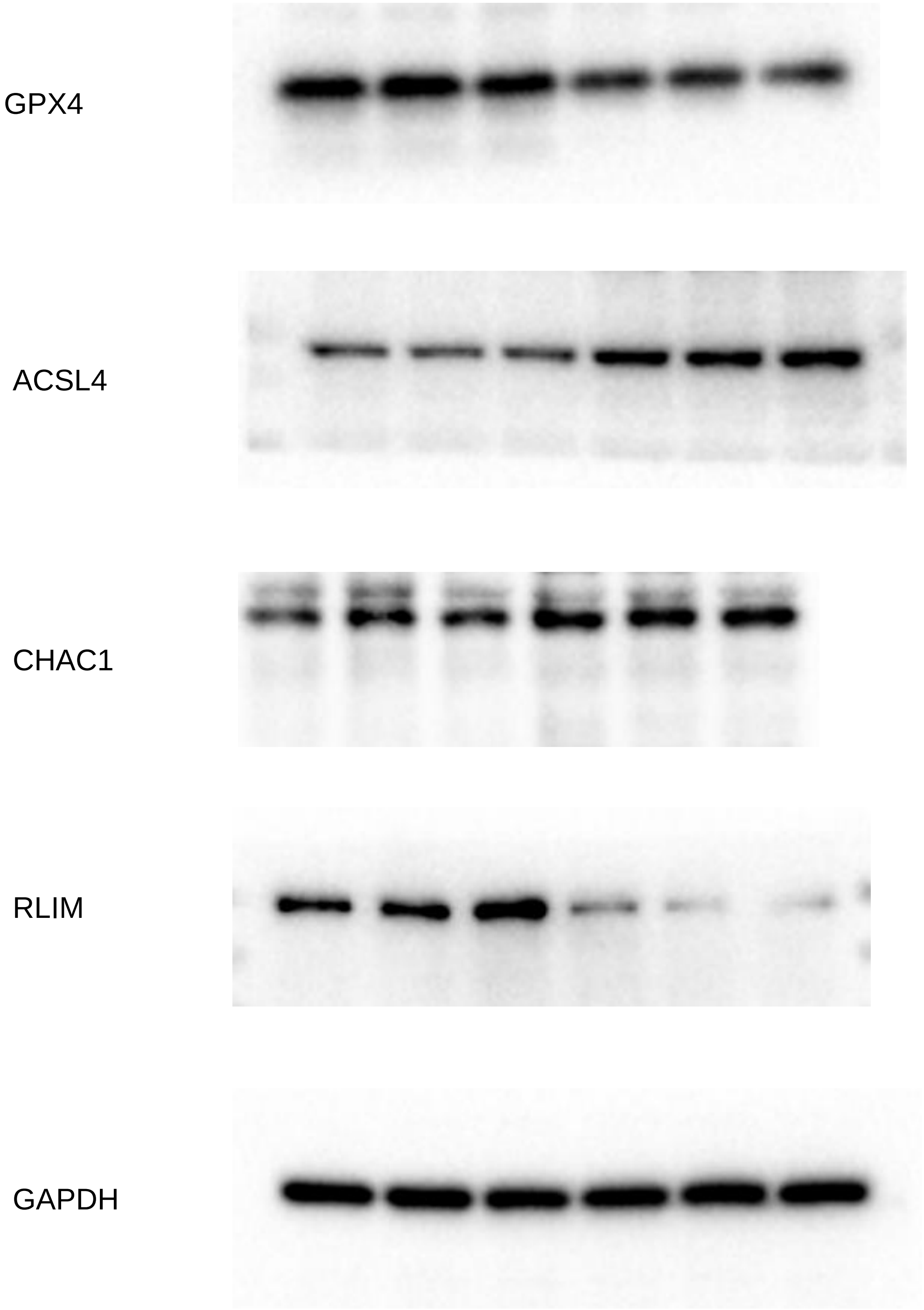

Figure S10A:

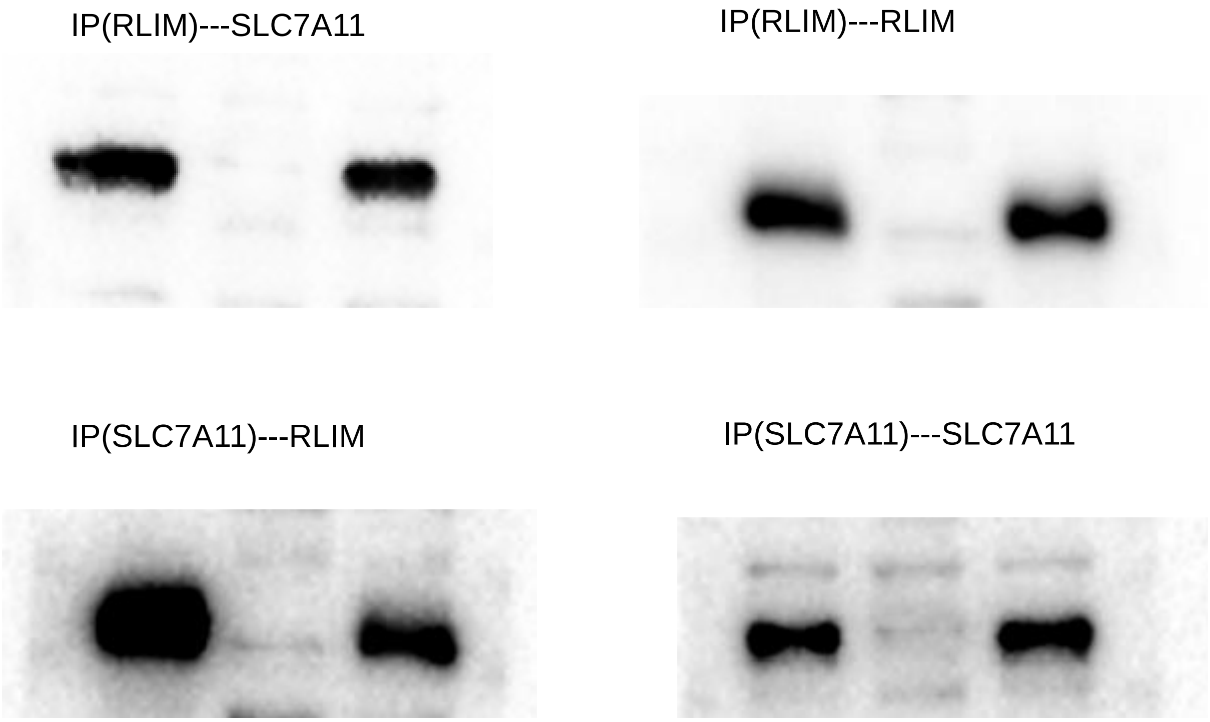
IP(RLIM)---SLC7A11
IP(RLIM)---RLIM
IP(SLC7A11)---RLIM
IP(SLC7A11)---SLC7A11

Figure S10B:

WCL---SLC7A11

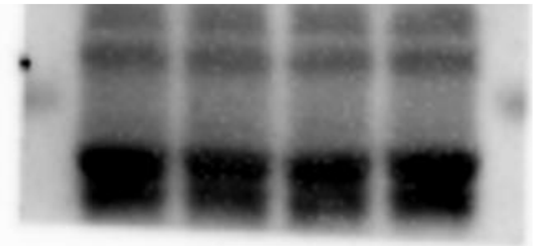

WCL---RLIM

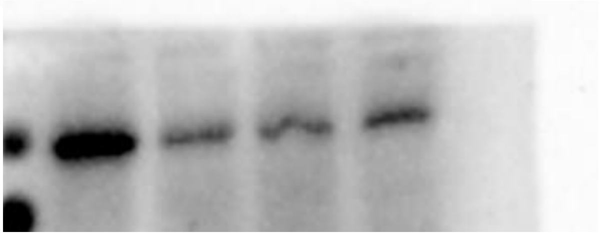

WCL---TUBULIN

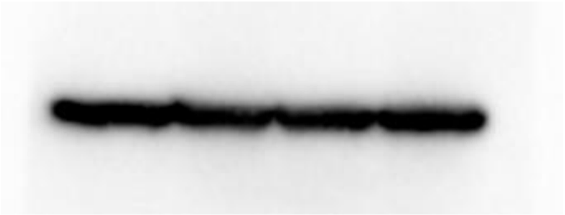

WCL---Ubiquitin

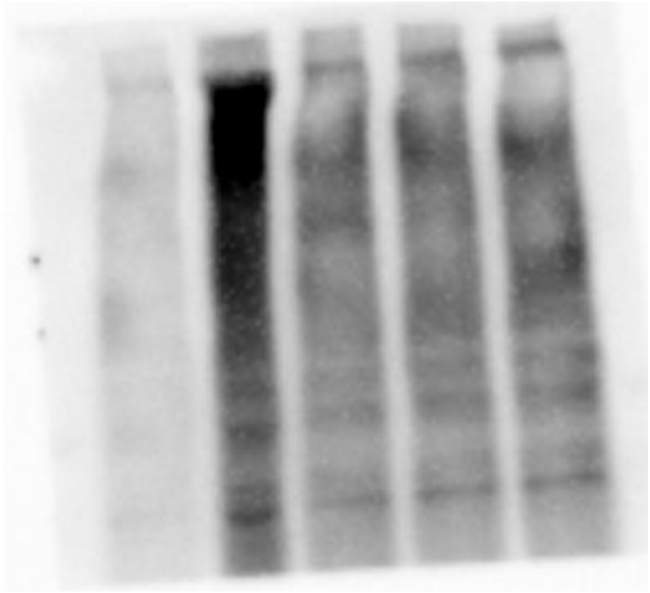

WCL---SLC7A11

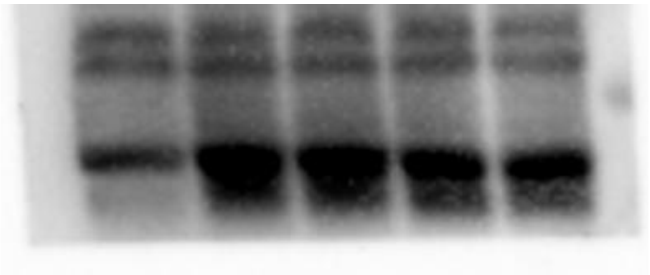

Figure S10D:

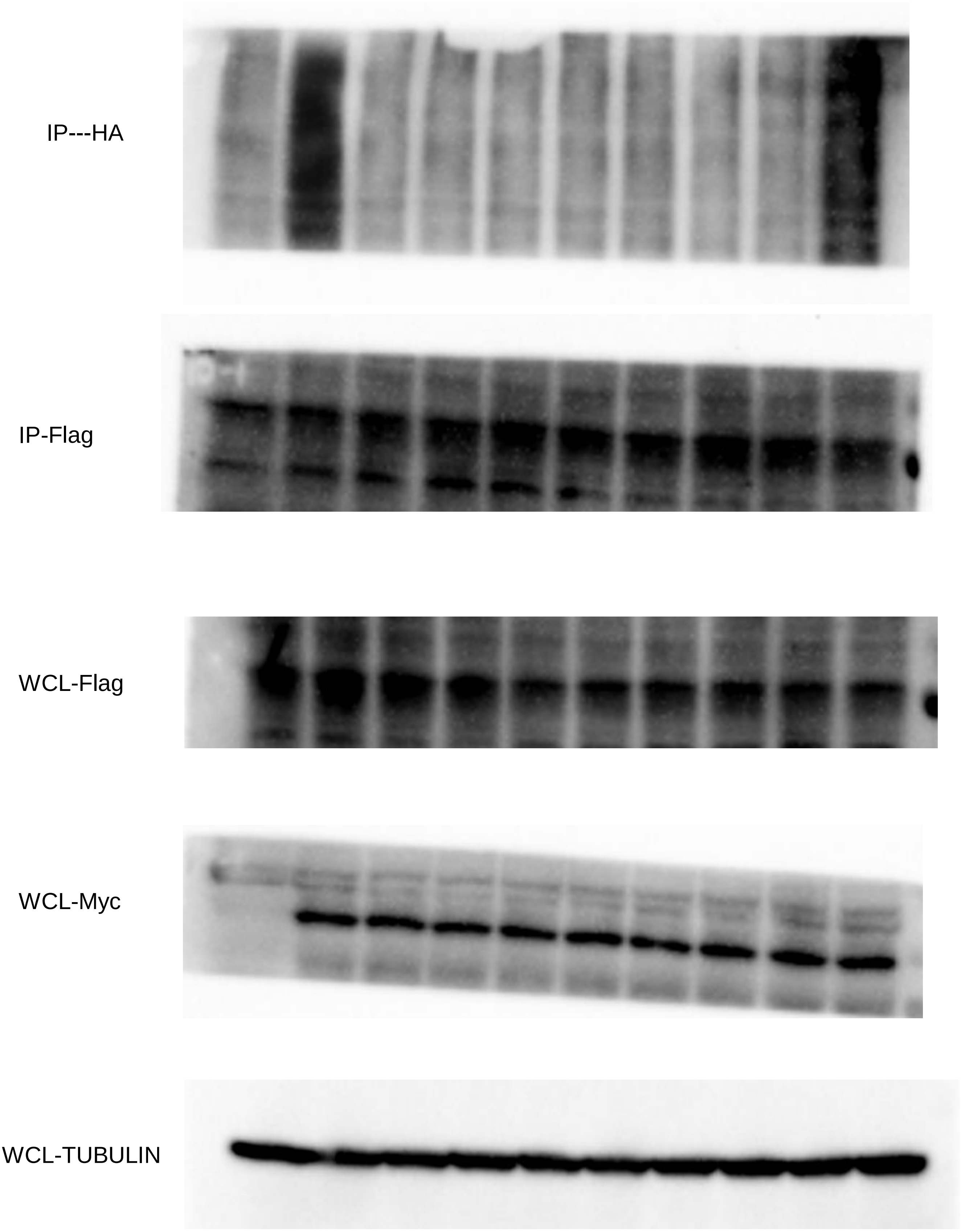

Figure S10E:

IP---HA

IP---Flag

WCL---Flag

WCL---Myc

WCL---Tubulin

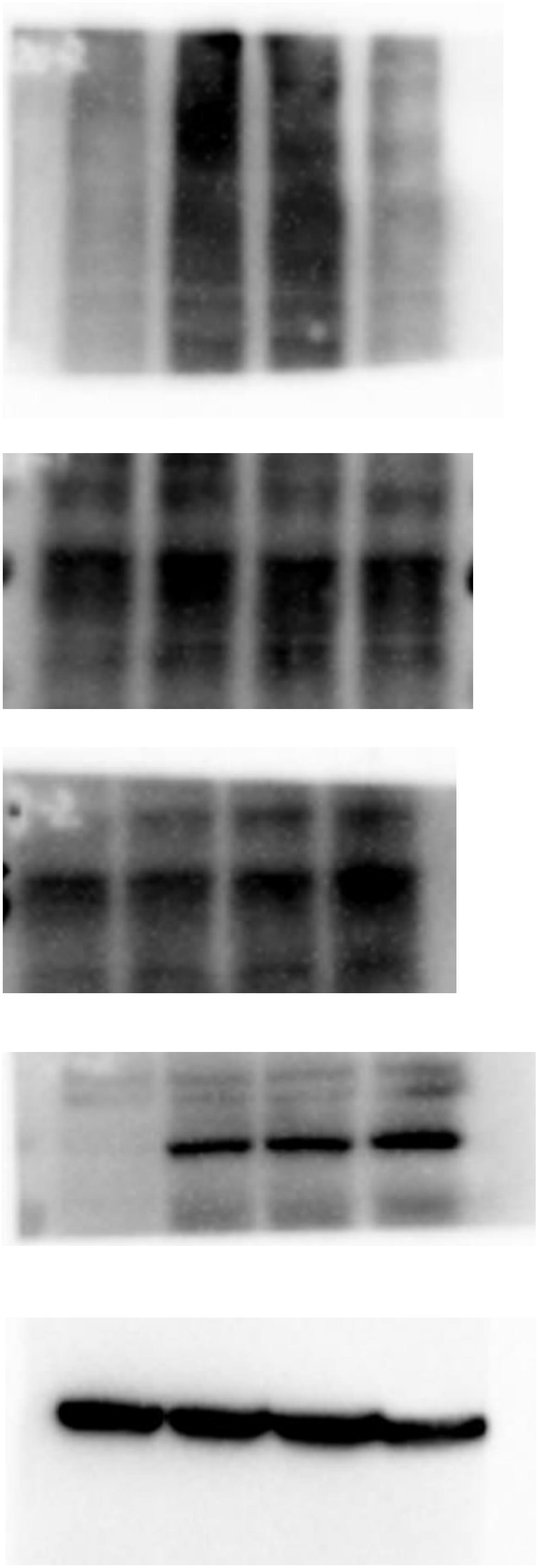

Figure S10F:

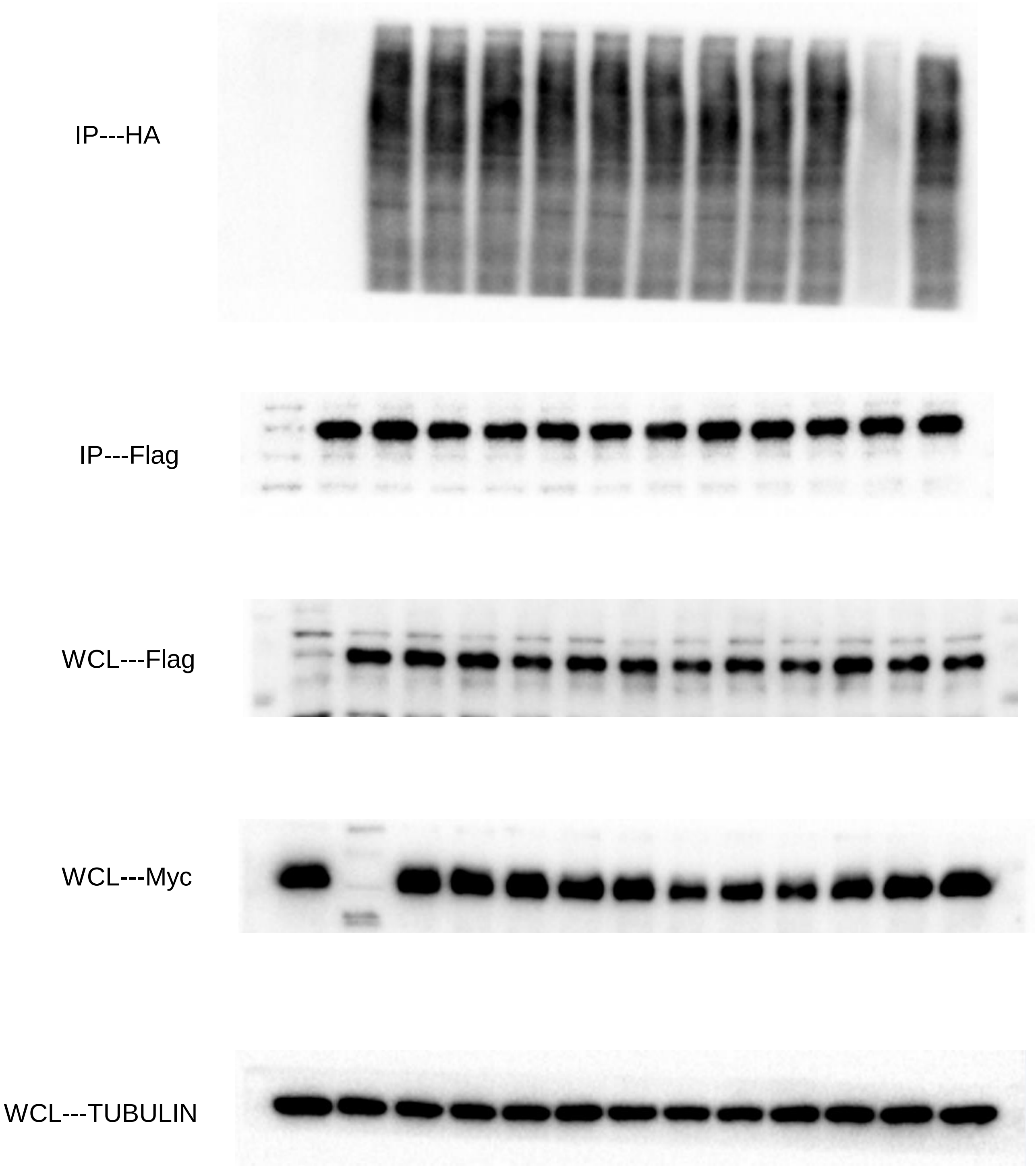

Figure S10H:

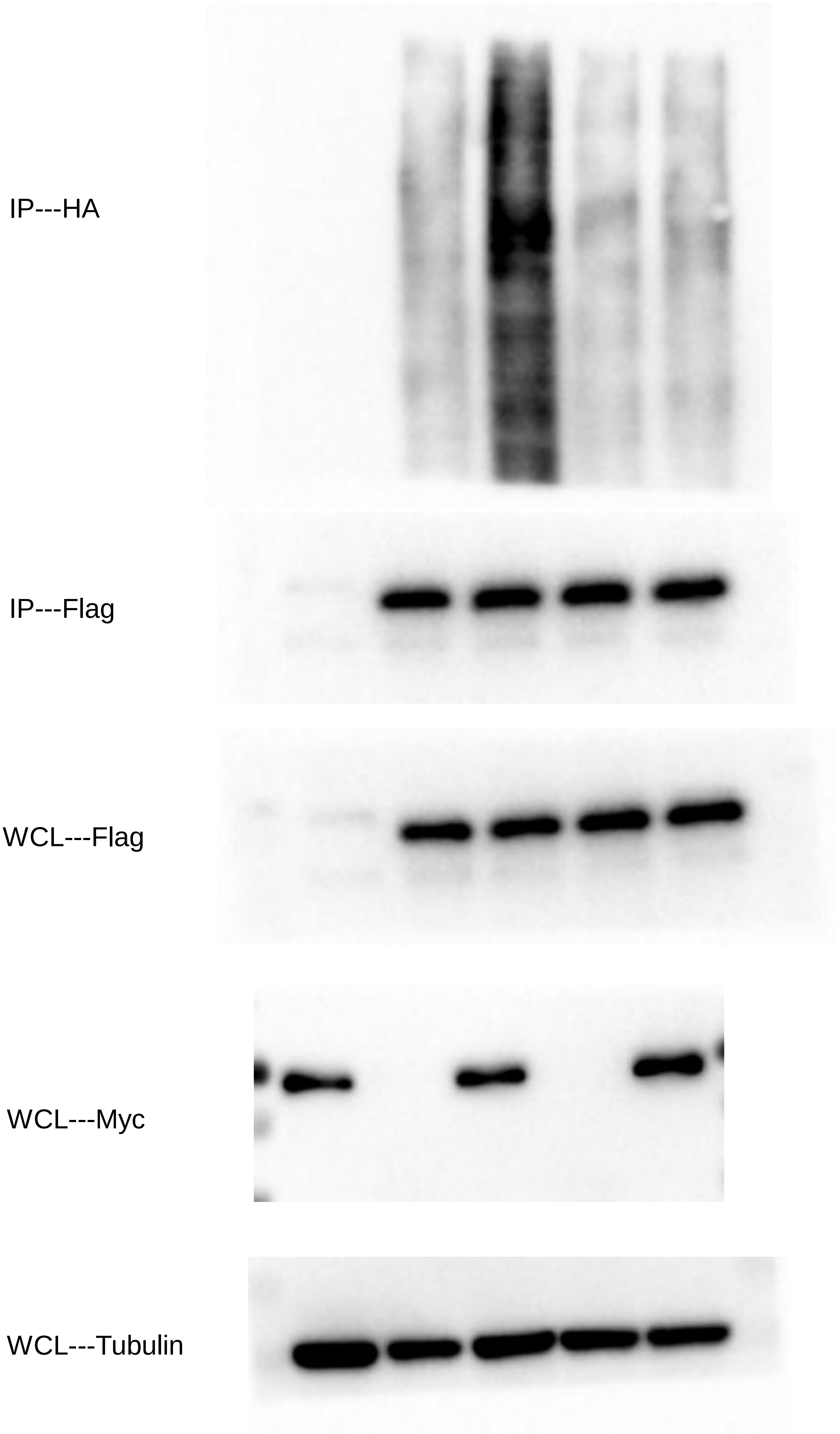

Figure S11A:

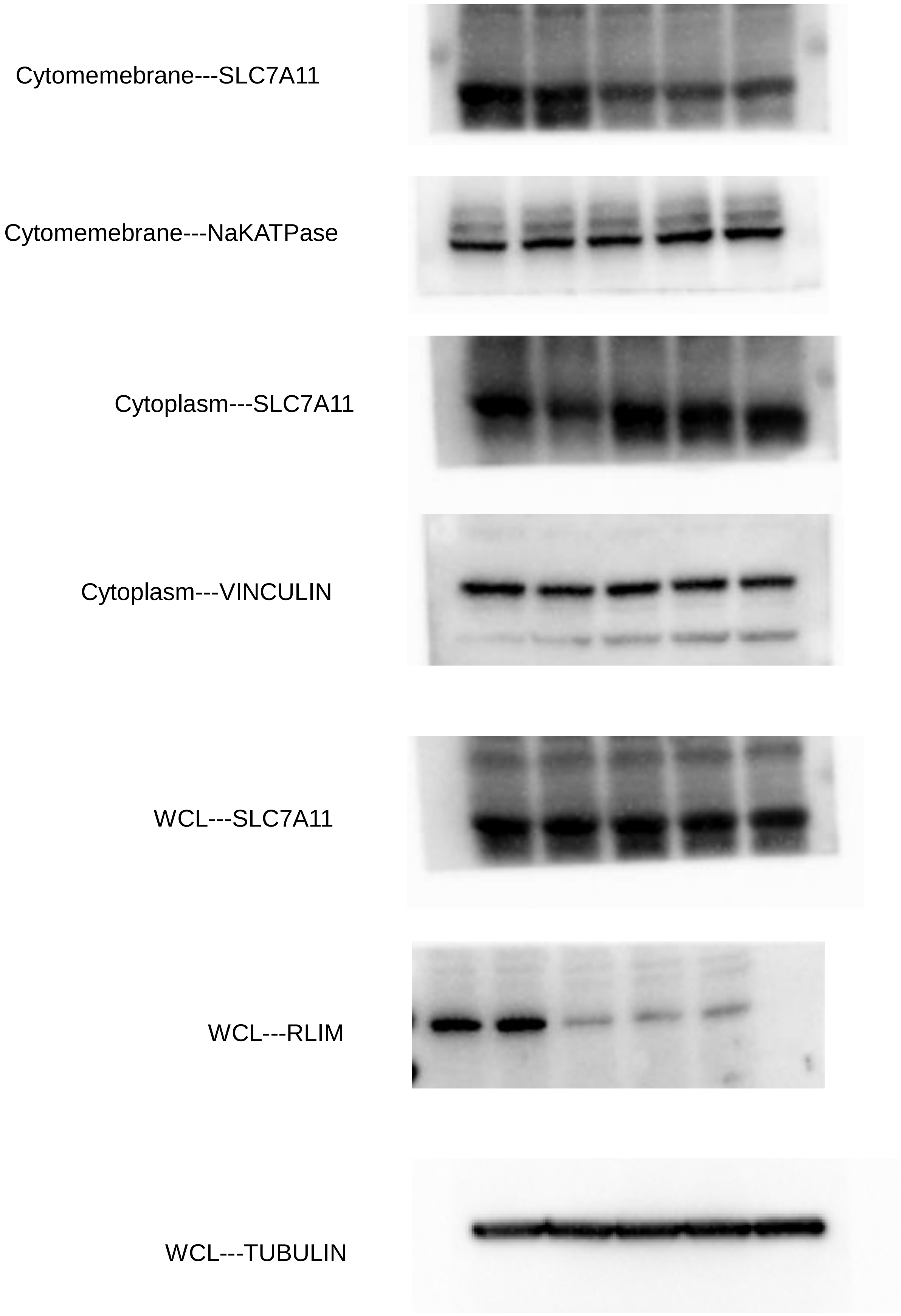
Cytomemebrane---SLC7A11
Cytomemebrane---NaKATPase
Cytoplasm---SLC7A11
Cytoplasm---VINCULIN
WCL---SLC7A11
WCL---RLIM
WCL---TUBULIN

Figure S11E:

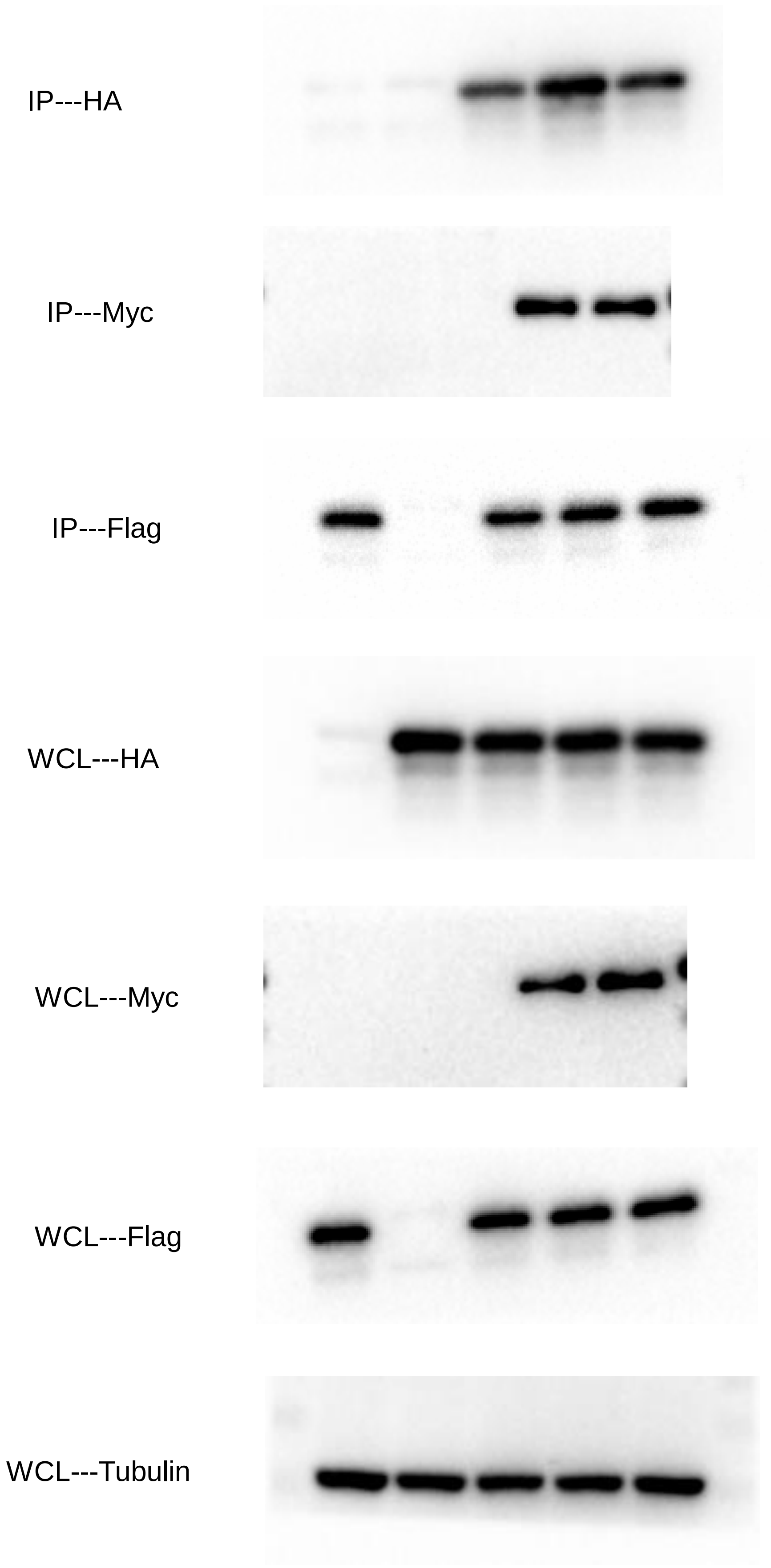

Figure S11G:

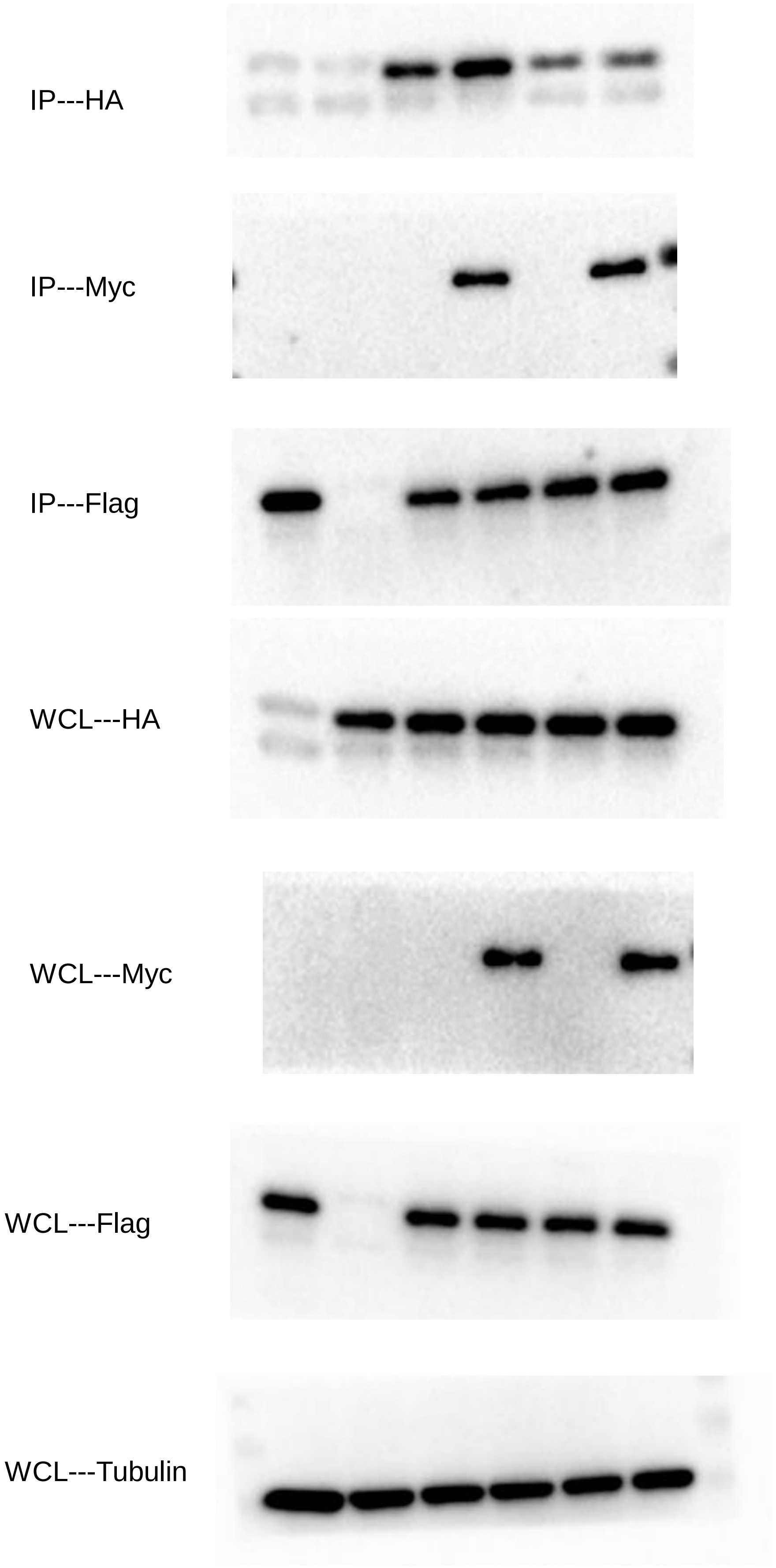

Figure S13A:

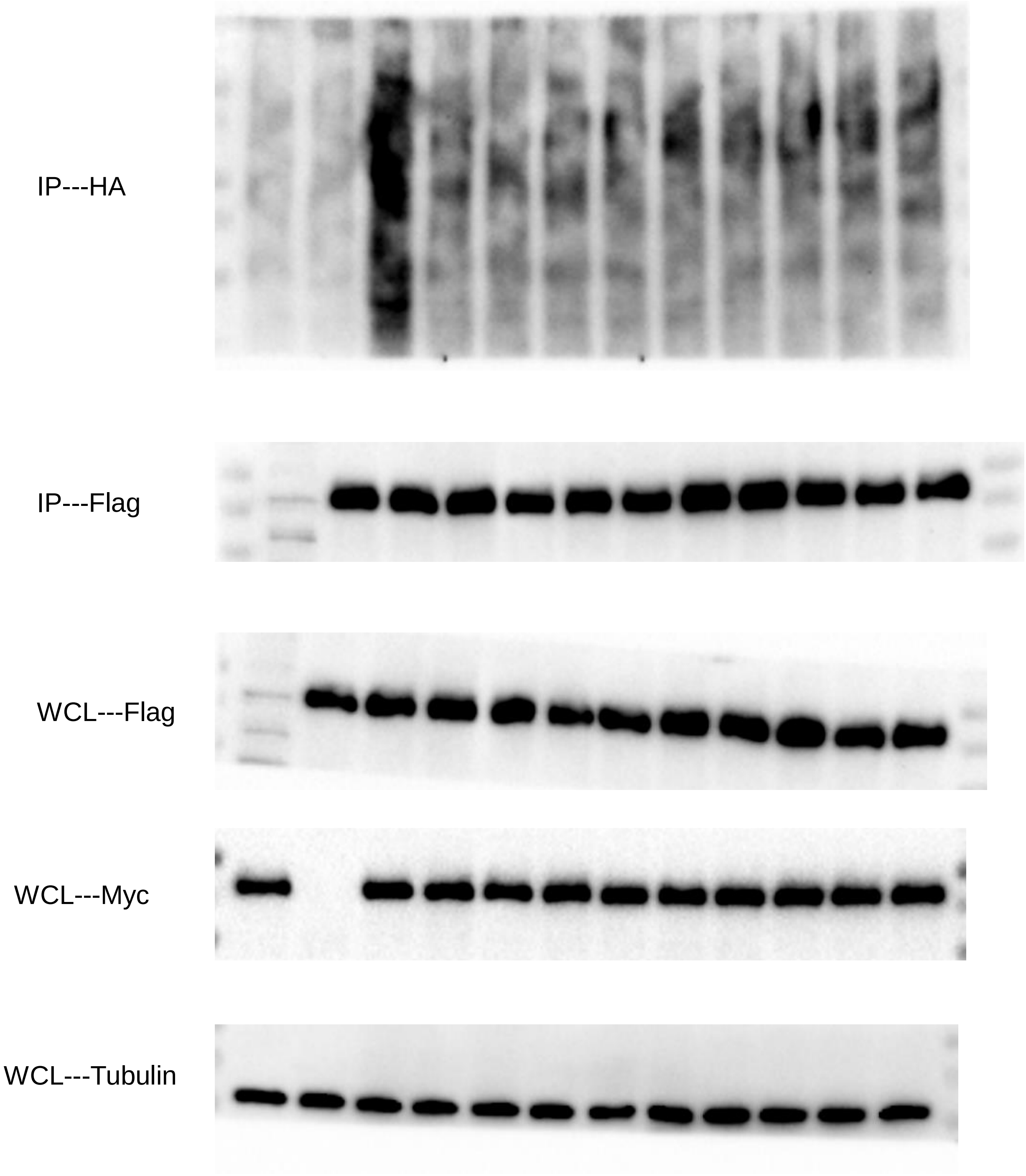

Figure S13C:

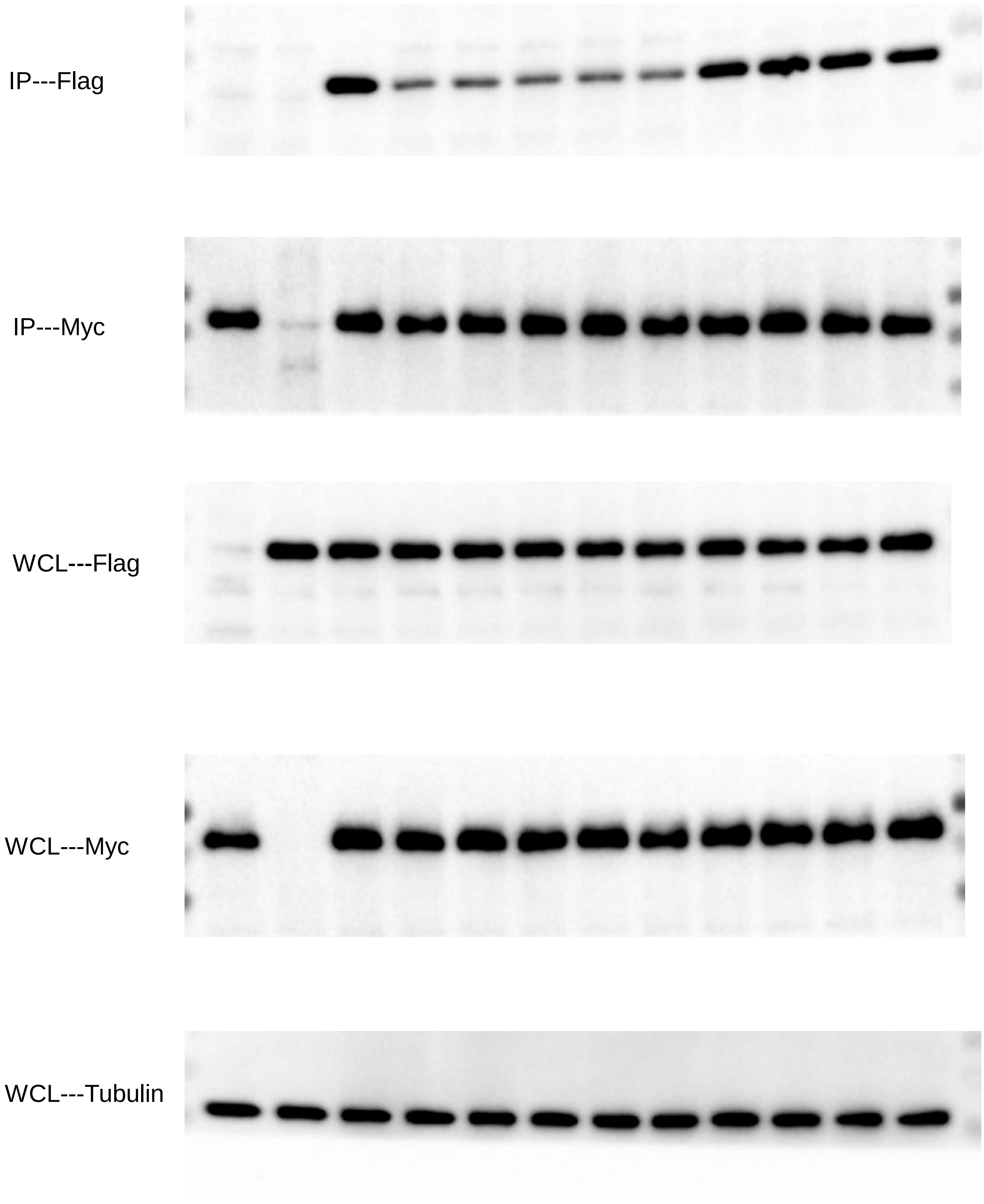

Supplement: Supplementary file 2 — Supporting File 2: advs76315‐sup‐0002‐Data.zip. [file ADVS-9999-e76315-s005.zip › Original Western Blot Images.pdf]
